# The mediating role of the kynurenine pathway in longitudinal associations between dietary intake and quality of life in colorectal cancer survivors up to 12 months posttreatment

**DOI:** 10.1002/ijc.70363

**Published:** 2026-02-24

**Authors:** Daniëlle D. B. Holthuijsen, Judith J. M. Rijnhart, Eline H. van Roekel, Martijn J. L. Bours, Per M. Ueland, Stéphanie O. Breukink, Maryska L. G. Janssen‐Heijnen, Joop L. Konsten, Eric T. P. Keulen, Adrian McCann, Stefanie Brezina, Biljana Gigic, Jennifer Ose, Matty P. Weijenberg, Simone J. P. M. Eussen

**Affiliations:** ^1^ Department of Epidemiology, CARIM Cardiovascular Research Institute Maastricht Maastricht University Maastricht The Netherlands; ^2^ Department of Epidemiology, GROW Research Institute for Oncology and Reproduction Maastricht University Maastricht The Netherlands; ^3^ College of Public Health University of South Florida Tampa Florida USA; ^4^ Bevital AS Bergen Norway; ^5^ Department of Surgery Maastricht University Medical Centre+ Maastricht The Netherlands; ^6^ NUTRIM School of Nutrition and Translational Research in Metabolism Maastricht University Medical Centre+ Maastricht The Netherlands; ^7^ Department of Clinical Epidemiology VieCuri Medical Centre Venlo The Netherlands; ^8^ Department of Surgery VieCuri Medical Centre Venlo The Netherlands; ^9^ Department of Internal Medicine and Gastroenterology Zuyderland Medical Centre Sittard‐Geleen Geleen The Netherlands; ^10^ Center for Cancer Research Medical University of Vienna Vienna Austria; ^11^ Department of General Visceral and Transplantation Surgery Heidelberg University Hospital Heidelberg Germany; ^12^ Department of Population Health Sciences University of Utah Salt Lake City Utah USA; ^13^ Huntsman Cancer Institute Salt Lake City Utah USA; ^14^ Department of Information and Communication, Faculty for Media, Information and Design University of Applied Sciences and Arts Hannover Germany; ^15^ Department of Epidemiology, CAPHRI Care and Public Health Research Institute Maastricht University Maastricht The Netherlands

**Keywords:** colorectal cancer survivorship, dietary intake, health‐related quality of life, mediation analysis, plasma kynurenines

## Abstract

Previous research has revealed associations between diet and health‐related quality of life (HRQoL), between diet and kynurenine pathway (KP) metabolites (kynurenines), and between kynurenines and HRQoL in colorectal cancer (CRC) survivors. We examined if kynurenines mediate longitudinal associations between diet and HRQoL in CRC survivors up to 12‐months posttreatment. Repeated measurements were performed in 209 stage I–III CRC survivors. Diet was assessed by seven‐day dietary records. Plasma kynurenines were analyzed using LC–MS/MS. HRQoL outcomes were assessed through the validated EORTC QLQ‐C30. We used confounder‐adjusted multilevel parallel‐multiple mediator models with all kynurenines simultaneously and single mediator models with established KP ratios to estimate total (*c*:diet‐HRQoL), direct (*c′*:diet‐HRQoL), metabolite‐specific indirect (*ab*: diet‐metabolite‐HRQoL), and total indirect (*ab*:diet‐metabolites‐HRQoL) effects. Higher carbohydrate intake was associated with worse role functioning, while higher fiber, alcohol, and zinc intake, and better adherence to the Dutch Healthy Diet (DHD) recommendations were associated with better physical and role functioning (*c*‐path). Associations of fiber and DHD with HRQoL remained statistically significant after controlling for all KP metabolites (*c′*‐path). All kynurenines simultaneously only mediated associations of higher carbohydrate intake with worse role functioning, and of higher alcohol intake with better physical functioning (*ab*). The kynurenic acid‐to‐quinolinic acid (KA/QA) ratio and hydroxykynurenine ratio (HKr) significantly mediated associations of carbohydrate, protein, fat, alcohol, magnesium, and zinc intake, and adherence to DHD recommendations, with physical and role functioning (*ab*). In conclusion, while all kynurenines simultaneously did not mediate diet‐HRQoL associations, the KA/QA ratio and HKr did mediate several associations within the first year after CRC treatment.

## What's New?

Many colorectal cancer survivors experience reduced health‐related quality of life (HRQoL), often lasting years after diagnosis. Healthy dietary patterns and even individual macro‐ and micronutrients, however, can potentially improve HRQoL. Here, the authors investigated the kynurenine pathway (KP), which is upregulated by stress and inflammation, as a potential mediator between diet and HRQoL in colorectal cancer survivors. Although KP did not overall impact HRQoL, the kynurenic acid‐to‐quinolinic acid ratio and hydroxykynurenine ratio were found to mediate several diet–HRQoL associations, including those involving carbohydrates, alcohol, and zinc. The findings offer insight into metabolic mechanisms linking diet to improved HRQoL after colorectal cancer.

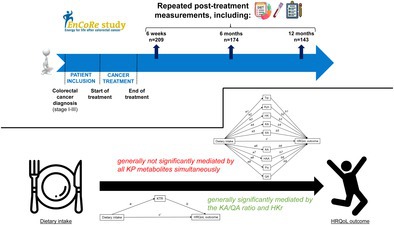

**Abbreviations**
AA: anthranilic acid
BMI: body mass index
CRC: colorectal cancer
DHD: Dutch Healthy Diet
HAA: 3‐hydroxyanthranilic acid
HK: 3‐hydroxykynurenine
HKr: hydroxykynurenine ratio (3‐hydroxykynurenine: [kynurenic acid + xanthurenic acid + 3‐hydroxyanthranilic acid + anthranilic acid])
HRQoL: health‐related quality of life
IQR: interquartile range
KA: kynurenic acid
KA/QA: kynurenic acid‐to‐quinolinic acid ratio
KTR: kynurenine‐to‐tryptophan ratio
Kyn: kynurenine
LC/MS–MS: liquid‐chromatography tandem‐mass spectrometry
MVPA: moderate‐to‐vigorous physical activity
Pic: picolinic acid
QA: quinolinic acid
SQUASH: Short QUestionnaire to ASsess Health‐enhancing physical activity
Trp: tryptophan
WCRF/AICR: World Cancer Research Fund/American Institute for Cancer Research
XA: xanthurenic acid

## 1 INTRODUCTION

Advances in colorectal cancer (CRC) screening, diagnosis, and treatment have led to an increase in the number of CRC survivors.^1^ The 5‐year survival rate after diagnosis and treatment of stage I–III CRC is 70%–90% nowadays, resulting in a growing global CRC survivor population of more than 5.25 million survivors in 2020.^2^ CRC survivors are being confronted with the side effects of both the disease and treatment, even years after diagnosis, which impedes their quality of life.^3^ In recent years, alongside the emphasis on improving cancer treatment, there has been an increasing focus on improving quality of life after cancer treatment.

A healthy lifestyle, including a healthy diet, has been linked to CRC survival and recurrence,^4^ but is also proposed to enhance post‐CRC quality of life.^5^ Evidence is emerging that meeting guidelines for cancer prevention may also prevent recurrence and improve post‐cancer quality of life.^6^, ^7^, ^8^ When focusing on the dietary recommendations, a longitudinal study among stage I–III CRC survivors in our research group revealed that higher intake of fiber, fruits, and vegetables, and lower consumption of meat, ultra‐processed foods, and sugary drinks were associated with better health‐related quality of life (HRQoL).^9^, ^10^ Next to these specific food component recommendations, a higher adherence to the Western diet was associated with worse HRQoL, while a diet rich in fruits and vegetables was associated with better HRQoL in a prospective cohort of CRC survivors.^11^ However, studies on the association between other dietary patterns or individual macro‐ and micronutrients with HRQoL outcomes in a CRC population are lacking, as well as underlying mechanisms that might explain these associations.

One potential underlying mechanism linking diet and HRQoL is the kynurenine pathway (KP). This pathway is upregulated by stress and inflammation and converts the essential amino acid tryptophan (Trp) into several downstream metabolites, known collectively as kynurenines (Supplementary Figure 1). The KP might be relevant due to its independent association with both dietary intake and HRQoL after cancer treatment. Recent studies, including our previous study, have shown that both macro‐ and micronutrient intake,^12^, ^13^ as well as adherence to dietary patterns,^14^, ^15^, ^16^, ^17^ were associated with alterations in KP in a variety of populations (i.e., healthy individuals, children with refractory epilepsy, CRC survivors, and patients at cardiovascular risk). In addition, a systematic review^18^ including several studies in patients with lung cancer, breast cancer, and various types of cancer, and in our recent longitudinal study in CRC survivors,^19^ reported on significant associations of tryptophan, kynurenic acid, xanthurenic acid, kynurenine‐to‐tryptophan ratio, kynurenic acid‐to‐quinolinic acid ratio, and hydroxykynurenine ratio with HRQoL.

The findings above suggest a potential mediating role of the KP in the association between dietary intake and HRQoL after cancer treatment, but such analyses have not been published yet. Therefore, we conducted a study to examine whether the KP mediates longitudinal associations between dietary intake and HRQoL outcomes up to 12 months posttreatment in CRC survivors. For this purpose, we used a holistic approach by examining all currently available KP metabolites simultaneously as mediators in parallel‐multiple mediator models, as well as by exploring established ratios that reflect the balance between KP metabolites as mediators in single mediator models.

## 2 MATERIALS AND METHODS

### 2.1 Study design and population

This study is embedded in the Energy for life after ColoRectal cancer (EnCoRe) study, an ongoing prospective cohort study looking into posttreatment lifestyle and health‐related outcomes in CRC survivors.^20^ Males and females aged 18 or older diagnosed with stage I‐III CRC at Maastricht University Medical Center+, VieCuri Medical Center, and Zuyderland Medical Center in The Netherlands were included since 2012. Exclusion criteria were stage IV CRC, not being a Dutch resident, unable to speak or read Dutch, or comorbidities that hamper successful participation (e.g., Alzheimer's disease).

### 2.2 Data collection

Data up to November 1, 2016, were used for the current study's analyses, as the metabolites of the KP had been analyzed for participants followed up until this specified date, providing data up to 12 months posttreatment. Trained research dietitians collected data and blood samples through home visits at 6 weeks, 6 months, and 12 months posttreatment. Participants with at least one posttreatment measurement of dietary intake, kynurenines, HRQoL, and relevant covariates were included in analyses (Supplementary Figure 2). Follow‐up participation rate exceeded 90%. The decline in participant numbers with increasing follow‐up time is mainly attributable to the fact that not all participants included at the moment of diagnosis had undergone all follow‐up measurements by the time of November 1, 2016.

### 2.3 Dietary intake

Participants filled in a structured dietary record on seven consecutive days at all posttreatment measurements. Information on consumed meals, foods, and beverages, with details on brand names, portion sizes, and preparation was collected in these seven‐day dietary records. Daily nutrient intake was calculated using food calculation software (Compl‐eat, Wageningen University, the Netherlands) based on the Dutch food compositions database (NEVO‐2011). Intake of macronutrients and micronutrients potentially involved in KP metabolism was estimated.^21^ In addition, adherence scores to the 2018 World Cancer Research Fund/American Institute for Cancer Research (WRCF/AICR) dietary recommendations^22^ and the 2015 Dutch Healthy Diet (DHD) recommendations^23^ were calculated according to methods previously published.^14^ A maximum score of 5 and 130 points for WCRF/AICR and DHD could be achieved, with higher scores indicating better adherence to healthy dietary habits.

### 2.4 Plasma kynurenines

Overnight fasting blood samples were collected at all posttreatment measurements according to standardized protocols. Samples were collected in 8.5 mL EDTA tubes. EDTA plasma samples were then divided in 500 μL aliquots and stored at −80°C within 4 h after blood was drawn until analysis. For analysis, samples were transported on dry ice to Bevital's laboratory in Bergen, Norway (www.bevital.no). Liquid chromatography–tandem mass spectrometry (LC–MS/MS) was used to analyze the nine metabolites of the KP, including tryptophan (Trp), kynurenine (Kyn), 3‐hydroxykynurenine (HK), kynurenic acid (KA), xanthurenic acid (XA), anthranilic acid (AA), 3‐hydroxyanthranilic acid (HAA), picolinic acid (Pic), and quinolinic acid (QA).^24^ The within‐day and between‐day variation coefficients for KP metabolites were previously determined to be in the ranges of 3.0% (for AA) to 9.5% (for XA) and 5.7% (for Trp) to 16.9% (for XA), respectively, and the limit of detection in the ranges of 0.4 nmol/L (for KA) to 400 nmol/L (for Trp).^24^

Three relevant ratios of individual kynurenine concentrations were calculated. The kynurenine‐to‐tryptophan ratio (KTR), a well‐established marker of inflammation and indoleamine 2,3‐dioxygenase (IDO) activation, was calculated by dividing the plasma concentration of Kyn (in nmol/L) by the plasma concentration of Trp (in μmol/L).^25^ The hydroxykynurenine ratio (HKr), a functional marker of vitamin B6 status, was calculated by dividing the plasma concentration of HK (in nmol/L) by the sum of the plasma concentrations of KA (in nmol/L), XA (in nmol/L), AA (in nmol/L), and HAA (in nmol/L).^26^ The kynurenic acid‐to‐quinolinic acid ratio (KA/QA), representing the ratio between the N‐methyl‐D‐aspartate receptor antagonist (KA; neuroprotective properties) and agonist (QA; neurotoxic properties), was calculated by dividing the plasma concentration of KA (in nmol) by the plasma concentration of QA (in nmol/L).^27^

### 2.5 Health‐related quality of life

Cancer‐specific HRQoL was assessed using the European Organization for the Research and Treatment of Cancer Quality of Life Questionnaire‐Core 30 (EORTC QLQ‐C30), which is a widely used and well‐validated questionnaire.^28^ The EORTC QLQ‐C30 contains 30 questions and includes five functioning scales (physical, role, cognitive, emotional, and social functioning), a global health/QoL scale, and a set of items for specific cancer‐/treatment‐related symptoms (fatigue, pain, nausea and vomiting, dyspnea, insomnia, appetite loss, constipation, diarrhea, and financial difficulties). To assess overall QoL, a summary score from the mean of 13 subscales (out of 15, excluding financial difficulty and global QoL) was calculated.^29^ All scores were linearly transformed to a scale of 0–100. The higher the score on a functional, global QoL or summary scale, the better the functioning or QoL. In the current analysis, physical functioning and role functioning were the primary outcomes of interest as the KP was particularly associated with these scales.^19^ Global QoL and the EORTC summary score were included as secondary outcomes in secondary analyses as well.

### 2.6 Covariates

In medical records, data on demographic variables, including age and sex, and clinical variables, including cancer stage, tumor site, and cancer treatment, were collected. Self‐reported data on the presence of comorbidities assessed with the Self‐Administered Comorbidity Questionnaire and the presence of a stoma assessed with the EORTC QLQ‐C29 were collected at all posttreatment measurements.^30^ The highest level of education attained was self‐reported only at time of diagnosis. Lifestyle‐related variables were assessed at all posttreatment measurements, including smoking status (never, former or current), body mass index (BMI) based on measurements of height (m) and weight (kg) by trained dietitians during home visits, total energy intake (kcal/day) assessed by seven‐day dietary records, self‐reported time spent in moderate‐to‐vigorous physical activity (MVPA, h/week) assessed with the Short QUestionnaire to ASsess Health‐enhancing Physical Activity,^31^ and objectively measured prolonged sedentary time (total time in hours/day spent in sedentary bouts of at least 30 minutes) using the validated tri‐axial MOX activity monitor worn 24 h a day for seven consecutive days.^32^ Plasma creatinine, an indicator of renal function, was measured using LC–MS/MS.^33^

### 2.7 Statistical analysis

Descriptive analyses were performed to describe the main characteristics of the study population, as well as the exposure, mediator, and outcome variables.

**FIGURE 1:**
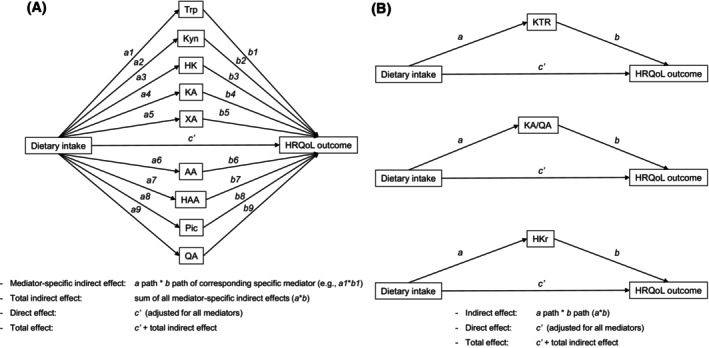
Schematic overview of the parallel‐multiple mediator model with all KP metabolites included simultaneously (A) and single mediator models with established KP ratios (B). In these models, dietary intake is modeled as exposure, all KP metabolites simultaneously or KP ratios are modeled as mediators, and HRQoL outcomes are modeled as outcome. Longitudinal associations between dietary intake and each of the metabolites of the KP as dependent variables were assessed (*a*‐paths), longitudinal associations of metabolites of the KP with HRQoL were assessed (*b‐*paths), and longitudinal associations of dietary intake with HRQoL were assessed (*c′*‐path; direct effect). Mediator‐specific interventional indirect effects were calculated as the product of the *a*‐ and *b*‐path corresponding to a specific mediator (a*b). Total indirect effects as the sum of all mediator‐specific interventional indirect effect estimates (sum of a*b). All linear mixed models included a random intercept but no models included a random slope, because the added model complexity caused the models to fail to converge. AA, anthranilic acid; HK, 3‐hydroxykynurenine; HKr, hydroxykynurenine ratio; HRQoL, health‐related quality of life; HAA, 3‐hydroxyanthranilic acid; Kyn, kynurenine; KA, kynurenic acid; KTR, kynurenine‐to‐tryptophan ratio; KA/QA, kynurenic acid‐to‐quinolinic acid ratio; Pic, picolinic acid; QA, quinolinic acid; Trp, tryptophan; XA, xanthurenic acid.

We used mediation analysis to determine whether the longitudinal association between dietary intake (i.e., macro‐ and micronutrient intake and adherence to dietary patterns) and HRQoL was (partially) mediated by the KP. We estimated interventional effects based on a parallel‐multiple mediator model which included all nine individual metabolites of the KP simultaneously as mediators (Figure 1).^34^, ^35^ Although theory suggests a causal ordering of the metabolites (Supplementary Figure 1), this causal sequence is not reflected in plasma, where all metabolites were measured simultaneously (thus in a cross‐sectional manner), and hence we used interventional effects in parallel‐multiple mediator models to relax the temporal ordering assumption among the mediators. We used linear mixed models to estimate the paths in the mediation models, as previously described in a similar study with fatigue as outcome.^36^ Repeated data on dietary intake, the KP, and HRQoL collected at 6 weeks, 6 months, and 12 months post‐treatment were included, and relations between these variables were modeled cross‐sectionally while considering repeated analyses in linear mixed models. All linear mixed models included a random intercept to address the clustered observations within individuals. While we initially explored the inclusion of random slopes to potentially enhance model fit, the models did not converge due to the increased complexity. Consequently, our final models did not include any random slopes.

In addition, we considered established and commonly used KP ratios (i.e., KTR, HKr, and KA/QA ratio) as mediators in single mediator models to investigate whether associations of dietary intake with HRQoL were mediated by these ratios, which reflect balances between KP metabolites.

We used the estimated *a*‐ and *b*‐paths to estimate the mediator‐specific interventional indirect effects and total indirect effects.^34^ The mediator‐specific interventional indirect effects were calculated as the product of the *a*‐ and *b*‐path corresponding to a specific mediator (a*b). These mediator‐specific interventional indirect effect estimates can be interpreted as the indirect effect of dietary intake on HRQoL through the specific metabolite and any metabolites causally preceding this metabolite.^34^ The total indirect effect was calculated as the sum of all mediator‐specific indirect effect estimates. The total indirect effect estimate can be interpreted as the indirect effect of dietary intake on HRQoL through all metabolites of the KP. The total effect (*c*‐path) was calculated as the sum of the direct effect (*c′*‐path) and total indirect effect (sum of all a*b). All identified paths, including the *a*‐path, the *b*‐path, the mediator‐specific interventional indirect effects, as well as the total indirect effect (a*b), the direct effect (*c′*‐path), and the total effect (*c*‐path), are reported. To address the skewed sampling distribution of the indirect effect estimates, confidence intervals (95%) for estimates of the indirect effects were estimated using Monte Carlo simulations with 20,000 draws.^37^, ^38^ Normal‐based 95% confidence intervals were estimated for the direct (*c′*‐path) and total (*c*‐path) effect estimates.^38^

In secondary analyses, we estimated the same associations but focusing on global QoL and the EORTC summary score as secondary outcomes.

Exposures were standardized by utilizing the mean of the standard deviations across all posttreatment measurements.

All models were adjusted for time‐invariant variables including age at enrolment (years), sex (male, female), and chemotherapy (yes, no), and time‐varying variables, including creatinine (μmol/L), number of comorbidities (0, 1, ≥2), presence of a stoma (yes, no), time since end treatment (weeks), BMI (kg/m^2^), MVPA (h/week), smoking status (current, former, never), educational level (low, medium, high), and prolonged sedentary time (h/day). Analyses with macronutrient intake as exposure were additionally adjusted for energy intake using the all‐components method, in which all sources of energy (e.g., carbohydrates, fats, proteins, fibers, and alcohol) were individually added to the model as continuous variables in kcal/day.^39^ Moreover, if plant‐based protein intake was the exposure, animal‐based protein and all other sources of energy, except protein (e.g., carbohydrates, fats, fibers, and alcohol), were included in the model as continuous variables in kcal/day. When dietary pattern scores (DHD score and WCRF/AICR dietary score) were the exposure, we additionally adjusted for energy intake using the standard method in which total energy intake as a continuous variable in kcal/day was added to the model.^39^ For analyses in which micronutrient intake was the exposure, we additionally adjusted for both alcohol intake (kcal/day) and total energy intake using the standard method by adding total energy intake as a continuous variable in kcal/d to the model.^39^

All analyses were carried out in R (version 3.4.1).^40^ The “lme4” package was used for linear mixed models and the “RMediation” package was used to estimate the confidence intervals for indirect, direct, and total effect estimates. The R scripts used for the analyses can be found in the Supplementary materials and methods. For all tests, statistical significance was set at *p* <.05.

## 3 RESULTS

### 3.1 Participant characteristics

Participant characteristics at 6 weeks (*n* = 209), 6 months (*n* = 174), and 12 months (*n* = 143) posttreatment are shown in Table 1. At 6 weeks posttreatment, the mean age was 66.1 (SD: 9.1) and more than two‐thirds were male (67.5%). Colon cancer was the most prevalent tumor site, diagnosed in 61.2% of the participants, while 38.8% of participants were diagnosed with rectal cancer. Most participants were diagnosed with stage III CRC (41.2%), while 34.5% were diagnosed with stage I and 24.4% with stage II. The majority of participants underwent surgery (88.5%), and 36.4% and 25.8% of participants received additional (neo‐)adjuvant chemotherapy and/or radiotherapy, respectively. Descriptives on exposure, mediator, and outcome data are presented in Table 2.

**TABLE 1:** Sociodemographic, clinical and lifestyle characteristics of participants included in the energy for life after ColoRectal cancer (EnCoRe) study at 6 weeks, 6 months and 12 months posttreatment with data on kynurenines (previously published in Holthuijsen et al.^36^).

	6 weeks posttreatment (*n* = 209)^a^	6 months posttreatment (*n* = 174)^a^	12 months posttreatment (*n* = 143)^a^
Sex (men), *n* (%)	141 (67.5)	111 (65.3)	99 (70.0)
Age (years), mean (SD)	66.1 (9.1)	66.7 (9.4)	66.4 (9.4)
Tumor site, *n* (%)			
Colon	128 (61.2)	109 (62.6)	85 (59.4)
Rectum	81 (38.8)	65 (37.4)	58 (40.6)
Cancer stage, *n* (%)			
Stage I	72 (34.5)	55 (31.6)	42 (29.4)
Stage II	51 (24.4)	43 (24.7)	43 (30.1)
Stage III	86 (41.2)	76 (43.7)	58 (40.6)
Cancer treatment, *n* (%)			
Surgery (yes)	185 (88.5)	158 (90.8)	126 (88.1)
Chemotherapy (yes)	76 (36.4)	67 (38.5)	50 (35.0)
Radiotherapy (yes)	54 (25.8)	47 (27.0)	42 (29.4)
Number of comorbidities, *n* (%)			
0 comorbidities	40 (19.1)	37 (21.3)	37 (26.1)
1 comorbidity	54 (25.8)	44 (25.3)	33 (23.2)
≥2 comorbidities	115 (55.0)	93 (53.5)	72 (50.7)
Stoma (yes), *n* (%)	63 (30.1)	31 (17.8)	20 (14.0)
BMI (kg m^2^), mean (SD)	27.7 (4.4)	28.3 (4.4)	28.3 (4.5)
Underweight: <18.5	1 (0.5)	0 (0.0)	1 (0.7)
Healthy weight: 18.5–24.9	63 (30.3)	39 (22.5)	31 (22.1)
Overweight: 25.0–29.9	87 (41.8)	77 (44.5)	63 (45.0)
Obese: ≥30	57 (27.4)	57 (32.9)	45 (32.1)
Educational level, *n* (%)			
Low	53 (25.4)	46 (26.6)	29 (20.4)
Medium	82 (39.2)	69 (39.9)	62 (43.7)
High	74 (35.4)	58 (33.5)	51 (35.9)
Smoking status, *n* (%)			
Never	70 (33.5)	58 (33.5)	43 (30.3)
Former	121 (57.9)	105 (60.7)	85 (59.9)
Current	18 (8.6)	10 (5.8)	14 (9.9)
MVPA (h/week), median (IQR)	7.5 (12.0)	9.4 (11.0)	9.8 (14.0)
Prolonged sedentary behavior (h/day), mean (SD)^b^	4.8 (2.4)	4.4 (1.9)	4.4 (1.7)
Total energy intake (kcal/day), mean (SD)	2130.5 (517.8)	1998.7 (466.8)	2038.8 (492.5)
Male	2312.3 (464.3)	2159.8 (427.7)	2189.8 (448.3)
Female	1750.5 (412.4)	1710.0 (395.3)	1696.6 (433.9)
Creatinine (μmol/L), median (IQR)	81.9 (21.2)	84.1 (22.3)	83.1 (22.5)

Abbreviations: BMI, body mass index; IQR, interquartile range; MVPA, moderate‐to‐vigorous physical activity; SD, standard deviation.

^a^ Numbers may not correspond to the total number of participants included at each time point due to missing data, and percentages may not add up to 100 due to rounding.

^b^ Total daily time spent in bouts of sedentary behavior of at least 30 minutes, based on accelerometer data.

**TABLE 2:** Macro‐ and micronutrient intake, dietary pattern scores, plasma concentrations of tryptophan, kynurenines, and ratios, and HRQoL outcomes of participants included in the Energy for life after ColoRectal cancer (EnCoRe) study at 6 weeks, 6 months, and 12 months posttreatment (partly previously published in Holthuijsen et al.^36^).

	6 weeks posttreatment (*n* = 209)	6 months posttreatment (*n* = 174)	12 months posttreatment (*n* = 143)
Macronutrient intake, mean (SD)			
Total protein (g/d–kcal/d)	79.6 (19.6)–318.6 (78.2)	75.3 (15.7)–301.1 (62.7)	76.7 (16.4)–306.9 (65.6)
Animal‐based protein (g/d–kcal/d)	47.9 (16.2)–191.6 (64.6)	44.6 (12.1)–178.3 (48.4)	45.5 (13.2)–182.1 (52.7)
Plant‐based protein (g/d–kcal/d)	31.8 (8.8)–127.0 (35.3)	30.7 (8.1)–123.0 (32.3)	31.3 (7.9)–125.3 (31.6)
Total carbohydrates (g/d–kcal/d)	229.3 (58.9)–917.2 (235.5)	211.7 (53.6)–846.8 (214.4)	218.2 (59.4)–872.6 (237.8)
Mono‐ and disaccharides (g/d–kcal/d)	94.0 (33.5)–375.9 (133.8)	82.3 (31.7)–329.1 (126.9)	84.9 (37.5)–339.5 (150.2)
Polysaccharides (g/d–kcal/d)	135.2 (36.5)–540.8 (146.0)	129.3 (34.0)–517.3 (136.1)	133.2 (34.6)–532.9 (138.3)
Total fat (g/d–kcal/d)	84.1 (25.2)–756.8 (227.0)	78.7 (22.0)–708.4 (198.2)	78.5 (21.7)–706.9 (195.3)
Saturated fat (g/d–kcal/d)	30.8 (10.4)–277.0 (94.0)	29.2 (9.6)–264.6 (86.3)	29.0 (9.9)–260.6 (89.0)
Unsaturated at (g/d–kcal/d)	45.8 (14.5)–412.1 (130.7)	42.5 (12.3)–380.4 (111.2)	42.7 (12.2)–384.2 (109.7)
Alcohol (g/d–kcal/d)	12.8 (16.7)–89.6 (116.2)	13.5 (19.8)–94.8 (138.9)	15.0 (19.8)–104.7 (138.5)
Fiber (g/d–kcal/d)	21.3 (6.0)–42.6 (12.1)	20.9 (6.3)–41.8 (12.5)	21.4 (6.1)–42.7 (12.3)
Micronutrient intake, mean (SD)			
Vitamin B2 (mg/d)	1.3 (0.4)	1.2 (0.4)	1.2 (0.4)
Vitamin B6 (mg/d)	1.9 (0.6)	1.8 (0.7)	1.8 (0.6)
Magnesium (mg/d)	320.3 (83.8)	306.1 (82.1)	313.0 (84.7)
Zinc (mg/d)	9.9 (2.6)	9.5 (2.2)	9.5 (2.3)
Dietary pattern scores, mean (SD)			
Dutch Healthy Diet index	66.9 (16.1)	69.3 (14.8)	67.0 (15.1)
WCRF dietary score	1.9 (0.7)	2.1 (0.7)	2.0 (0.7)
Plasma kynurenines, median (IQR)			
Tryptophan (μmol/L)	66.4 (12.1)	67.5 (15.9)	68.2 (14.8)
Kynurenine (μmol/L)	1.9 (0.6)	1.8 (0.6)	1.8 (0.6)
3‐Hydroxykynurenine (nmol/L)	49.9 (25.2)	47.5 (22.5)	46.5 (21.9)
Kynurenic acid (nmol/L)	53.8 (22.7)	59.6 (28.2)	58.7 (32.6)
Xanthurenic acid (nmol/L)	13.1 (9.2)	15.2 (9.2)	14.6 (10.5)
Anthranilic acid (nmol/L)	16.0 (6.7)	16.5 (7.5)	16.3 (8.1)
3‐Hydroxyanthranilic acid (nmol/L)	42.9 (18.4)	42.6 (17.4)	42.8 (18.1)
Picolinic acid (nmol/L)	33.5 (15.5)	36.0 (16.1)	35.0 (17.7)
Quinolinic acid (nmol/L)	515.0 (303.0)	493.5 (278.0)	456.0 (270.0)
KTR	28.7 (9.3)	27.2 (8.4)	27.6 (8.0)
HKr	0.39 (0.18)	0.35 (0.15)	0.34 (0.12)
KA/QA ratio	0.10 (0.06)	0.12 (0.06)	0.13 (0.06)
Health‐related quality of life: EORTC QLQ‐C30 (range 0–100), mean (SD)			
Physical functioning	78.3 (18.7)	82.5 (18.1)	83.8 (19.0)
Role functioning	72.3 (26.9)	83.1 (21.4)	85.4 (22.4)
Global QoL	74.6 (18.4)	78.9 (16.5)	78.4 (17.1)
Summary score of HRQoL	84.7 (12.7)	88.7 (10.2)	88.8 (12.8)

Abbreviations: EORTC QLQ‐C30, European Organization for the Research and Treatment of Cancer Quality of Life Questionnaire; HKr, hydroxykynurenine ratio; HRQoL, health‐related quality of life; IQR, interquartile range; KTR, kynurenine‐to‐tryptophan ratio; KA/QA, kynurenic acid‐to‐quinolinic acid; QoL, quality of life; SD, standard deviation; WCRF, World Cancer Research Fund.

### 3.2 Multilevel multiple‐parallel mediator analysis with diet, the KP, and physical functioning

None of the total effect estimates and direct effect estimates of associations of intake of total carbohydrates, mono‐ and disaccharides, polysaccharides, total protein, animal‐based protein, plant‐based protein, total fat, saturated fat, unsaturated fat, vitamin B2, vitamin B6, magnesium, and zinc, as well as the WCRF dietary score, with physical functioning were statistically significant (Table 3). We observed that higher intake of fiber (total effect: standardized *β*: 2.28; 95% CI: 0.68, 3.88) and alcohol (total effect: 2.09; 0.45, 3.72), as well as a higher adherence to the DHD recommendations (total effect: 2.43; 1.20, 3.67), were statistically significantly associated with better physical functioning (Table 3). Even after adjustment for all kynurenines, the association between higher fiber intake (direct effect: 2.20; 0.62, 3.78) and higher DHD score (direct effect: 2.45; 1.24, 3.66) with improved physical functioning remained statistically significant (Table 3).

**TABLE 3:** Analysis of all KP metabolites as mediators in the longitudinal association of macro‐ and micronutrient intake and adherence to dietary patterns with physical functioning and role functioning; parallel‐multiple mediator model.

			Physical functioning (0–100)					Role functioning (0–100)				
Exposure	Mediator	*a*‐path	*b*‐path	Mediator‐specific interventional indirect effect (a*b)	Total indirect effect	Direct effect	Total effect	*b*‐path	Mediator‐specific interventional indirect effect (a*b)	Total indirect effect	Direct effect	Total effect
Macronutrient intake												
Total carbohydrates *(per SD = 230 kcal/day)*	Trp	−1.65^a^	−0.01	0.01 (−0.26, 0.30)	−0.60 (−1.31, 0.10)	−0.18 (−2.09, 1.73)	−0.78 (−2.68, 1.12)	0.11	−0.18 (−0.77, 0.27)	**−1.27 (−2.58, −0.13)**	−3.12(−6.50, 0.25)	**1.02 (0.10, 2.05)**
	Kyn	0.05	−0.18	−0.01 (−0.31, 0.29)				−6.58	−0.30 (−1.03, 0.15)			
	HK	0.79	−0.07^a^	−0.06 (−0.35, 0.20)				−0.06	−0.05 (−0.40, 0.22)			
	KA	−2.89^a^	0.11^a^	−0.31 (−0.83, 0.01)				0.14	−0.41 (−1.20, 0.09)			
	XA	−1.47^a^	0.19	−0.28 (−0.85, 0.17)				0.38	−0.57 (−1.58, 0.19)			
	AA	−0.53	0.01	−0.00 (−0.14, 0.12)				0.07	−0.04 (−0.33, 0.19)			
	HAA	−0.91	−0.00	0.00 (−0.18, 0.18)				−0.02	0.02 (−0.28, 0.35)			
	Pic	−2.45^a^	−0.03	0.07 (−0.20, 0.38)				−0.06	0.16 (−0.31, 0.71)			
	QA	23.06	−0.00	−0.03 (−0.25, 0.15)				0.00	0.09 (−0.18, 0.50)			
Mono‐ and disaccharides *(per SD = 137 kcal/day)*	Trp	−0.90	−0.01	0.01 (−0.16, 0.19)	−0.29 (−0.80, 0.23)	−0.05 (−1.51, 1.40)	−0.34 (−1.81, 1.12)	0.11	−0.10 (−0.49, 0.16)	−0.60 (−1.53, 0.26)	−1.74 (−4.32, 0.83)	−2.34 (−4.90, 0.22)
	Kyn	0.01	−0.16	−0.00 (−0.16, 0.15)				−6.56	−0.10 (−0.52, 0.21)			
	HK	−1.51	−0.07^a^	0.11 (−0.07, 0.36)				−0.06	0.09 (−0.12, 0.42)			
	KA	−2.19^a^	0.11^a^	−0.24 (−0.62, 0.01)				0.14	−0.31 (−0.91, 0.07)			
	XA	−1.02^a^	0.19	−0.19 (−0.61, 0.11)				0.38	−0.39 (−1.12, 0.13)			
	AA	−0.20	0.01	−0.00 (−0.08, 0.07)				0.07	−0.01 (−0.19, 0.13)			
	HAA	−0.23	−0.00	0.00 (−0.10, 0.10)				−0.02	0.00 (−0.17, 0.20)			
	Pic	−1.95^a^	−0.03	0.06 (−0.16, 0.30)				−0.06	0.12 (−0.25, 0.56)			
	QA	23.11	−0.00	−0.03 (−0.22, 0.13)				0.00	0.09 (−0.15, 0.44)			
Polysaccharides *(per SD = 140 kcal/day)*	Trp	−1.14	−0.01	0.01 (−0.20, 0.24)	−0.48 (−1.19, 0.20)	−0.18 (−2.10, 1.73)	−0.66 (−2.59, 1.26)	0.11	−0.12 (−0.61, 0.21)	−1.03 (−2.33, 0.08)	−2.12 (−5.49, 1.26)	−3.15 (−6.53, 0.23)
	Kyn	0.05	−0.16	−0.01 (−0.33, 0.30)				−6.56	−0.31 (−1.06, 0.15)			
	HK	3.79^a^	−0.07^a^	**−0.27 (−0.69, −0.00)**				−0.06	−0.22 (−0.82, 0.22)			
	KA	−1.00	0.11^a^	−0.11 (−0.51, 0.21)				0.14	−0.14 (−0.73, 0.29)			
	XA	−0.67	0.19	−0.13 (−0.52, 0.11)				0.38	−0.26 (−0.98, 0.17)			
	AA	−0.51	0.01	−0.00 (−0.14, 0.12)				0.07	−0.04 (−0.33, 0.19)			
	HAA	−1.10	−0.00	0.00 (−0.20, 0.20)				−0.02	0.02 (−0.31, 0.39)			
	Pic	−0.68	−0.03	0.02 (−0.11, 0.19)				−0.06	0.04 (−0.18, 0.36)			
	QA	−2.31	−0.00	0.00 (−0.13, 0.16)				0.00	−0.01 (−0.28, 0.25)			
Total protein *(per SD = 69 kcal/day)*	Trp	0.43	−0.01	−0.00 (−0.15, 0.13)	0.36 (−0.25, 0.97)	−0.97 (−2.68, 0.73)	−0.61 (−2.34, 1.11)	0.11	0.05 (−0.20, 0.41)	0.76 (−0.29, 1.86)	0.42 (−2.73, 3.58)	1.18 (−1.99, 4.35)
	Kyn	0.02	−0.18	−0.00 (−0.21, 0.20)				−6.58	−0.16 (−0.72, 0.20)			
	HK	3.38^a^	−0.07^a^	−0.24 (−0.63, 0.00)				−0.06	−0.20 (−0.75, 0.19)			
	KA	3.60^a^	0.11^a^	**0.39 (0.02, 0.92)**				0.14	0.51 (−0.08, 1.37)			
	XA	1.65^a^	0.19	0.31 (−0.19, 0.91)				0.38	0.63 (−0.23, 1.69)			
	AA	0.92^a^	0.01	0.01 (−0.18, 0.19)				0.07	0.07 (−0.27, 0.45)			
	HAA	2.47^a^	−0.00	−0.00 (−0.34, 0.33)				−0.02	−0.05 (−0.66, 0.53)			
	Pic	2.46^a^	−0.03	−0.07 (−0.38, 0.20)				−0.06	−0.16 (−0.72, 0.31)			
	QA	18.13	−0.00	−0.02 (−0.21, 0.12)				0.00	0.07 (−0.16, 0.42)			
Animal−based protein *(per SD = 55 kcal/day)*	Trp	0.30	−0.01	−0.00 (−0.11, 0.10)	0.28 (−0.20, 0.77)	−0.76 (−2.12, 0.61)	−0.48 (−1.86, 0.91)	0.11	0.03 (−0.17, 0.31)	0.60 (−0.24, 1.48)	0.45 (−2.08, 2.97)	1.05 (−1.49, 3.58)
	Kyn	0.02	−0.16	−0.00 (−0.16, 0.15)				−6.56	−0.12 (−0.55, 0.17)			
	HK	2.59^a^	−0.07^a^	−0.19 (‐0.49, 0.01)				−0.06	−0.16 (−0.58, 0.15)			
	KA	2.70^a^	0.11^a^	**0.28 (0.01, 0.70)**				0.14	0.38 (−0.08, 1.04)			
	XA	1.35^a^	0.19	0.26 (−0.15, 0.77)				0.38	0.52 (−0.18, 1.38)			
	AA	0.75^a^	0.01	0.00 (−0.15, 0.15)				0.07	0.05 (−0.22, 0.37)			
	HAA	1.95^a^	−0.00	−0.00 (−0.27, 0.26)				−0.02	−0.04 (−0.52, 0.42)			
	Pic	2.04^a^	−0.03	−0.06 (−0.31, 0.17)				−0.06	−0.13 (−0.57, 0.25)			
	QA	15.22	−0.00	−0.02 (−0.17, 0.10)				0.00	0.06 (−0.13, 0.35)			
Plant−based protein *(per SD = 33 kcal/day)*	Trp	0.25	−0.01	−0.00 (−0.17, 0.16)	0.21 (−0.52, 0.95)	−0.01 (−2.26, 2.23)	0.20 (−2.09, 2.49)	0.11	0.03 (−0.32, 0.44)	0.29 (−0.96, 1.58)	0.42 (−3.69, 4.54)	0.71 (−3.47, 4.90)
	Kyn	0.01	−0.16	−0.00 (−0.20, 0.20)				−6.56	−0.06 (−0.67, 0.46)			
	HK	1.41	−0.07^a^	−0.10 (−0.48, 0.21)				−0.06	−0.08 (−0.56, 0.23)			
	KA	2.60	0.11^a^	0.27 (−0.08, 0.82)				0.14	0.36 (−0.16, 1.23)			
	XA	0.22	0.19	0.04 (−0.26, 0.41)				0.38	0.08 (−0.49, 0.76)			
	AA	0.33	0.01	0.00 (−0.13, 0.13)				0.07	0.02 (−0.21, 0.31)			
	HAA	0.34	−0.00	−0.00 (−0.17, 0.17)				−0.02	−0.01 (−0.32, 0.27)			
	Pic	0.39	−0.03	−0.01 (−0.19, 0.14)				−0.06	−0.03 (−0.36, 0.24)			
	QA	−7.66	−0.00	0.01 (−0.14, 0.19)				0.00	−0.03 (−0.38, 0.26)			
Total fat *(per SD = 207 kcal/day)*	Trp	1.71^a^	−0.01	−0.01 (−0.31, 0.26)	0.06 (−0.51, 0.62)	0.34 (−1.38, 2.05)	0.40 (−1.34, 2.14)	0.11	0.18 (−0.27, 0.79)	0.11 (−0.86, 1.10)	1.45 (−1.70, 4.61)	1.56 (−1.65, 4.77)
	Kyn	0.02	−0.18	−0.00 (−0.20, 0.19)				−6.58	−0.15 (−0.69, 0.22)			
	HK	−1.77	−0.07^a^	0.13 (−0.09, 0.44)				−0.06	0.11 (−0.15, 0.51)			
	KA	−0.62	0.11^a^	−0.07 (−0.42, 0.24)				0.14	−0.09 (−0.60, 0.33)			
	XA	0.11	0.19	0.02 (−0.21, 0.29)				0.38	0.04 (−0.40, 0.55)			
	AA	0.33	0.01	0.00 (−0.10, 0.11)				0.07	0.02 (−0.17, 0.27)			
	HAA	0.30	−0.00	−0.00 (−0.13, 0.13)				−0.02	−0.01 (−0.25, 0.21)			
	Pic	0.19	−0.03	−0.01 (−0.14, 0.11)				−0.06	−0.01 (−0.25, 0.20)			
	QA	−0.24	−0.00	0.00 (−0.12, 0.12)				0.00	−0.00 (−0.24, 0.24)			
Saturated fat *(per SD = 90 kcal/day)*	Trp	1.50	−0.01	−0.01 (−0.28, 0.24)	−0.11 (−0.71, 0.45)	0.28 (−1.48, 2.04)	0.16 (−1.62, 1.95)	0.11	0.16 (−0.24, 0.73)	−0.13 (−1.17, 0.87)	0.27 (−2.90, 3.44)	0.14 (−3.08, 3.36)
	Kyn	0.05^a^	−0.20	−0.01 (−0.34, 0.31)				−6.48	−0.33 (−1.07, 0.14)			
	HK	0.77	−0.07^a^	−0.06 (−0.34, 0.19)				−0.06	−0.05 (−0.39, 0.21)			
	KA	−0.49	0.11^a^	−0.05 (−0.41, 0.26)				0.14	−0.07 (−0.59, 0.36)			
	XA	0.24	0.19	0.05 (−0.18, 0.33)				0.39	0.09 (−0.34, 0.64)			
	AA	0.33	0.01	0.00 (−0.10, 0.11)				0.07	0.02 (−0.17, 0.26)			
	HAA	0.34	0.00	0.00 (−0.14, 0.13)				−0.02	−0.01 (−0.25, 0.22)			
	Pic	0.30	−0.03	−0.01 (−0.15, 0.11)				−0.06	−0.02 (−0.27, 0.19)			
	QA	16.49	−0.00	−0.02 (−0.21, 0.12)				0.00	0.06 (−0.15, 0.41)			
Unsaturated fat *(per SD = 136 kcal/day)*	Trp	0.32	−0.01	−0.00 (−0.16, 0.15)	0.18 (−0.46, 0.83)	0.06 (−1.92, 2.03)	0.24 (−1.78, 2.26)	0.11	0.03 (−0.27, 0.43)	0.25 (−0.85, 1.38)	1.52 (−2.19, 5.23)	1.76 (−2.02, 5.54)
	Kyn	−0.03	−0.20	0.01 (−0.22, 0.24)				−6.48	0.18 (−0.23, 0.82)			
	HK	−2.81	−0.07^a^	0.20 (−0.06, 0.60)				−0.06	0.17 (−0.17, 0.71)			
	KA	−0.17	0.11^a^	−0.02 (−0.41, 0.36)				0.14	−0.02 (−0.57, 0.50)			
	XA	−0.14	0.19	−0.03 (−0.35, 0.24)				0.39	−0.06 (−0.66, 0.46)			
	AA	0.01	0.01	−0.00 (−0.10, 0.10)				0.07	0.00 (−0.22, 0.23)			
	HAA	0.04	0.00	0.00 (−0.14, 0.14)				−0.02	−0.00 (−0.26, 0.25)			
	Pic	−0.14	−0.03	0.00 (−0.13, 0.15)				−0.06	0.01 (−0.24, 0.28)			
	QA	−14.91	−0.00	0.01 (−0.13, 0.21)				0.00	−0.06 (−0.43, 0.18)			
Alcohol *(per SD = 131 kcal/day)*	Trp	0.97	−0.01	−0.01 (−0.19, 0.17)	**0.64 (0.11, 1.21)**	1.45 (−0.18, 3.08)	**2.09 (0.45, 3.72)**	0.11	0.10 (−0.17, 0.51)	**1.02 (0.10, 2.05)**	1.42 (−1.18, 4.02)	2.44 (−0.16, 5.03)
	Kyn	−0.00	−0.18	0.00 (−0.13, 0.13)				−6.58	0.02 (−0.34, 0.43)			
	HK	0.03	−0.07^a^	−0.00 (−0.22, 0.22)				−0.06	−0.00 (−0.25, 0.24)			
	KA	3.56^a^	0.11^a^	**0.38 (0.04, 0.88)**				0.14	0.50 (−0.08, 1.29)			
	XA	1.46^a^	0.19	0.28 (−0.17, 0.80)				0.38	0.56 (−0.20, 1.49)			
	AA	0.10	0.01	0.00 (−0.07, 0.07)				0.07	0.00 (−0.13, 0.17)			
	HAA	−0.04	−0.00	−0.00 (−0.11, 0.10)				−0.02	0.00 (−0.19, 0.19)			
	Pic	1.43	−0.03	−0.04 (−0.25, 0.12)				−0.06	−0.09 (−0.46, 0.19)			
	QA	−22.20	−0.00	0.03 (−0.13, 0.23)				0.00	−0.09 (−0.46, 0.16)			
Fiber *(per SD = 12 kcal/day)*	Trp	0.97	−0.01	−0.01 (−0.20, 0.17)	0.08 (−0.46, 0.60)	**2.20 (0.62, 3.78)**	**2.28 (0.68, 3.88)**	0.11	0.11 (−0.17, 0.52)	0.21 (−0.68, 1.19)	**4.06 (1.25, 6.88)**	**4.27 (1.43, 7.12)**
	Kyn	−0.03	−0.18	0.00 (−0.21, 0.23)				−6.58	0.18 (−0.14, 0.73)			
	HK	−0.63	−0.07^a^	0.05 (−0.17, 0.30)				−0.06	0.04 (−0.19, 0.34)			
	KA	0.18	0.11^a^	0.02 (−0.26, 0.32)				0.14	0.02 (−0.37, 0.46)			
	XA	−0.05	0.19	−0.01 (−0.24, 0.20)				0.38	−0.02 (−0.46, 0.39)			
	AA	−0.74^a^	0.01	−0.00 (−0.16, 0.15)				0.07	−0.05 (−0.38, 0.22)			
	HAA	−0.07	−0.00	−0.00 (−0.11, 0.11)				−0.02	0.00 (−0.19, 0.20)			
	Pic	−0.23	−0.03	0.01 (−0.10, 0.13)				−0.06	0.01 (−0.17, 0.23)			
	QA	−21.61	−0.00	0.03 (−0.13, 0.22)				0.00	−0.08 (−0.45, 0.15)			
Micronutrient intake												
Vitamin B2 *(per SD = 0.4 mg/day)*	Trp	0.43	0.01	0.01 (−0.10, 0.13)	0.25 (−0.21, 0.73)	−0.22 (−1.63, 1.19)	0.03 (−1.41, 1.46)	0.17	0.07 (−0.17, 0.43)	0.54 (−0.32, 1.44)	−0.67 (−3.20, 1.86)	−0.13 (−2.72, 2.45)
	Kyn	0.01	−0.86	−0.01 (−0.16, 0.12)				−9.25^a^	−0.10 (−0.59, 0.30)			
	HK	1.02	−0.07^a^	−0.07 (−0.30, 0.11)				−0.05	−0.05 (−0.33, 0.13)			
	KA	2.13^a^	0.12^a^	0.25 (−0.00, 0.64)				0.17^a^	0.36 (−0.04, 0.99)			
	XA	0.79^a^	0.16	0.13 (−0.12, 0.47)				0.35	0.27 (−0.15, 0.89)			
	AA	0.17	−0.05	−0.00 (−0.08, 0.07)				0.07	0.01 (−0.13, 0.18)			
	HAA	1.98^a^	−0.00	−0.00 (−0.28, 0.27)				−0.02	−0.03 (−0.53, 0.44)			
	Pic	1.09	−0.03	−0.03 (−0.21, 0.10)				−0.05	−0.06 (−0.36, 0.17)			
	QA	13.83	−0.00	−0.02 (−0.17, 0.10)				0.00	0.07 (−0.13, 0.36)			
Vitamin B6 *(per SD = 0.6 mg/day)*	Trp	−0.10	0.01	−0.00 (−0.10, 0.09)	0.19 (−0.23, 0.62)	−0.51 (−1.78, 0.77)	−0.32 (−1.61, 0.98)	0.18	−0.02 (−0.30, 0.25)	0.34 (−0.47, 1.15)	0.94 (−1.42, 3.30)	1.28 (−1.13, 3.69)
	Kyn	0.00	−0.91	−0.00 (−0.12, 0.11)				−9.10^a^	−0.02 (−0.42, 0.37)			
	HK	1.10	−0.07^a^	−0.07 (−0.29, 0.08)				−0.05	−0.05 (−0.32, 0.12)			
	KA	1.65	0.12^a^	0.19 (−0.03, 0.54)				0.16^a^	0.27 (−0.06, 0.82)			
	XA	0.76^a^	0.16	0.12 (−0.12, 0.45)				0.34	0.26 (−0.14, 0.85)			
	AA	0.01	−0.00	−0.00 (−0.07, 0.06)				0.08	0.00 (−0.14, 0.14)			
	HAA	1.39^a^	0.00	0.00 (−0.21, 0.20)				−0.03	−0.04 (−0.42, 0.30)			
	Pic	1.46^a^	−0.03	−0.04 (−0.25, 0.12)				−0.06	−0.09 (−0.45, 0.20)			
	QA	3.11	−0.00	−0.00 (−0.10, 0.09)				0.00	0.01 (−0.16, 0.22)			
Magnesium *(per SD = 84.0 mg/day)*	Trp	0.38	0.01	0.00 (−0.14, 0.16)	0.38 (−0.32, 1.08)	1.07 (−0.96, 3.11)	1.45 (−0.60, 3.50)	0.16	0.06 (−0.27, 0.51)	0.85 (−0.36, 2.19)	2.46 (−1.07, 6.00)	3.31 (−0.26, 6.88)
	Kyn	−0.04	−0.42	0.02 (−0.26, 0.33)				−8.16	0.35 (−0.12, 1.15)			
	HK	0.07	−0.07^a^	−0.00 (−0.29, 0.28)				−0.05	−0.00 (−0.30, 0.28)			
	KA	2.68	0.11^a^	0.29 (−0.04, 0.81)				0.15	0.40 (−0.10, 1.22)			
	XA	0.55	0.17	0.10 (−0.14, 0.47)				0.38	0.21 (−0.25, 0.93)			
	AA	−0.10	−0.01	0.00 (−0.10, 0.10)				0.08	−0.01 (−0.22, 0.19)			
	HAA	1.54	−0.01	−0.01 (−0.27, 0.23)				−0.03	−0.05 (−0.54, 0.35)			
	Pic	1.11	−0.03	−0.04 (−0.25, 0.12)				−0.06	−0.06 (−0.44, 0.20)			
	QA	−12.10	−0.00	0.02 (−0.13, 0.21)				0.00	−0.05 (−0.43, 0.22)			
Zinc *(per SD = 2.4 mg/day)*	Trp	0.11	0.01	0.00 (−0.11, 0.12)	0.34 (−0.23, 0.92)	−1.17(−2.76, 0.42)	−0.83 (−2.44, 0.78)	0.17	0.02 (−0.30, 0.38)	0.76(−0.26, 1.88)	2.51 (−0.48, 5.51)	**3.28 (0.26, 6.29)**
	Kyn	0.00	−1.11	−0.00 (−0.15, 0.14)				−8.53	−0.02 (−0.49, 0.46)			
	HK	3.73^a^	−0.06^a^	−0.24 (−0.60, 0.01)				−0.05	−0.20 (−0.75, 0.24)			
	KA	3.41^a^	0.12^a^	**0.40 (0.04, 0.93)**				0.16	0.54 (−0.04, 1.38)			
	XA	1.45^a^	0.17	0.25 (−0.20, 0.78)				0.33	0.48 (−0.28, 1.42)			
	AA	0.59	0.00	0.00 (−0.13, 0.14)				0.07	0.04 (−0.20, 0.33)			
	HAA	2.06^a^	0.00	0.00 (−0.29, 0.29)				−0.02	−0.05 (−0.59, 0.43)			
	Pic	1.82^a^	−0.03	−0.06 (−0.31, 0.14)				−0.06	−0.11 (−0.55, 0.24)			
	QA	11.84	−0.00	−0.01 (−0.16, 0.11)				0.00	0.05 (−0.15, 0.36)			
Dietary patterns												
DHD score *(per SD = 15 points)*	Trp	0.03	0.01	0.00 (−0.08, 0.09)	−0.02 (−0.44, 0.41)	**2.45 (1.24, 3.66)**	**2.43 (1.20, 3.67)**	0.17	0.01 (−0.23, 0.26)	0.02 (−0.76, 0.84)	**2.68 (0.48, 4.88)**	**2.70 (0.45, 4.94)**
	Kyn	−0.03	−0.04	0.00 (−0.19, 0.19)				−8.22	0.24 (−0.06, 0.74)			
	HK	−1.89	−0.07^a^	0.13 (−0.02, 0.36)				−0.04	0.08 (−0.14, 0.40)			
	KA	−0.50	0.11^a^	−0.05 (−0.30, 0.16)				0.16	−0.08 (−0.46, 0.25)			
	XA	−0.47	0.22	−0.11 (−0.39, 0.06)				0.44	−0.21 (−0.73, 0.10)			
	AA	−0.25	−0.01	0.00 (−0.07, 0.08)				0.08	−0.02 (−0.19, 0.11)			
	HAA	0.20	−0.03	−0.01 (−0.11, 0.08)				−0.06	−0.01 (−0.20, 0.15)			
	Pic	−0.57	−0.02	0.01 (−0.08, 0.13)				−0.05	0.03 (−0.13, 0.24)			
	QA	−4.81	−0.00	0.01 (−0.08, 0.12)				0.00	−0.02 (−0.22, 0.13)			
WCRF/AICR *(per SD = 0.7 points)*	Trp	0.05	0.02	0.00 (−0.08, 0.08)	0.07 (−0.30, 0.45)	−0.20 (−1.34, 0.94)	−0.12 (−1.29, 1.05)	0.17	0.01 (−0.23, 0.26)	0.09 (−0.59, 0.80)	0.95 (−1.14, 3.04)	1.03 (−1.11, 3.18)
	Kyn	−0.01	−1.06	0.01 (−0.09, 0.14)				−9.05	0.09 (−0.22, 0.49)			
	HK	−0.12	−0.07^a^	0.01 (−0.15, 0.17)				−0.05	0.01 (−0.16, 0.18)			
	KA	0.40	0.12^a^	0.05 (−0.18, 0.30)				0.16^a^	0.06 (−0.25, 0.44)			
	XA	−0.08	0.17	−0.01 (−0.19, 0.13)				0.38	−0.03 (−0.38, 0.27)			
	AA	−0.24	−0.00	0.00 (−0.07, 0.07)				0.08	−0.02 (−0.19, 0.11)			
	HAA	−0.16	−0.01	0.00 (−0.08, 0.09)				−0.03	0.00 (−0.15, 0.17)			
	Pic	−0.25	−0.03	0.01 (−0.07, 0.10)				−0.05	0.01 (−0.12, 0.18)			
	QA	−10.45	−0.00	0.01 (−0.08, 0.13)				0.00	−0.05 (−0.28, 0.11)			

*Note*: Models with macronutrients as exposure are adjusted for age, sex (male, female), renal function (μmol/L), weeks since end treatment (weeks), chemotherapy (yes, no), comorbidities (0, 1, ≥2), stoma (yes, no), educational level (low, medium, high), BMI (kg/m^2^), MVPA (h/week), smoking status (never, former, current), prolonged sedentary time (h/day), and energy intake using the all‐components method (kcal/day). Models with micronutrients as exposure are adjusted for age, sex (male, female), renal function (μmol/L), weeks since end treatment (weeks), chemotherapy (yes, no), comorbidities (0, 1, ≥2), stoma (yes, no), educational level (low, medium, high), BMI (kg/m^2^), MVPA (h/week), smoking status (never, former, current), prolonged sedentary time (h/day), alcohol intake (kcal/day), and total energy intake (kcal/day). Models with dietary pattern scores as exposure are adjusted for age, sex (male, female), renal function (μmol/L), weeks since end treatment (weeks), chemotherapy (yes, no), comorbidities (0, 1, ≥2), stoma (yes, no), educational level (low, medium, high), BMI (kg/m^2^), MVPA (h/week), smoking status (never, former, current), prolonged sedentary time (h/day), and total energy intake (kcal/day). Bold indicates a statistically significant mediator‐specific interventional indirect effect, total indirect effect, direct effect or total effect (*p* <.05).

Abbreviations: AA, anthranilic acid; DHD, Dutch Healthy Diet; HAA, 3‐hydroxyanthranilic acid; HK, 3‐hydroxykynurenine; KA, kynurenic acid; Kyn, kynurenine; Pic, picolinic acid; QA, quinolinic acid; SD, standard deviation; Trp, tryptophan; WCRF/AICR, World Cancer Research Fund/American Institute for Cancer Research; XA, xanthurenic acid.

^a^ Indicates a statistically significant *a*‐ or *b*‐path (*p* <.05).

The total indirect effect of the whole KP was only significant on the longitudinal association between alcohol intake and better physical functioning (total indirect effect: 0.64; 0.11, 1.21) (Table 3). In addition, we observed that plasma KA statistically significantly mediated the association of total protein (indirect effect: 0.39; 0.02, 0.92), animal‐based protein (indirect effect: 0.28; 0.01, 0.70), alcohol (indirect effect: 0.38; 0.04, 0.88), and zinc (indirect effect: 0.40; 0.04, 0.93) with better physical functioning. Furthermore, plasma HK mediated the inverse association of polysaccharides with physical functioning (indirect effect: −0.27; −0.69, −0.00) (Table 3).

Lastly, we observed statistically significant associations for both the *a*‐ and *b*‐paths of the mediation models with total protein, animal‐based protein, and zinc, plasma HK, and physical functioning, and mediation models with total carbohydrates, mono‐ and disaccharides, and vitamin B2, plasma KA, and physical functioning. However, the mediator‐specific interventional indirect effects were not statistically significant (total protein: −0.24; −0.63, 0.00 and animal‐based protein: −0.19; −0.49, 0.01 and zinc: −0.24; −0.60, 0.01 and total carbohydrates: −0.31; −0.83, 0.01 and mono‐ and disaccharides: −0.24; −0.62, 0.01 and vitamin B2: 0.25; −0.00, 0.64) (Table 3).

### 3.3 Multilevel multiple‐parallel mediator analysis with diet, the KP, and role functioning

Similarly to physical functioning, we found that better adherence to the DHD recommendations (total effect: 2.70; 0.45, 4.94), as well as higher intake of fiber (total effect: 4.27; 1.43, 7.12) and zinc (total effect: 3.28; 0.26, 6.29) was statistically significantly associated with better role functioning (Table 3). Additionally, higher total carbohydrate intake (total effect: −4.39; −7.73, −1.06) was associated with worse role functioning (Table 3). Direct effect estimates adjusted for all kynurenines remained statistically significant for associations of fiber (direct effect: 4.06; 1.25, 6.88) and the DHD score (direct effect: 2.68; 0.48, 4.88) with better role functioning (Table 3). None of the total effect estimates and direct effect estimates of intake of any other nutrient and the WCRF dietary score on role functioning were statistically significant (Table 3).

Considering all metabolites simultaneously, the KP with all currently available metabolites was a significant mediator in the longitudinal association between higher carbohydrate intake and poorer role functioning (total indirect effect: −1.27; −2.58, −0.13) and between higher alcohol intake and better role functioning (total indirect effect: 1.02; 0.10, 2.05) (Table 3). None of the mediator‐specific interventional indirect effects were statistically significant. However, we observed a statistically significant *a*‐ and *b*‐path for vitamin B2 intake, plasma KA, and role functioning, but the mediator‐specific interventional indirect effect was not statistically significant (indirect effect: 0.36; −0.04, 0.99) (Table 3).

### 3.4 Multilevel single mediator analysis with diet, KP ratios, and physical and role functioning

Total and direct effect estimates of associations of dietary intake with physical and role functioning in single mediator models with KP ratios as mediator were similar as for parallel‐multiple mediator models (Table 4). No statistically significant indirect effect estimates were observed for KTR as mediator with physical and role functioning. In contrast, the KA/QA ratio was a significant mediator in the association of total carbohydrates (−0.94; −1.77, −0.30) and mono‐ and disaccharides (−0.64; −1.24, −0.18) with worse role functioning. Additionally, the KA/QA ratio mediated associations of higher protein (0.57; 0.08, 1.23), animal‐based protein (0.41; 0.03, 0.92), plant‐based protein (0.63; 0.02, 1.45), alcohol (0.79, 0.26, 1.49), magnesium (0.86; 0.22, 1.70), and zinc (0.63; 0.13, 1.30) intake with better role functioning. Moreover, HKr was found to be a significant mediator in associations of higher carbohydrate (−0.53; −1.20, −0.07) and polysaccharide (−0.85; −1.69, −.021) intake with worse role functioning, and of higher intake of fat (0.42; 0.01, 1.03) and higher adherence to DHD recommendations (0.37; 0.04, 4.48) with better role functioning. Indirect effect estimates for associations with physical functioning as outcome were similar in direction and significance to those for role functioning as outcome but less pronounced.

**TABLE 4:** Analysis of the kynurenine‐to‐tryptophan ratio (KTR), kynurenic acid‐to‐quinolinic acid ratio (KA/QA), and hydroxykynurenine ratio (HKr) as mediator in the longitudinal association of dietary intake with physical functioning and role functioning as outcome; single mediator model.

			Physical functioning (0–100)				Role functioning (0–100)			
Exposure	Media‐tor	*a*‐path	*b*‐path	Indirect effect (a*b)	Direct effect	Total effect	b‐path	Indirect effect (a*b)	Direct effect	Total effect
Macronutrient intake										
Total carbohydrates *(per SD = 230 kcal/day)*	KTR	2.27^a^	−0.08	−0.19 (−0.60, 0.16)	−0.57 (−2.48, 1.34)	−0.76 (−2.64, 1.12)	−0.14	−0.32 (−1.03, 0.31)	−**4.15** **(**−**7.49**, −**0.81)**	−**4.46** **(**−**7.75**, −**1.17)**
	KA/QA	−0.01^a^	62.99^a^	−**0.70 (**−**1.23**, −**0.28)**	−0.12 (−1.98, 1.75)	−0.81 (−2.69, 1.06)	84.40^a^	−**0.94 (**−**1.77**, −**0.30)**	−**3.46** **(**−**6.76**, −**0.15)**	−**4.39** **(**−**7.68**, −**1.11)**
	HKr	0.03^a^	−12.70^a^	−**0.39 (**−**0.82**, −**0.07)**	−0.35 (−2.23, 1.52)	−0.75 (−2.63, 1.14)	−17.36^a^	−**0.53 (**−**1.20**, −**0.07)**	−**3.96** **(**−**7.23**, −**0.69)**	−**4.50** **(**−**7.77**, −**1.22)**
Mono‐ and disaccharides *(per SD = 137 kcal/day)*	KTR	1.40^a^	−0.08	−0.12 (−0.39, 0.10)	−0.26 (−1.72, 1.20)	−0.38 (−1.83, 1.06)	−0.14	−0.19 (−0.66, 0.19)	−2.20 (−4.75, 0.36)	−2.39 (−4.92, 0.14)
	KA/QA	−0.01^a^	63.27^a^	−**0.48 (**−**0.87**, −**0.17)**	0.10 (−1.33, 1.53)	−0.38 (−1.82, 1.07)	84.42^a^	−**0.64 (**−**1.24**, −**0.18)**	−1.77 (−4.29, 0.76)	−2.40 (−4.92, 0.12)
	HKr	−0.00	−12.78^a^	0.00 (−0.25, 0.25)	−0.31 (−1.74, 1.12)	−0.31 (−1.76, 1.34)	−17.42^a^	0.00 (−0.35, 0.36)	−2.42 (−4.92, 0.08)	−2.42 (−4.94, 0.10)
Polysaccharides *(per SD = 140 kcal/day)*	KTR	1.29^a^	−0.08	−0.11 (−0.39, 0.09)	−0.46 (−2.38, 1.45)	−0.57 (−2.48, 1.34)	−0.14	−0.18 (−0.67, 0.18)	−3.00 (−6.36, 0.35)	−3.18 (−6.52, 0.15)
	KA/QA	−0.01	63.27^a^	−0.33 (−0.78, 0.05)	−0.34 (−2.21, 1.53)	−0.67 (−2.58, 1.24)	84.42^a^	−0.44 (−1.09, 0.06)	−2.61 (−5.93, 0.70)	−3.05 (−6.38, 0.28)
	HKr	0.05^a^	−12.78^a^	−**0.63 (**−**1.13**, −**0.23)**	−0.04 (−1.95, 1.87)	−0.67 (−2.58, 1.25)	−17.42^a^	−**0.85 (**−**1.69**, −**0.21)**	−2.32 (−5.68, 1.03)	−3.18 (−6.50, 0.15)
Total protein *(per SD = 69 kcal/day)*	KTR	0.41	−0.08	−0.03 (−0.20, 0.07)	−0.58 (−2.29, 1.13)	−0.61 (−2.32, 1.10)	−0.14	−0.06 (−0.35, 0.13)	1.25 (−1.87, 4.38)	1.20 (−1.93, 4.32)
	KA/QA	0.01^a^	62.99^a^	**0.42 (0.07, 0.86)**	−1.02 (−2.71, 0.66)	−0.60 (−2.31, 1.11)	84.40^a^	**0.57 (0.08, 1.23)**	0.59 (−2.52, 3.71)	1.16 (−1.96, 4.29)
	HKr	0.00	−12.70^a^	−0.03 (−0.34, 0.27)	−0.55 (−2.25, 1.14)	−0.58 (−2.30, 1.13)	−17.36^a^	−0.04 (−0.49, 0.39)	1.18 (−1.92, 4.28)	1.14 (−1.99, 4.26)
Animal‐based protein *(per SD = 55 kcal/day)*	KTR	0.39	−0.08	−0.03 (−0.17, 0.05)	−0.45 (−1.82, 0.93)	−0.48 (−1.85, 0.89)	−0.14	−0.05 (−0.30, 0.10)	1.12 (−1.39, 3.62)	1.06 (−1.44, 3.57)
	KA/QA	0.00^a^	62.68^a^	**0.30 (0.03, 0.64)**	−0.76 (−2.11, 0.58)	−0.46 (−1.83, 0.91)	84.16^a^	**0.41 (0.03, 0.92)**	0.61 (−1.88, 3.10)	1.02 (−1.49, 3.52)
	HKr	0.00	−12.69^a^	−0.02 (−0.27, 0.22)	−0.43 (−1.78, 0.93)	−0.45 (−1.83, 0.93)	−17.34^a^	−0.03 (−0.39, 0.31)	1.04 (−1.44, 3.52)	1.01 (−1.49, 3.51)
Plant‐based protein *(per SD = 33 kcal/day)*	KTR	−0.48	−0.08	0.04 (−0.10, 0.25)	0.19 (−2.08, 2.45)	0.23 (−2.04, 2.49)	−0.14	0.07 (−0.18, 0.43)	0.66 (−3.47, 4.79)	0.72 (−3.41, 4.86)
	KA/QA	0.01^a^	62.68^a^	**0.47 (0.02, 1.02)**	−0.29 (−2.53, 1.94)	0.17 (−2.09, 2.44)	84.16^a^	**0.63 (0.02, 1.45)**	0.19 (−3.91, 4.29)	0.82 (−3.31, 4.95)
	HKr	−0.00	−12.69^a^	0.01 (−0.39, 0.42)	0.18 (−2.07, 2.43)	0.19 (−2.09, 2.47)	−17.34^a^	0.02 (−0.55, 0.60)	0.75 (−3.35, 4.85)	0.77 (−3.36, 4.90)
Total fat *(per SD = 207 kcal/day)*	KTR	−1.33^a^	−0.08	0.11 (−0.09, 0.38)	0.35 (−1.38, 2.09)	0.46 (−1.26, 2.18)	−0.14	0.18 (−0.18, 0.66)	1.37 (−1.81, 4.56)	1.56 (−1.61, 4.73)
	KA/QA	−0.00	62.99^a^	−0.14 (−0.52, 0.21)	0.60 (−1.08, 2.29)	0.47 (−1.25, 2.18)	84.40^a^	−0.19 (−0.73, 0.28)	1.76 (−1.37, 4.90)	1.58 (−1.59, 4.74)
	HKr	−0.02^a^	−12.70^a^	**0.31 (0.02, 0.70)**	0.05 (−1.67, 1.77)	0.36 (−1.37, 2.09)	−17.36^a^	**0.42 (0.01, 1.03)**	1.24 (−1.92, 4.39)	1.66 (−1.51, 4.82)
Saturated fat *(per SD = 90 kcal/day)*	KTR	−0.28	−0.08	0.02 (−0.09, 0.18)	0.02 (−1.75, 1.79)	0.04 (−1.73, 1.81)	−0.14	0.04 (−0.16, 0.30)	0.19 (−2.99, 3.36)	0.22 (−2.96, 3.40)
	KA/QA	−0.00	63.08^a^	−0.26 (−0.67, 0.09)	0.34 (−1.40, 2.08)	0.08 (−1.69, 1.85)	84.23^a^	0.35 (−0.94, 0.11)	0.54 (−2.61, 3.68)	0.19 (−2.99, 3.36)
	HKr	−0.00	−12.72^a^	0.06 (−0.25, 0.38)	−0.02 (−1.77, 1.73)	0.03 (−1.74, 1.81)	−17.33^a^	0.08 (−0.35, 0.55)	0.14 (−3.01, 3.29)	0.22 (−2.95, 3.39)
Unsaturated fat *(per SD = 136 kcal/day)*	KTR	−1.26^a^	−0.08	0.11 (−0.09, 0.39)	0.34 (−1.66, 2.35)	0.45 (−1.54, 2.45)	−0.14	0.17 (−0.18, 0.66)	1.50 (−2.25, 5.24)	1.67 (−2.06, 5.40)
	KA/QA	0.00	63.08^a^	0.09 (−0.33, 0.52)	0.33 (−1.62, 2.29)	0.42 (−1.57, 2.41)	84.23^a^	0.12 (−0.44, 0.72)	1.61 (−2.08, 5.30)	1.73 (−2.00, 5.46)
	HKr	−0.02	−12.72^a^	0.30 (−0.04, 0.74)	0.04 (−1.95, 2.03)	0.34 (−1.67, 2.35)	−17.33^a^	0.41 (−0.05, 1.09)	1.39 (−2.33, 5.10)	1.80 (−1.94, 5.53)
Alcohol *(per SD = 131 kcal/day)*	KTR	−0.73	−0.08	0.06 (−0.06, 0.24)	**2.00** **(0.37, 3.64)**	**2.06** **(0.43, 3.70)**	−0.14	0.10 (−0.11, 0.42)	2.37 (−0.20, 4.95)	2.47 (−0.10, 5.04)
	KA/QA	0.01^a^	62.99^a^	**0.59 (0.24, 1.04)**	1.51 (−0.12, 3.13)	**2.10** **(0.47, 3.73)**	84.40^a^	**0.79 (0.26, 1.49)**	1.63 (−0.94, 4.20)	2.42 (−0.14, 4.99)
	HKr	−0.01	−12.70^a^	0.16 (−0.08, 0.45)	**1.90** **(0.30, 3.51)**	**2.06** **(0.44, 3.68)**	−17.36^a^	0.21 (−0.11, 0.66)	2.19 (−0.34, 4.72)	2.40 (−0.14, 4.95)
Fiber *(per SD = 12 kcal/day)*	KTR	−1.02^a^	−0.08	0.09 (−0.07, 0.31)	**2.24** **(0.65, 3.83)**	**2.33** **(0.74, 3.91)**	−0.14	0.14 (−0.14, 0.53)	**4.15** **(1.33, 6.97)**	**4.29** **(1.48, 7.10)**
	KA/QA	0.00	62.99^a^	0.21 (−0.10, 0.57)	**2.10** **(0.55, 3.66)**	**2.31** **(0.73, 3.90)**	84.40^a^	0.28 (−0.13, 0.80)	**4.03** **(1.26, 6.81)**	**4.31** **(1.51, 7.12)**
	HKr	−0.01	−12.70^a^	0.13 (−0.13, 0.44)	**2.23** **(0.66, 3.80)**	**2.35** **(0.77, 3.94)**	−17.36^a^	0.18 (−0.18, 0.63)	**4.12** **(1.34, 6.91)**	**4.30** **(1.50, 7.10)**
Micronutrient intake										
Vitamin B2 *(per SD = 0.4 mg/day)*	KTR	0.28	−0.11	−0.03 (−0.18, 0.07)	0.09 (−1.33, 1.51)	0.06 (−1.36, 1.48)	−0.21	−0.06 (−0.34, 0.13)	−0.09 (−2.63, 2.45)	−0.15 (−2.69, 2.39)
	KA/QA	0.00	53.70^a^	0.21 (−0.08, 0.53)	−0.16 (−1.56, 1.23)	0.04 (−1.38, 1.46)	95.69^a^	0.31 (−0.11, 0.83)	−0.43 (−2.94, 2.08)	−0.12 (−2.66, 2.42)
	HKr	−0.01	−12.86^a^	0.09 (−0.15, 0.36)	−0.00 (−1.41, 1.41)	0.08 (−1.34, 1.51)	−19.32^a^	0.13 (−0.23, 0.56)	−0.35 (−2.86, 2.16)	−0.22 (−2.76, 2.32)
Vitamin B6 *(per SD = 0.6 mg/day)*	KTR	−0.01	−0.11	0.00 (−0.11, 0.11)	−0.21 (−1.50, 1.07)	−0.21 (−1.50, 1.07)	−0.21	0.00 (−0.21, 0.21)	1.25 (−1.12, 3.62)	1.25 (−1.12, 3.62)
	KA/QA	0.00	64.13^a^	0.21 (−0.06, 0.51)	−0.42 (−1.68, 0.84)	−0.21 (−1.49, 1.07)	93.79^a^	0.30 (−0.08, 0.78)	0.94 (−1.41, 3.28)	1.24 (−1.13, 3.61)
	HKr	−0.00	−12.89^a^	0.00 (−0.23, 0.24)	−0.27 (−1.54, 1.01)	−0.27 (−1.56, 1.03)	−19.21^a^	0.00 (−0.35, 0.37)	1.24 (−1.11, 3.60)	1.25 (−1.12, 3.62)
Magnesium *(per SD = 84 mg/day)*	KTR	−0.79	−0.10	0.08 (−0.06, 0.31)	1.42 (−0.61, 3.45)	1.50 (−0.53, 3.53)	−0.20	0.15 (−0.11, 0.58)	3.16 (−0.36, 6.68)	3.31 (−0.20, 6.83)
	KA/QA	0.01^a^	61.81^a^	**0.59 (0.17, 1.11)**	0.86 (−1.15, 2.87)	1.44 (−0.58, 3.47)	90.71^a^	**0.86 (0.22, 1.70)**	2.52 (−0.97, 6.01)	3.38 (−0.13, 6.89)
	HKr	−0.02	−12.77^a^	0.22 (−0.10, 0.62)	1.43 (−0.57, 3.44)	1.65 (−0.38, 3.69)	−18.42^a^	0.32 (−0.14, 0.95)	2.95 (−0.53, 6.43)	3.27 (−0.23, 6.78)
Zinc *(per SD = 2.4 mg/day)*	KTR	0.50	−0.10	−0.05 (−0.23, 0.06)	−0.78 (−2.37, 0.82)	−0.83 (−2.42, 0.77)	−0.22	−0.11 (−0.46, 0.11)	**3.44** **(0.47, 6.40)**	**3.33** **(0.36, 6.30)**
	KA/QA	0.01^a^	66.46^a^	**0.47 (0.12, 0.90)**	−1.30 (−2.87, 0.27)	−0.83 (−2.43, 0.76)	89.57^a^	**0.63 (0.13, 1.30)**	2.68 (−0.28, 5.63)	**3.31** **(0.34, 6.28)**
	HKr	0.01	−12.74^a^	−0.13 (−0.44, 0.15)	−0.68 (−2.27, 0.90)	−0.81 (−2.41, 0.80)	−19.36^a^	−0.19 (−0.69, 0.23)	**3.44** **(0.49, 6.38)**	**3.25** **(0.28, 6.22)**
Dietary patterns										
DHD score *(per SD = 15 points)*	KTR	−0.77^a^	−0.10	0.08 (−0.04, 0.25)	**2.38** **(1.15, 3.60)**	**2.45** **(1.23, 3.68)**	−0.21	0.16 (−0.05, 0.49)	**2.44** **(0.22, 4.65)**	**2.60** **(0.39, 4.81)**
	KA/QA	−0.00	67.03^a^	−0.01 (−0.23, 0.27)	**2.45** **(1.26, 3.65)**	**2.45** **(1.22, 3.67)**	100.37^a^	−0.01 (−0.43, 0.40)	**2.64** **(0.46, 4.81)**	**2.63** **(0.42, 4.84)**
	HKr	−0.02^a^	−12.30^a^	**0.24 (0.03, 0.51)**	**2.26** **(1.04, 3.47)**	**2.49** **(1.27, 3.72)**	−18.97^a^	**0.37 (0.04, 0.83)**	**2.28** **(0.08, 4.48)**	**2.65** **(0.44, 4.86)**
WCRF/AICR dietary score *(per SD = 0.7 points)*	KTR	−0.64	−0.12	0.08 (−0.02, 0.25)	−0.19 (−1.35, 0.98)	−0.11 (−1.27, 1.05)	−0.23	0.14 (−0.04, 0.45)	0.90 (−1.22, 3.01)	1.04 (−1.08, 3.16)
	KA/QA	0.00	67.03^a^	0.16 (−0.09, 0.45)	−0.27 (−1.41, 0.86)	−0.11 (−1.27, 1.05)	98.87^a^	0.24 (−0.13, 0.67)	0.81 (−1.27, 2.90)	1.06 (−1.06, 3.17)
	HKr	−0.01	−13.38^a^	0.13 (−0.07, 0.38)	−0.22 (−1.36, 0.93)	−0.08 (−1.25, 1.08)	−19.79^a^	0.20 (−0.10, 0.59)	0.86 (−1.24, 2.96)	1.06 (−1.06, 3.17)

*Note*: Models with macronutrients as exposure are adjusted for age, sex (male, female), renal function (μmol/L), weeks since end treatment (weeks), chemotherapy (yes, no), comorbidities (0, 1, ≥2), stoma (yes, no), educational level (low, medium, high), BMI (kg/m^2^), MVPA (h/week), smoking status (never, former, current), prolonged sedentary time (h/day), and energy intake using the all‐components method (kcal/day). Models with micronutrients as exposure are adjusted for age, sex (male, female), renal function (μmol/L), weeks since end treatment (weeks), chemotherapy (yes, no), comorbidities (0, 1, ≥2), stoma (yes, no), educational level (low, medium, high), BMI (kg/m^2^), MVPA (h/week), smoking status (never, former, current), prolonged sedentary time (h/day), alcohol intake (kcal/day), and total energy intake (kcal/day). Models with dietary pattern scores as exposure are adjusted for age, sex (male, female), renal function (μmol/L), weeks since end treatment (weeks), chemotherapy (yes, no), comorbidities (0, 1, ≥2), stoma (yes, no), educational level (low, medium, high), BMI (kg/m^2^), MVPA (h/week), smoking status (never, former, current), prolonged sedentary time (h/day), and total energy intake (kcal/day). Bold indicates a statistically significant mediator‐specific interventional indirect effect, total indirect effect, direct effect or total effect (*p* <.05).

Abbreviations: DHD, Dutch Healthy Diet; HWCRF/AICR, World Cancer Research Fund/American Institute for Cancer Research; Kr, hydroxykynurenine ratio (3‐hydroxykynurenine ratio: [kynurenic acid + xanthurenic acid + anthranilic acid +3‐hydroxyanthranilic acid]); KTR, kynurenine‐to‐tryptophan ratio; KA/QA, kynurenic acid‐to‐quinolinic acid; SD, standard deviation.

^a^ Indicates a statistically significant *a*‐ or *b*‐path (*p* <.05).

### 3.5 Secondary analyses

Parallel‐multiple mediator models with the subscale global QoL and the EORTC summary score as outcomes showed generally similar but weaker total and direct effect estimates compared to those for physical and role functioning (Supplementary Table 1). More specifically, statistically significant total and direct effects were observed for total carbohydrate and mono‐ and disaccharide intake with worse global QoL and lower EORTC summary score, plant‐based protein with better global QoL, and saturated fat intake with a higher EORTC summary score. Also, total effect estimates were statistically significant for higher polysaccharide intake with lower EORTC summary score and higher alcohol intake with higher EORTC summary score (Supplementary Table 1). Interestingly, plasma XA was found to be a significant mediator in the association between intakes of total carbohydrates, mono‐ and disaccharides, total protein, animal‐based protein, alcohol, vitamin B2, vitamin B6, and zinc with global QoL, and plasma HK significantly mediated in the association between polysaccharide and zinc intake with EORTC summary score (Supplementary Table 1).

Single mediation models with kynurenine ratios as mediators yielded similar results regarding direct and total effects in terms of direction, magnitude, and statistical significance compared to main analyses with physical and role functioning as outcome (Supplementary Tables 2–4). No statistically significant indirect effect estimates were observed for KTR as mediator with any outcome (Supplementary Table 2). In contrast, the KA/QA ratio was a significant mediator of the association of total carbohydrates, mono‐ and disaccharides, alcohol, magnesium, and zinc intake with global QoL and the EORTC summary score (Supplementary Table 3). The KA/QA ratio also mediated the association of intake of total protein, animal‐based protein, and plant‐based protein with the EORTC summary score (Supplementary Table 3). In addition, HKr was found to be a significant mediator for the association of polysaccharide intake and the DHD score with the EORTC summary score (Supplementary Table 4).

## 4 DISCUSSION

The current study revealed that all KP metabolites simultaneously only mediated longitudinal associations of total carbohydrate intake with worse role functioning and of alcohol intake with better physical and role functioning in the first year after CRC treatment. Evaluating the individual kynurenines, plasma HK, KA, and XA were found to be significant mediators in several associations between dietary intake and HRQoL outcomes. Moreover, the KA/QA ratio was a mediator in associations of total carbohydrate, mono‐ and disaccharides, total protein, animal‐based protein, plant‐based protein, alcohol, magnesium, and zinc with physical and role functioning, global QoL, and the EORTC summary score. Additionally, HKr mediated the association of polysaccharides and the DHD with physical and role functioning, and the EORTC summary score, as well as the association between total carbohydrates and total fat with physical and role functioning. Lastly, we observed that, in general, higher intake of carbohydrates was significantly associated with worse HRQoL outcomes, while better adherence to the DHD recommendations and higher intakes of plant‐based protein, fiber, saturated fat, alcohol, and zinc were significantly associated with better HRQoL outcomes up to 12 months post CRC treatment.

### 4.1 Total and direct effects of dietary intake on HRQoL outcomes

In CRC survivors, we observed that higher carbohydrate intake, including total carbohydrates, mono‐ and disaccharides, and polysaccharides, was longitudinally associated with poorer role functioning, global QoL, and overall HRQoL after CRC treatment. This finding contrasts with a cross‐sectional study in patients treated for malignant neoplasm, including patients with lower digestive tract cancers, which did not find a significant association between carbohydrate intake, assessed with a Food Frequency Questionnaire, and HRQoL assessed by the EORTC QLQ‐C30.^41^ While studies specifically investigating carbohydrate intake in relation to quality of life after cancer remain limited, several studies have examined the impact of a ketogenic diet, characterized by low carbohydrate and high fat content, on such outcomes.^42^ A randomized controlled trial in patients receiving chemotherapy for stage II or III breast, prostate, CRC, lung, or cervical cancer found that those adhering to a 16‐week ketogenic diet (10% carbohydrates, 15% proteins, 75% fats) reported higher overall quality of life evaluated using the EORTC QLQ‐C30 compared to those following a standard traditional diet.^43^ Additionally, an online survey among 96 cancer patients, including a subset of patients with CRC, demonstrated that adhering to a ketogenic diet or low‐carbohydrate diet improved quality of life in more than two‐thirds of study participants.^44^ Similar, we observed that lower carbohydrate intake and higher fat intake, consistent with the ketogenic diet, were associated with better HRQoL outcomes in CRC survivors. Furthermore, in line with results of the current study, previous research among CRC survivors has shown a positive association between fiber intake and better HRQoL^45^ and improved physical and role functioning.^10^ In our study, significant associations were observed for fiber intake with physical and role functioning. Remarkably, we observed that higher alcohol intake was longitudinally associated with better physical functioning and overall HRQoL in the current study, which can possibly be explained by potential reverse causality – participants experiencing improvements in their quality of life might have a higher likelihood of alcohol consumption compared to those who have not yet experienced better quality of life.^9^ Regarding protein intake, a previous randomized controlled trial focused on increasing protein intake among CRC survivors demonstrated better global QoL and better physical and role functioning compared to those who received routine care for 6 months.^46^ We also observed a significant positive association with global QoL, but only with intakes of plant‐based protein. Moreover, consistent with cross‐sectional analysis of the 2006 DHD*i*, including nine dietary components and one physical activity component, and HRQoL within the EnCoRe cohort,^8^ we found that higher adherence to the 2015 DHD recommendations, including dietary components only, was longitudinally associated with higher scores on physical functioning and role functioning. To the best of our knowledge, we are the first to report a positive association between intake of saturated fat and a higher EORTC summary score. Finally, we observed that a higher zinc intake was associated with better role functioning, but not with other aspects of quality of life. This finding is in line with another randomized controlled trial among CRC patients, which concluded that oral zinc supplementation (35 mg/day) 45 days before and up to 12 weeks post‐chemotherapy treatment can preserve quality of life.^47^

### 4.2 Longitudinal associations of dietary intake with the KP (*a*‐path) and the KP with HRQoL outcomes (*b*‐path)

We have previously described and discussed longitudinal associations between macronutrient and micronutrient intake, as well as adherence to the DHD and dietary WCRF recommendations, with plasma kynurenines and ratios (*a*‐path),^12^, ^14^ and between plasma kynurenines and ratios and HRQoL outcomes.^19^ Any small differences between the estimates and significance level in the current study and previous analyses are likely explained by slight differences in sample size and the selected confounders that were adjusted for. More specifically, we estimated parallel‐multiple mediator models in which all metabolites were included simultaneously to allow estimation of a total indirect effect. This means that KP metabolite‐HRQoL associations were adjusted for all other KP metabolites, in contrast to previous analyses.

### 4.3 The potential mediating role of the KP

In the current analyses when using multiple‐parallel mediator models including all currently available metabolites simultaneously, most metabolites of the KP were not identified as mediators in the association between dietary intake and HRQoL outcomes in CRC survivors, contradicting our initial hypothesis. Only HK, KA, and/or XA were significant mediators in certain nutrient–HRQoL outcome associations. This could be attributed to the simultaneous inclusion of all KP metabolites with diverse and often opposing biological functions (e.g., neurotoxic and neuroprotective) in a parallel‐multiple mediator model, thereby attenuating mediating effects through certain KP metabolites. However, despite the different functions of KP metabolites, it is relevant to consider them all in a single model, as they coexist in the body and naturally interact with one another.

Because the balance of metabolites within the KP are commonly summarized using the KTR,^25^ HKr,^26^ and KA/QA^27^ ratios, we also performed mediation analyses using these ratios as mediators instead of the single metabolites. Although we found statistically significant indirect effect estimates of dietary intake on HRQoL outcomes through the KA/QA ratio and the HKr in single mediator models (Supplementary Tables 3 and 4), it should be noted that the ratios reflect only a subset of metabolites, while all metabolites of the KP are hypothesized to causally affect one another. Nevertheless, our previous work demonstrated that the established KP ratios often tend to show robust associations with both diet^12^, ^14^ and outcomes^19^ in CRC survivors, underscoring their relevance as mediators. However, effect estimates from mediator models that include a ratio as a mediator should be interpreted with caution, as the consistency assumption may be violated,^48^ and they may suffer from bias away from the null and inflated type I error rates.^49^

Interestingly, our results do show significant direct effects of dietary intake on HRQoL outcomes, indicating that there are likely also alternative mechanisms explaining the association between dietary intake and HRQoL. Further research is warranted to unravel other mechanisms, including gut microbiota, amino acids, inflammatory markers, or energy metabolism, that can explain the dietary intake—HRQoL relationship in CRC survivors and other populations as well.

## 5 STRENGTHS AND LIMITATIONS

The major strength of this study lies in its prospective design with repeated measurements on dietary intake, plasma kynurenines, HRQoL, and confounders. Another strength was the availability of detailed information on exposures, mediators, outcomes, and confounders. Dietary intake was assessed using seven‐day dietary records, which provided us with extensive quantitative data on macro‐ and micronutrient intake and food components to operationalize two dietary pattern adherence scores. In addition, we analyzed a comprehensive panel of all nine KP metabolites in blood samples after an overnight fast, which is important given that kynurenine concentrations are affected even by a light breakfast.^50^ Furthermore, a well‐validated questionnaire to assess HRQoL for cancer survivors was used.^28^ Lastly, high response rates (>90%) were observed for all posttreatment measurement, resulting in limited missing data. Information on dietary intake, HRQoL outcomes, and the blood draw for the analysis of plasma kynurenines were collected at the same time, ensuring no time lag between exposures, mediators, and outcomes.

However, this study also had a few limitations. First, the temporal ordering of the metabolites of the KP was not reflected in their measurement. In theory, the pathway starts with Trp, which is metabolized to Kyn, which is then converted to several other downstream metabolites in the KP. However, in the current study, the metabolites were determined all at once using one blood sample at each follow‐up measurement. Although the metabolites may have causally influenced each other, we could not impose a causal structure. Therefore, we estimated the potential mediating role of the KP based on interventional mediation effects from parallel‐multiple mediator models, instead of serial‐multiple mediator models, which are fairly strict models.^34^ This analytical approach involving interventional mediation effects does not require the assumption that mediators are causally independent, which is required when interpreting the indirect effect estimates as conventional path‐specific indirect effects. By interpreting the mediator‐specific indirect effect estimates as interventional indirect effect estimates, we acknowledge that the mediators may causally affect one another, without imposing a specific causal structure on the mediators.^34^ Second, because of the relatively small sample and the small effect sizes, the statistical power to detect significant mediation effect estimates was likely low. Third, with the large number of statistical tests performed, there is a chance that some of our findings represent false positive findings. Based on both the small sample size and the large number of tests, we recommend repeating similar analyses in larger study populations.

## 6 CONCLUSION

The findings of the present study in CRC survivors using parallel multiple mediator models suggest that when including all currently available KP metabolites simultaneously, generally the KP is not a mediator in the association of dietary intake with HRQoL outcomes in the first year after CRC treatment. In contrast, the KA/QA ratio and HKr significantly mediated overall associations between carbohydrates, protein, alcohol, magnesium, and zinc with HRQoL outcomes in single mediator models. Considering the significant associations between diet and KP, KP and HRQoL outcomes, and diet and HRQoL outcomes, it is recommended to confirm findings in larger prospective observational studies and further elucidate on biological mechanisms underlying the associations of dietary intake with HRQoL.

## AUTHOR CONTRIBUTIONS

**Daniëlle D. B. Holthuijsen:** Conceptualization; methodology; investigation; formal analysis; writing – original draft. **Judith J. M. Rijnhart:** Methodology; formal analysis; writing – original draft. **Eline H. van Roekel:** Conceptualization; methodology; investigation; supervision; writing – review and editing. **Martijn J. L. Bours:** Conceptualization; methodology; supervision; writing – review and editing. **Per M. Ueland:** Conceptualization; resources; writing – review and editing. **Stéphanie O. Breukink:** Writing – review and editing. **Maryska L. G. Janssen‐Heijnen:** Writing – review and editing. **Joop L. Konsten:** Writing – review and editing. **Eric T. P. Keulen:** Writing – review and editing. **Adrian McCann:** Resources; writing – review and editing. **Stefanie Brezina:** Writing – review and editing. **Biljana Gigic:** Writing – review and editing. **Jennifer Ose:** Writing – review and editing. **Matty P. Weijenberg:** Conceptualization; methodology; funding acquisition; supervision; writing – review and editing. **Simone J. P. M. Eussen:** Conceptualization; methodology; supervision; funding acquisition; writing – review and editing.

## FUNDING INFORMATION

Funding for grant (WCRF: IIG_FULL_2020_018, recipient—S.J.P.M. Eussen) was obtained from Wereld Kanker Onderzoek Fonds (WKOF) as part of the World Cancer Research Fund International (WCRF) grant program. D.D.B. Holthuijsen is supported by this grant. The EnCoRe study was also supported by several other grants from Kankeronderzoekfonds Limburg as part of Health Foundation Limburg (grant 00005739, recipient—M.P. Weijenberg), Stichting Alpe d'HuZes within the research program “Leven met kanker” of the Dutch Cancer Society grants UM 2010–4867 (recipient—M.P. Weijenberg) and UM 2012‐5653 (recipient—M.P. Weijenberg), and by ERA‐NET on Translational Cancer Research (TRANSCAN: Dutch Cancer Society [UM 2014‐6877, recipient—M.P. Weijenberg]), by Wereld Kanker Onderzoek Fonds (WKOF)/World Cancer Research Fund International (WCRF) (grant number 2017/1619—recipient M.J.L. Bours; grant number 2016/1620—recipient M.P. Weijenberg; and grant number SG_2021_076—recipient M.P. Weijenberg). E.H. van Roekel is supported by the Dutch Cancer Society (grant number 2021‐1/13387). B. Gigic was funded by TRANSCAN: German Ministry of Education and Research (BMBF) project 01KT1503, BMBF project 01KD2101D, and the National Institutes of Health/National Cancer Institute (NHI/NCI) projects R01 CA189184 and U01 CA206110. Jennifer Ose was supported by grants from the National Institutes of Health/National Cancer Institute (U01 CA206110, R01 CA189184, R01 CA207371, R01 CA211705, R01 CA254108, R03CA270473), the Huntsman Cancer Foundation.

## CONFLICT OF INTEREST STATEMENT

The authors declare no conflict of interest.

## ETHICS STATEMENT

The study was approved by the Medical Ethics Committee of the University Hospital Maastricht and Maastricht University (Dutch Trial Register no. NL6904). All participants signed written informed consent prior to participation.

## Supporting information


**Data S1** Supplementary materials

## Data Availability

All source code is publicly available in the Supplementary Materials of this paper. The data that support the findings of this study are available from the corresponding author and Dr. Martijn Bours (email: m.bours@maastrichtuniversity.nl) upon reasonable request.
