# Supplementary material for: The mediating role of the kynurenine pathway in longitudinal associations between dietary intake and quality of life in colorectal cancer survivors up to 12 months posttreatment
Source: Int J Cancer. 2026 Feb 24;159(1):122–43. doi: 10.1002/ijc.70363 (PMC13140012; doi:10.1002/ijc.70363)
Supplement: Supplementary file 1 — Data S1 Supplementary materials [file IJC-159-122-s001.pdf]

The mediating role of the kynurenine pathway in longitudinal associations between dietary intake and quality of life in colorectal cancer survivors up to 12 months posttreatment

Daniëlle D.B. Holthuijsen, Judith J.M. Rijnhart, Eline H. van Roekel, Martijn J.L. Bours, Per M. Ueland, Stéphanie O. Breukink, Maryska L.G. Janssen-Heijnen, Joop L. Konsten, Eric T.P. Keulen, Adrian McCann, Stefanie Brezina, Biljana Gigic, Jennifer Ose, Matty P. Weijenberg, and Simone J.P.M. Eussen

Table of contents

1. Supplementary materials & methods 2
1.1 Parallel-multiple mediator model: Macronutrient intake – Physical functioning 2
1.2 Parallel-multiple mediator model: Micronutrient intake – Physical functioning 10
1.3 Parallel-multiple mediator model: Dietary pattern – Physical functioning 17
1.4 Single mediator model: Macronutrient intake – Physical functioning 24
1.5 Single mediator model: Micronutrient intake – Physical functioning 26
1.6 Single mediator model: Dietary pattern – Physical functioning 28
2. **Supplementary Tables** 30
Supplementary Table 1. 30
Supplementary Table 2. 35
Supplementary Table 3. 37
Supplementary Table 4. 39
3. **Supplementary Figures** 41
Supplementary Figure 1 41
Supplementary Figure 2 42

1. Supplementary materials & methods

1.1 Parallel-multiple mediator model: Macronutrient intake – Physical functioning

```
###Macronutrient intake – Physical functioning

### Estimate mediator model and save coefficients ###
# Mediator models
modela1 <- (lmer(Trp ~ Koolhydraten_kcal_day_SD + Vet_kcal_day_SD + Eiwit_kcal_day_SD +
Alcohol_kcal_day_SD + Voedingsvezel_kcal_day_SD + ENROLL_AGE + SEX + Creat +
COM_NUMBER_CAT + P_CLIN_CHEM + WEEKS_SINCE_END_TREAT + STOMA +
W0EDU_LEVEL + BMI + MVPA + SMOKER_CURR2 + mox.mdursedge30min_h + (1 |PATIENTN),
data=Dataset_Sumsc))
summary(modela1)
confint(modela1)

modela2 <- (lmer(Kyn ~ Koolhydraten_kcal_day_SD + Vet_kcal_day_SD + Eiwit_kcal_day_SD +
Alcohol_kcal_day_SD + Voedingsvezel_kcal_day_SD + ENROLL_AGE + SEX + Creat +
COM_NUMBER_CAT + P_CLIN_CHEM + WEEKS_SINCE_END_TREAT + STOMA +
W0EDU_LEVEL + BMI + MVPA + SMOKER_CURR2 + mox.mdursedge30min_h + (1 |PATIENTN),
data=Dataset_Sumsc))
summary(modela2)
confint(modela2)

modela3 <- (lmer(HK ~ Koolhydraten_kcal_day_SD + Vet_kcal_day_SD + Eiwit_kcal_day_SD +
Alcohol_kcal_day_SD + Voedingsvezel_kcal_day_SD + ENROLL_AGE + SEX + Creat +
COM_NUMBER_CAT + P_CLIN_CHEM + WEEKS_SINCE_END_TREAT + STOMA +
W0EDU_LEVEL + BMI + MVPA + SMOKER_CURR2 + mox.mdursedge30min_h + (1 |PATIENTN),
data=Dataset_Sumsc))
summary(modela3)
confint(modela3)

modela4 <- (lmer(KA ~ Koolhydraten_kcal_day_SD + Vet_kcal_day_SD + Eiwit_kcal_day_SD +
Alcohol_kcal_day_SD + Voedingsvezel_kcal_day_SD + ENROLL_AGE + SEX + Creat +
COM_NUMBER_CAT + P_CLIN_CHEM + WEEKS_SINCE_END_TREAT + STOMA +
W0EDU_LEVEL + BMI + MVPA + SMOKER_CURR2 + mox.mdursedge30min_h + (1 |PATIENTN),
data=Dataset_Sumsc))
summary(modela4)
confint(modela4)

modela5 <- (lmer(XA ~ Koolhydraten_kcal_day_SD + Vet_kcal_day_SD + Eiwit_kcal_day_SD +
Alcohol_kcal_day_SD + Voedingsvezel_kcal_day_SD + ENROLL_AGE + SEX + Creat +
COM_NUMBER_CAT + P_CLIN_CHEM + WEEKS_SINCE_END_TREAT + STOMA +
W0EDU_LEVEL + BMI + MVPA + SMOKER_CURR2 + mox.mdursedge30min_h + (1 |PATIENTN),
data=Dataset_Sumsc))
summary(modela5)
confint(modela5)

modela6 <- (lmer(AA ~ Koolhydraten_kcal_day_SD + Vet_kcal_day_SD + Eiwit_kcal_day_SD +
Alcohol_kcal_day_SD + Voedingsvezel_kcal_day_SD + ENROLL_AGE + SEX + Creat +
COM_NUMBER_CAT + P_CLIN_CHEM + WEEKS_SINCE_END_TREAT + STOMA +
W0EDU_LEVEL + BMI + MVPA + SMOKER_CURR2 + mox.mdursedge30min_h + (1 |PATIENTN),
data=Dataset_Sumsc))
summary(modela6)
confint(modela6)
```

```
modela7 <- (lmer(HAA ~ Koolhydraten_kcal_day_SD + Vet_kcal_day_SD + Eiwit_kcal_day_SD +
Alcohol_kcal_day_SD + Voedingsvezel_kcal_day_SD + ENROLL_AGE + SEX + Creat +
COM_NUMBER_CAT + P_CLIN_CHEM + WEEKS_SINCE_END_TREAT + STOMA +
W0EDU_LEVEL + BMI + MVPA + SMOKER_CURR2 + mox.mdursedge30min_h + (1 |PATIENTN),
data=Dataset_Sumsc))
summary(modela7)
confint(modela7)

modela8 <- (lmer(Pic ~ Koolhydraten_kcal_day_SD + Vet_kcal_day_SD + Eiwit_kcal_day_SD +
Alcohol_kcal_day_SD + Voedingsvezel_kcal_day_SD + ENROLL_AGE + SEX + Creat +
COM_NUMBER_CAT + P_CLIN_CHEM + WEEKS_SINCE_END_TREAT + STOMA +
W0EDU_LEVEL + BMI + MVPA + SMOKER_CURR2 + mox.mdursedge30min_h + (1 |PATIENTN),
data=Dataset_Sumsc))
summary(modela8)
confint(modela8)

modela9 <- (lmer(QA ~ Koolhydraten_kcal_day_SD + Vet_kcal_day_SD + Eiwit_kcal_day_SD +
Alcohol_kcal_day_SD + Voedingsvezel_kcal_day_SD + ENROLL_AGE + SEX + Creat +
COM_NUMBER_CAT + P_CLIN_CHEM + WEEKS_SINCE_END_TREAT + STOMA +
W0EDU_LEVEL + BMI + MVPA + SMOKER_CURR2 + mox.mdursedge30min_h + (1 |PATIENTN),
data=Dataset_Sumsc))
summary(modela9)
confint(modela9)

# Save a coefficients and variance
a1 <- modela1@beta[2]
Sa1 <- vcov(modela1) ["Koolhydraten_kcal_day_SD", "Koolhydraten_kcal_day_SD"]

a2 <- modela2@beta[2]
Sa2 <- vcov(modela2) ["Koolhydraten_kcal_day_SD", "Koolhydraten_kcal_day_SD"]

a3 <- modela3@beta[2]
Sa3 <- vcov(modela3) ["Koolhydraten_kcal_day_SD", "Koolhydraten_kcal_day_SD"]

a4 <- modela4@beta[2]
Sa4 <- vcov(modela4) ["Koolhydraten_kcal_day_SD", "Koolhydraten_kcal_day_SD"]

a5 <- modela5@beta[2]
Sa5 <- vcov(modela5) ["Koolhydraten_kcal_day_SD", "Koolhydraten_kcal_day_SD"]

a6 <- modela6@beta[2]
Sa6 <- vcov(modela6) ["Koolhydraten_kcal_day_SD", "Koolhydraten_kcal_day_SD"]

a7 <- modela7@beta[2]
Sa7 <- vcov(modela7) ["Koolhydraten_kcal_day_SD", "Koolhydraten_kcal_day_SD"]

a8 <- modela8@beta[2]
Sa8 <- vcov(modela8) ["Koolhydraten_kcal_day_SD", "Koolhydraten_kcal_day_SD"]

a9 <- modela9@beta[2]
Sa9 <- vcov(modela9) ["Koolhydraten_kcal_day_SD", "Koolhydraten_kcal_day_SD"]


### Estimate outcome model and save coefficients ###
# Outcome model
```

```
modelb <- (lmer(PF2 ~ Koolhydraten_kcal_day_SD + Vet_kcal_day_SD + Eiwit_kcal_day_SD +
Alcohol_kcal_day_SD + Voedingsvezel_kcal_day_SD + Trp + Kyn + HK + KA + XA + AA + HAA + Pic
+ QA + ENROLL_AGE + SEX + Creat + COM_NUMBER_CAT + P_CLIN_CHEM +
WEEKS_SINCE_END_TREAT + STOMA + W0EDU_LEVEL + BMI + MVPA + SMOKER_CURR2 +
mox.mdursedge30min_h + (1 |PATIENTN), data=Dataset_Sumsc))
summary(modelb)
confint(modelb)

# Save c' coefficient and variance
cp <- modelb@beta[2]
Scp <- vcov(modelb) ["Koolhydraten_kcal_day_SD", "Koolhydraten_kcal_day_SD"]

# Save b coefficient and variance
b1 <- modelb@beta[7]
Sb1 <- vcov(modelb) ["Trp", "Trp"]
Covsb1b2 <- vcov(modelb) ["Trp", "Kyn"]
Covsb1b3 <- vcov(modelb) ["Trp", "HK"]
Covsb1b4 <- vcov(modelb) ["Trp", "KA"]
Covsb1b5 <- vcov(modelb) ["Trp", "XA"]
Covsb1b6 <- vcov(modelb) ["Trp", "AA"]
Covsb1b7 <- vcov(modelb) ["Trp", "HAA"]
Covsb1b8 <- vcov(modelb) ["Trp", "Pic"]
Covsb1b9 <- vcov(modelb) ["Trp", "QA"]
Covsb1cp <- vcov(modelb) ["Trp", "Koolhydraten_kcal_day_SD"]

b2 <- modelb@beta[8]
Sb2 <- vcov(modelb) ["Kyn", "Kyn"]
Covsb2b3 <- vcov(modelb) ["Kyn", "HK"]
Covsb2b4 <- vcov(modelb) ["Kyn", "KA"]
Covsb2b5 <- vcov(modelb) ["Kyn", "XA"]
Covsb2b6 <- vcov(modelb) ["Kyn", "AA"]
Covsb2b7 <- vcov(modelb) ["Kyn", "HAA"]
Covsb2b8 <- vcov(modelb) ["Kyn", "Pic"]
Covsb2b9 <- vcov(modelb) ["Kyn", "QA"]
Covsb2cp <- vcov(modelb) ["Kyn", "Koolhydraten_kcal_day_SD"]

b3 <- modelb@beta[9]
Sb3 <- vcov(modelb) ["HK", "HK"]
Covsb3b4 <- vcov(modelb) ["HK", "KA"]
Covsb3b5 <- vcov(modelb) ["HK", "XA"]
Covsb3b6 <- vcov(modelb) ["HK", "AA"]
Covsb3b7 <- vcov(modelb) ["HK", "HAA"]
Covsb3b8 <- vcov(modelb) ["HK", "Pic"]
Covsb3b9 <- vcov(modelb) ["HK", "QA"]
Covsb3cp <- vcov(modelb) ["HK", "Koolhydraten_kcal_day_SD"]

b4 <- modelb@beta[10]
Sb4 <- vcov(modelb) ["KA", "KA"]
Covsb4b5 <- vcov(modelb) ["KA", "XA"]
Covsb4b6 <- vcov(modelb) ["KA", "AA"]
Covsb4b7 <- vcov(modelb) ["KA", "HAA"]
Covsb4b8 <- vcov(modelb) ["KA", "Pic"]
Covsb4b9 <- vcov(modelb) ["KA", "QA"]
Covsb4cp <- vcov(modelb) ["KA", "Koolhydraten_kcal_day_SD"]

b5 <- modelb@beta[11]
```

```
Sb5 <- vcov(modelb) ["XA", "XA"]
Covsb5b6 <- vcov(modelb) ["XA", "AA"]
Covsb5b7 <- vcov(modelb) ["XA", "HAA"]
Covsb5b8 <- vcov(modelb) ["XA", "Pic"]
Covsb5b9 <- vcov(modelb) ["XA", "QA"]
Covsb5cp <- vcov(modelb) ["XA", "Koolhydraten_kcal_day_SD"]

b6 <- modelb@beta[12]
Sb6 <- vcov(modelb) ["AA", "AA"]
Covsb6b7 <- vcov(modelb) ["AA", "HAA"]
Covsb6b8 <- vcov(modelb) ["AA", "Pic"]
Covsb6b9 <- vcov(modelb) ["AA", "QA"]
Covsb6cp <- vcov(modelb) ["AA", "Koolhydraten_kcal_day_SD"]

b7 <- modelb@beta[13]
Sb7 <- vcov(modelb) ["HAA", "HAA"]
Covsb7b8 <- vcov(modelb) ["HAA", "Pic"]
Covsb7b9 <- vcov(modelb) ["HAA", "QA"]
Covsb7cp <- vcov(modelb) ["HAA", "Koolhydraten_kcal_day_SD"]

b8 <- modelb@beta[14]
Sb8 <- vcov(modelb) ["Pic", "Pic"]
Covsb8b9 <- vcov(modelb) ["Pic", "QA"]
Covsb8cp <- vcov(modelb) ["Pic", "Koolhydraten_kcal_day_SD"]

b9 <- modelb@beta[15]
Sb9 <- vcov(modelb) ["QA", "QA"]
Covsb9cp <- vcov(modelb) ["QA", "Koolhydraten_kcal_day_SD"]

### Compute mediation effects with confidence intervals ###
# Indirect effect with Monte Carlo confidence interval
ab1 <- a1*b1
ab1
set.seed(1234)
ci(mu=c(b1=a1, b2=a2, b3=a3, b4=a4, b5=a5, b6=a6, b7=a7, b8=a8, b9=a9, b10=b1, b11=b2,
b12=b3, b13=b4, b14=b5, b15=b6, b16=b7, b17=b8, b18=b9, b19=cp),
Sigma=c(Sa1,0,0,0,0,0,0,0,0,0,0,0,0,0,0,0,0,0,0,Sa2,0,0,0,0,0,0,0,0,0,0,0,0,0,0,0,0,0,Sa3,0,0,0,0,0,0,0
,0,0,0,0,0,0,0,0,0,Sa4,0,0,0,0,0,0,0,0,0,0,0,0,0,0,0,Sa5,
0,0,0,0,0,0,0,0,0,0,0,0,0,0,Sa6,0,0,0,0,0,0,0,0,0,0,0,0,0,Sa7,0,0,0,0,0,0,0,0,0,0,0,0,Sa8,0,0,0,0,0,0,0,0
,0,0,0,Sa9,0,0,0,0,0,0,0,0,0,0,Sb1,Covsb1b2,Covsb1b3,Covsb1b4,Covsb1b5,Covsb1b6,Covsb1b7,Co
vsb1b8,Covsb1b9,Covsb1cp,Sb2,Covsb2b3,Covsb2b4,Covsb2b5,Covsb2b6,Covsb2b7,Covsb2b8,Co
vsb2b9,Covsb2cp,Sb3,Covsb3b4,Covsb3b5,Covsb3b6,Covsb3b7,Covsb3b8,Covsb3b9,Covsb3cp,Sb
4,Covsb4b5,Covsb4b6,Covsb4b7,Covsb4b8,Covsb4b9,Covsb4cp,Sb5,Covsb5b6,Covsb5b7,Covsb5b
8,Covsb5b9,Covsb5cp,Sb6,Covsb6b7,Covsb6b8,Covsb6b9,Covsb6cp,Sb7,Covsb7b8,Covsb7b9,Cov
sb7cp,Sb8,Covsb8b9,Covsb8cp,Sb9,Covsb9cp,Scp),
  quant=~b1*b10, alpha=0.05, type="MC", n.mc=20000)

ab2 <- a2*b2
ab2
set.seed(1234)
ci(mu=c(b1=a1, b2=a2, b3=a3, b4=a4, b5=a5, b6=a6, b7=a7, b8=a8, b9=a9, b10=b1, b11=b2,
b12=b3, b13=b4, b14=b5, b15=b6, b16=b7, b17=b8, b18=b9, b19=cp),
Sigma=c(Sa1,0,0,0,0,0,0,0,0,0,0,0,0,0,0,0,0,0,0,Sa2,0,0,0,0,0,0,0,0,0,0,0,0,0,0,0,0,0,Sa3,0,0,0,0,0,0,0
,0,0,0,0,0,0,0,0,0,Sa4,0,0,0,0,0,0,0,0,0,0,0,0,0,0,0,Sa5,
0,0,0,0,0,0,0,0,0,0,0,0,0,0,Sa6,0,0,0,0,0,0,0,0,0,0,0,0,0,Sa7,0,0,0,0,0,0,0,0,0,0,0,0,Sa8,0,0,0,0,0,0,0,0
,0,0,0,Sa9,0,0,0,0,0,0,0,0,0,0,Sb1,Covsb1b2,Covsb1b3,Covsb1b4,Covsb1b5,Covsb1b6,Covsb1b7,Co
```

```
vsb1b8,Covsb1b9,Covsb1cp,Sb2,Covsb2b3,Covsb2b4,Covsb2b5,Covsb2b6,Covsb2b7,Covsb2b8,Co
vsb2b9,Covsb2cp,Sb3,Covsb3b4,Covsb3b5,Covsb3b6,Covsb3b7,Covsb3b8,Covsb3b9,Covsb3cp,Sb
4,Covsb4b5,Covsb4b6,Covsb4b7,Covsb4b8,Covsb4b9,Covsb4cp,Sb5,Covsb5b6,Covsb5b7,Covsb5b
8,Covsb5b9,Covsb5cp,Sb6,Covsb6b7,Covsb6b8,Covsb6b9,Covsb6cp,Sb7,Covsb7b8,Covsb7b9,Cov
sb7cp,Sb8,Covsb8b9,Covsb8cp,Sb9,Covsb9cp,Scp),
  quant=~b2*b11, alpha=0.05, type="MC", n.mc=20000)

ab3 <- a3*b3
ab3
set.seed(1234)
ci(mu=c(b1=a1, b2=a2, b3=a3, b4=a4, b5=a5, b6=a6, b7=a7, b8=a8, b9=a9, b10=b1, b11=b2,
b12=b3, b13=b4, b14=b5, b15=b6, b16=b7, b17=b8, b18=b9, b19=cp),
Sigma=c(Sa1,0,0,0,0,0,0,0,0,0,0,0,0,0,0,0,0,0,0,Sa2,0,0,0,0,0,0,0,0,0,0,0,0,0,0,0,0,0,Sa3,0,0,0,0,0,0,0
,0,0,0,0,0,0,0,0,0,Sa4,0,0,0,0,0,0,0,0,0,0,0,0,0,0,0,Sa5,
0,0,0,0,0,0,0,0,0,0,0,0,0,0,Sa6,0,0,0,0,0,0,0,0,0,0,0,0,0,Sa7,0,0,0,0,0,0,0,0,0,0,0,0,Sa8,0,0,0,0,0,0,0,0
,0,0,0,Sa9,0,0,0,0,0,0,0,0,0,0,Sb1,Covsb1b2,Covsb1b3,Covsb1b4,Covsb1b5,Covsb1b6,Covsb1b7,Co
vsb1b8,Covsb1b9,Covsb1cp,Sb2,Covsb2b3,Covsb2b4,Covsb2b5,Covsb2b6,Covsb2b7,Covsb2b8,Co
vsb2b9,Covsb2cp,Sb3,Covsb3b4,Covsb3b5,Covsb3b6,Covsb3b7,Covsb3b8,Covsb3b9,Covsb3cp,Sb
4,Covsb4b5,Covsb4b6,Covsb4b7,Covsb4b8,Covsb4b9,Covsb4cp,Sb5,Covsb5b6,Covsb5b7,Covsb5b
8,Covsb5b9,Covsb5cp,Sb6,Covsb6b7,Covsb6b8,Covsb6b9,Covsb6cp,Sb7,Covsb7b8,Covsb7b9,Cov
sb7cp,Sb8,Covsb8b9,Covsb8cp,Sb9,Covsb9cp,Scp),
  quant=~b3*b12, alpha=0.05, type="MC", n.mc=20000)

ab4 <- a4*b4
ab4
set.seed(1234)
ci(mu=c(b1=a1, b2=a2, b3=a3, b4=a4, b5=a5, b6=a6, b7=a7, b8=a8, b9=a9, b10=b1, b11=b2,
b12=b3, b13=b4, b14=b5, b15=b6, b16=b7, b17=b8, b18=b9, b19=cp),
Sigma=c(Sa1,0,0,0,0,0,0,0,0,0,0,0,0,0,0,0,0,0,0,Sa2,0,0,0,0,0,0,0,0,0,0,0,0,0,0,0,0,0,Sa3,0,0,0,0,0,0,0
,0,0,0,0,0,0,0,0,0,Sa4,0,0,0,0,0,0,0,0,0,0,0,0,0,0,0,Sa5,
0,0,0,0,0,0,0,0,0,0,0,0,0,0,Sa6,0,0,0,0,0,0,0,0,0,0,0,0,0,Sa7,0,0,0,0,0,0,0,0,0,0,0,0,Sa8,0,0,0,0,0,0,0,0
,0,0,0,Sa9,0,0,0,0,0,0,0,0,0,0,Sb1,Covsb1b2,Covsb1b3,Covsb1b4,Covsb1b5,Covsb1b6,Covsb1b7,Co
vsb1b8,Covsb1b9,Covsb1cp,Sb2,Covsb2b3,Covsb2b4,Covsb2b5,Covsb2b6,Covsb2b7,Covsb2b8,Co
vsb2b9,Covsb2cp,Sb3,Covsb3b4,Covsb3b5,Covsb3b6,Covsb3b7,Covsb3b8,Covsb3b9,Covsb3cp,Sb
4,Covsb4b5,Covsb4b6,Covsb4b7,Covsb4b8,Covsb4b9,Covsb4cp,Sb5,Covsb5b6,Covsb5b7,Covsb5b
8,Covsb5b9,Covsb5cp,Sb6,Covsb6b7,Covsb6b8,Covsb6b9,Covsb6cp,Sb7,Covsb7b8,Covsb7b9,Cov
sb7cp,Sb8,Covsb8b9,Covsb8cp,Sb9,Covsb9cp,Scp),
  quant=~b4*b13, alpha=0.05, type="MC", n.mc=20000)

ab5 <- a5*b5
ab5
set.seed(1234)
ci(mu=c(b1=a1, b2=a2, b3=a3, b4=a4, b5=a5, b6=a6, b7=a7, b8=a8, b9=a9, b10=b1, b11=b2,
b12=b3, b13=b4, b14=b5, b15=b6, b16=b7, b17=b8, b18=b9, b19=cp),
Sigma=c(Sa1,0,0,0,0,0,0,0,0,0,0,0,0,0,0,0,0,0,0,Sa2,0,0,0,0,0,0,0,0,0,0,0,0,0,0,0,0,0,Sa3,0,0,0,0,0,0,0
,0,0,0,0,0,0,0,0,0,Sa4,0,0,0,0,0,0,0,0,0,0,0,0,0,0,0,Sa5,
0,0,0,0,0,0,0,0,0,0,0,0,0,0,Sa6,0,0,0,0,0,0,0,0,0,0,0,0,0,Sa7,0,0,0,0,0,0,0,0,0,0,0,0,Sa8,0,0,0,0,0,0,0,0
,0,0,0,Sa9,0,0,0,0,0,0,0,0,0,0,Sb1,Covsb1b2,Covsb1b3,Covsb1b4,Covsb1b5,Covsb1b6,Covsb1b7,Co
vsb1b8,Covsb1b9,Covsb1cp,Sb2,Covsb2b3,Covsb2b4,Covsb2b5,Covsb2b6,Covsb2b7,Covsb2b8,Co
vsb2b9,Covsb2cp,Sb3,Covsb3b4,Covsb3b5,Covsb3b6,Covsb3b7,Covsb3b8,Covsb3b9,Covsb3cp,Sb
4,Covsb4b5,Covsb4b6,Covsb4b7,Covsb4b8,Covsb4b9,Covsb4cp,Sb5,Covsb5b6,Covsb5b7,Covsb5b
8,Covsb5b9,Covsb5cp,Sb6,Covsb6b7,Covsb6b8,Covsb6b9,Covsb6cp,Sb7,Covsb7b8,Covsb7b9,Cov
sb7cp,Sb8,Covsb8b9,Covsb8cp,Sb9,Covsb9cp,Scp),
  quant=~b5*b14, alpha=0.05, type="MC", n.mc=20000)

ab6 <- a6*b6
```

```
ab6
set.seed(1234)
ci(mu=c(b1=a1, b2=a2, b3=a3, b4=a4, b5=a5, b6=a6, b7=a7, b8=a8, b9=a9, b10=b1, b11=b2,
b12=b3, b13=b4, b14=b5, b15=b6, b16=b7, b17=b8, b18=b9, b19=cp),
Sigma=c(Sa1,0,0,0,0,0,0,0,0,0,0,0,0,0,0,0,0,0,0,Sa2,0,0,0,0,0,0,0,0,0,0,0,0,0,0,0,0,0,Sa3,0,0,0,0,0,0,0,0
,0,0,0,0,0,0,0,0,0,Sa4,0,0,0,0,0,0,0,0,0,0,0,0,0,0,0,Sa5,
0,0,0,0,0,0,0,0,0,0,0,0,0,0,Sa6,0,0,0,0,0,0,0,0,0,0,0,0,0,Sa7,0,0,0,0,0,0,0,0,0,0,0,0,Sa8,0,0,0,0,0,0,0,0
,0,0,0,Sa9,0,0,0,0,0,0,0,0,0,0,Sb1,Covsb1b2,Covsb1b3,Covsb1b4,Covsb1b5,Covsb1b6,Covsb1b7,Co
vsb1b8,Covsb1b9,Covsb1cp,Sb2,Covsb2b3,Covsb2b4,Covsb2b5,Covsb2b6,Covsb2b7,Covsb2b8,Co
vsb2b9,Covsb2cp,Sb3,Covsb3b4,Covsb3b5,Covsb3b6,Covsb3b7,Covsb3b8,Covsb3b9,Covsb3cp,Sb
4,Covsb4b5,Covsb4b6,Covsb4b7,Covsb4b8,Covsb4b9,Covsb4cp,Sb5,Covsb5b6,Covsb5b7,Covsb5b
8,Covsb5b9,Covsb5cp,Sb6,Covsb6b7,Covsb6b8,Covsb6b9,Covsb6cp,Sb7,Covsb7b8,Covsb7b9,Cov
sb7cp,Sb8,Covsb8b9,Covsb8cp,Sb9,Covsb9cp,Scp),
  quant=~b6*b15, alpha=0.05, type="MC", n.mc=20000)

ab7 <- a7*b7
ab7
set.seed(1234)
ci(mu=c(b1=a1, b2=a2, b3=a3, b4=a4, b5=a5, b6=a6, b7=a7, b8=a8, b9=a9, b10=b1, b11=b2,
b12=b3, b13=b4, b14=b5, b15=b6, b16=b7, b17=b8, b18=b9, b19=cp),
Sigma=c(Sa1,0,0,0,0,0,0,0,0,0,0,0,0,0,0,0,0,0,0,Sa2,0,0,0,0,0,0,0,0,0,0,0,0,0,0,0,0,0,Sa3,0,0,0,0,0,0,0,0
,0,0,0,0,0,0,0,0,0,Sa4,0,0,0,0,0,0,0,0,0,0,0,0,0,0,0,Sa5,
0,0,0,0,0,0,0,0,0,0,0,0,0,0,Sa6,0,0,0,0,0,0,0,0,0,0,0,0,0,Sa7,0,0,0,0,0,0,0,0,0,0,0,0,Sa8,0,0,0,0,0,0,0,0
,0,0,0,Sa9,0,0,0,0,0,0,0,0,0,0,Sb1,Covsb1b2,Covsb1b3,Covsb1b4,Covsb1b5,Covsb1b6,Covsb1b7,Co
vsb1b8,Covsb1b9,Covsb1cp,Sb2,Covsb2b3,Covsb2b4,Covsb2b5,Covsb2b6,Covsb2b7,Covsb2b8,Co
vsb2b9,Covsb2cp,Sb3,Covsb3b4,Covsb3b5,Covsb3b6,Covsb3b7,Covsb3b8,Covsb3b9,Covsb3cp,Sb
4,Covsb4b5,Covsb4b6,Covsb4b7,Covsb4b8,Covsb4b9,Covsb4cp,Sb5,Covsb5b6,Covsb5b7,Covsb5b
8,Covsb5b9,Covsb5cp,Sb6,Covsb6b7,Covsb6b8,Covsb6b9,Covsb6cp,Sb7,Covsb7b8,Covsb7b9,Cov
sb7cp,Sb8,Covsb8b9,Covsb8cp,Sb9,Covsb9cp,Scp),
  quant=~b7*b16, alpha=0.05, type="MC", n.mc=20000)

ab8 <- a8*b8
ab8
set.seed(1234)
ci(mu=c(b1=a1, b2=a2, b3=a3, b4=a4, b5=a5, b6=a6, b7=a7, b8=a8, b9=a9, b10=b1, b11=b2,
b12=b3, b13=b4, b14=b5, b15=b6, b16=b7, b17=b8, b18=b9, b19=cp),
Sigma=c(Sa1,0,0,0,0,0,0,0,0,0,0,0,0,0,0,0,0,0,0,Sa2,0,0,0,0,0,0,0,0,0,0,0,0,0,0,0,0,0,Sa3,0,0,0,0,0,0,0,0
,0,0,0,0,0,0,0,0,0,Sa4,0,0,0,0,0,0,0,0,0,0,0,0,0,0,0,Sa5,
0,0,0,0,0,0,0,0,0,0,0,0,0,0,Sa6,0,0,0,0,0,0,0,0,0,0,0,0,0,Sa7,0,0,0,0,0,0,0,0,0,0,0,0,Sa8,0,0,0,0,0,0,0,0
,0,0,0,Sa9,0,0,0,0,0,0,0,0,0,0,Sb1,Covsb1b2,Covsb1b3,Covsb1b4,Covsb1b5,Covsb1b6,Covsb1b7,Co
vsb1b8,Covsb1b9,Covsb1cp,Sb2,Covsb2b3,Covsb2b4,Covsb2b5,Covsb2b6,Covsb2b7,Covsb2b8,Co
vsb2b9,Covsb2cp,Sb3,Covsb3b4,Covsb3b5,Covsb3b6,Covsb3b7,Covsb3b8,Covsb3b9,Covsb3cp,Sb
4,Covsb4b5,Covsb4b6,Covsb4b7,Covsb4b8,Covsb4b9,Covsb4cp,Sb5,Covsb5b6,Covsb5b7,Covsb5b
8,Covsb5b9,Covsb5cp,Sb6,Covsb6b7,Covsb6b8,Covsb6b9,Covsb6cp,Sb7,Covsb7b8,Covsb7b9,Cov
sb7cp,Sb8,Covsb8b9,Covsb8cp,Sb9,Covsb9cp,Scp),
  quant=~b8*b17, alpha=0.05, type="MC", n.mc=20000)

ab9 <- a9*b9
ab9
set.seed(1234)
ci(mu=c(b1=a1, b2=a2, b3=a3, b4=a4, b5=a5, b6=a6, b7=a7, b8=a8, b9=a9, b10=b1, b11=b2,
b12=b3, b13=b4, b14=b5, b15=b6, b16=b7, b17=b8, b18=b9, b19=cp),
Sigma=c(Sa1,0,0,0,0,0,0,0,0,0,0,0,0,0,0,0,0,0,0,Sa2,0,0,0,0,0,0,0,0,0,0,0,0,0,0,0,0,0,Sa3,0,0,0,0,0,0,0,0
,0,0,0,0,0,0,0,0,0,Sa4,0,0,0,0,0,0,0,0,0,0,0,0,0,0,0,Sa5,
0,0,0,0,0,0,0,0,0,0,0,0,0,0,Sa6,0,0,0,0,0,0,0,0,0,0,0,0,0,Sa7,0,0,0,0,0,0,0,0,0,0,0,0,Sa8,0,0,0,0,0,0,0,0
,0,0,0,Sa9,0,0,0,0,0,0,0,0,0,0,Sb1,Covsb1b2,Covsb1b3,Covsb1b4,Covsb1b5,Covsb1b6,Covsb1b7,Co
```

```
vsb1b8,Covsb1b9,Covsb1cp,Sb2,Covsb2b3,Covsb2b4,Covsb2b5,Covsb2b6,Covsb2b7,Covsb2b8,Co
vsb2b9,Covsb2cp,Sb3,Covsb3b4,Covsb3b5,Covsb3b6,Covsb3b7,Covsb3b8,Covsb3b9,Covsb3cp,Sb
4,Covsb4b5,Covsb4b6,Covsb4b7,Covsb4b8,Covsb4b9,Covsb4cp,Sb5,Covsb5b6,Covsb5b7,Covsb5b
8,Covsb5b9,Covsb5cp,Sb6,Covsb6b7,Covsb6b8,Covsb6b9,Covsb6cp,Sb7,Covsb7b8,Covsb7b9,Cov
sb7cp,Sb8,Covsb8b9,Covsb8cp,Sb9,Covsb9cp,Scp),
  quant=~b9*b18, alpha=0.05, type="MC", n.mc=20000)

abtot <- ab1 + ab2 + ab3 + ab4 + ab5 + ab6 + ab7 + ab8 + ab9
abtot
set.seed(1234)
ci(mu=c(b1=a1, b2=a2, b3=a3, b4=a4, b5=a5, b6=a6, b7=a7, b8=a8, b9=a9, b10=b1, b11=b2,
b12=b3, b13=b4, b14=b5, b15=b6, b16=b7, b17=b8, b18=b9, b19=cp),
Sigma=c(Sa1,0,0,0,0,0,0,0,0,0,0,0,0,0,0,0,0,0,0,Sa2,0,0,0,0,0,0,0,0,0,0,0,0,0,0,0,0,0,Sa3,0,0,0,0,0,0,0
,0,0,0,0,0,0,0,0,0,Sa4,0,0,0,0,0,0,0,0,0,0,0,0,0,0,0,Sa5,
0,0,0,0,0,0,0,0,0,0,0,0,0,0,Sa6,0,0,0,0,0,0,0,0,0,0,0,0,0,Sa7,0,0,0,0,0,0,0,0,0,0,0,0,Sa8,0,0,0,0,0,0,0,0
,0,0,0,Sa9,0,0,0,0,0,0,0,0,0,0,Sb1,Covsb1b2,Covsb1b3,Covsb1b4,Covsb1b5,Covsb1b6,Covsb1b7,Co
vsb1b8,Covsb1b9,Covsb1cp,Sb2,Covsb2b3,Covsb2b4,Covsb2b5,Covsb2b6,Covsb2b7,Covsb2b8,Co
vsb2b9,Covsb2cp,Sb3,Covsb3b4,Covsb3b5,Covsb3b6,Covsb3b7,Covsb3b8,Covsb3b9,Covsb3cp,Sb
4,Covsb4b5,Covsb4b6,Covsb4b7,Covsb4b8,Covsb4b9,Covsb4cp,Sb5,Covsb5b6,Covsb5b7,Covsb5b
8,Covsb5b9,Covsb5cp,Sb6,Covsb6b7,Covsb6b8,Covsb6b9,Covsb6cp,Sb7,Covsb7b8,Covsb7b9,Cov
sb7cp,Sb8,Covsb8b9,Covsb8cp,Sb9,Covsb9cp,Scp),
  quant=~b1*b10+b2*b11+b3*b12+b4*b13+b5*b14+ b6*b15+b7*b16+b8*b17+b9*b18, alpha=0.05,
type="MC", n.mc=20000)

# Direct effect with confidence interval
direct <- cp
direct
set.seed(1234)
ci(mu=c(b1=a1, b2=a2, b3=a3, b4=a4, b5=a5, b6=a6, b7=a7, b8=a8, b9=a9, b10=b1, b11=b2,
b12=b3, b13=b4, b14=b5, b15=b6, b16=b7, b17=b8, b18=b9, b19=cp),
Sigma=c(Sa1,0,0,0,0,0,0,0,0,0,0,0,0,0,0,0,0,0,0,Sa2,0,0,0,0,0,0,0,0,0,0,0,0,0,0,0,0,0,Sa3,0,0,0,0,0,0,0
,0,0,0,0,0,0,0,0,0,Sa4,0,0,0,0,0,0,0,0,0,0,0,0,0,0,0,Sa5,
0,0,0,0,0,0,0,0,0,0,0,0,0,0,Sa6,0,0,0,0,0,0,0,0,0,0,0,0,0,Sa7,0,0,0,0,0,0,0,0,0,0,0,0,Sa8,0,0,0,0,0,0,0,0
,0,0,0,Sa9,0,0,0,0,0,0,0,0,0,0,Sb1,Covsb1b2,Covsb1b3,Covsb1b4,Covsb1b5,Covsb1b6,Covsb1b7,Co
vsb1b8,Covsb1b9,Covsb1cp,Sb2,Covsb2b3,Covsb2b4,Covsb2b5,Covsb2b6,Covsb2b7,Covsb2b8,Co
vsb2b9,Covsb2cp,Sb3,Covsb3b4,Covsb3b5,Covsb3b6,Covsb3b7,Covsb3b8,Covsb3b9,Covsb3cp,Sb
4,Covsb4b5,Covsb4b6,Covsb4b7,Covsb4b8,Covsb4b9,Covsb4cp,Sb5,Covsb5b6,Covsb5b7,Covsb5b
8,Covsb5b9,Covsb5cp,Sb6,Covsb6b7,Covsb6b8,Covsb6b9,Covsb6cp,Sb7,Covsb7b8,Covsb7b9,Cov
sb7cp,Sb8,Covsb8b9,Covsb8cp,Sb9,Covsb9cp,Scp),
  quant=~b19, alpha=0.05, type="asymp", n.asymp=20000)

# Total effect with confidence interval
total <- abtot+cp
total
set.seed(1234)
ci(mu=c(b1=a1, b2=a2, b3=a3, b4=a4, b5=a5, b6=a6, b7=a7, b8=a8, b9=a9, b10=b1, b11=b2,
b12=b3, b13=b4, b14=b5, b15=b6, b16=b7, b17=b8, b18=b9, b19=cp),
Sigma=c(Sa1,0,0,0,0,0,0,0,0,0,0,0,0,0,0,0,0,0,0,Sa2,0,0,0,0,0,0,0,0,0,0,0,0,0,0,0,0,0,Sa3,0,0,0,0,0,0,0
,0,0,0,0,0,0,0,0,0,Sa4,0,0,0,0,0,0,0,0,0,0,0,0,0,0,0,Sa5,
0,0,0,0,0,0,0,0,0,0,0,0,0,0,Sa6,0,0,0,0,0,0,0,0,0,0,0,0,0,Sa7,0,0,0,0,0,0,0,0,0,0,0,0,Sa8,0,0,0,0,0,0,0,0
,0,0,0,Sa9,0,0,0,0,0,0,0,0,0,0,Sb1,Covsb1b2,Covsb1b3,Covsb1b4,Covsb1b5,Covsb1b6,Covsb1b7,Co
vsb1b8,Covsb1b9,Covsb1cp,Sb2,Covsb2b3,Covsb2b4,Covsb2b5,Covsb2b6,Covsb2b7,Covsb2b8,Co
vsb2b9,Covsb2cp,Sb3,Covsb3b4,Covsb3b5,Covsb3b6,Covsb3b7,Covsb3b8,Covsb3b9,Covsb3cp,Sb
4,Covsb4b5,Covsb4b6,Covsb4b7,Covsb4b8,Covsb4b9,Covsb4cp,Sb5,Covsb5b6,Covsb5b7,Covsb5b
8,Covsb5b9,Covsb5cp,Sb6,Covsb6b7,Covsb6b8,Covsb6b9,Covsb6cp,Sb7,Covsb7b8,Covsb7b9,Cov
sb7cp,Sb8,Covsb8b9,Covsb8cp,Sb9,Covsb9cp,Scp),
```

quant=~b1*b10+b2*b11+b3*b12+b4*b13+b5*b14+ b6*b15+b7*b16+b8*b17+b9*b18+b19, alpha=0.05, type="asymp", n.asymp=20000)

1.2 Parallel-multiple mediator model: Micronutrient intake – Physical functioning

```
###VITAMIN B2 (PER SD) -PHYSICAL FUNCTIONING

### Estimate mediator model and save coefficients ###
# Mediator models
modela1 <- (lmer(Trp ~ VITB2_mg_day_SD + ENROLL_AGE + SEX + Creat + COM_NUMBER_CAT
+ P_CLIN_CHEM + WEEKS_SINCE_END_TREAT + STOMA + W0EDU_LEVEL + BMI + MVPA +
SMOKER_CURR2 + Energie_kcal_day + Alcohol_totaal_g_day + mox.mdursedge30min_h + (1
|PATIENTN), data=Dataset_Sumsc))
summary(modela1)
confint(modela1)

modela2 <- (lmer(Kyn ~ VITB2_mg_day_SD + ENROLL_AGE + SEX + Creat + COM_NUMBER_CAT
+ P_CLIN_CHEM + WEEKS_SINCE_END_TREAT + STOMA + W0EDU_LEVEL + BMI + MVPA +
SMOKER_CURR2 + Energie_kcal_day + Alcohol_totaal_g_day + mox.mdursedge30min_h + (1
|PATIENTN), data=Dataset_Sumsc))
summary(modela2)
confint(modela2)

modela3 <- (lmer(HK ~ VITB2_mg_day_SD + ENROLL_AGE + SEX + Creat + COM_NUMBER_CAT
+ P_CLIN_CHEM + WEEKS_SINCE_END_TREAT + STOMA + W0EDU_LEVEL + BMI + MVPA +
SMOKER_CURR2 + Energie_kcal_day + Alcohol_totaal_g_day + mox.mdursedge30min_h + (1
|PATIENTN), data=Dataset_Sumsc))
summary(modela3)
confint(modela3)

modela4 <- (lmer(KA ~ VITB2_mg_day_SD + ENROLL_AGE + SEX + Creat + COM_NUMBER_CAT
+ P_CLIN_CHEM + WEEKS_SINCE_END_TREAT + STOMA + W0EDU_LEVEL + BMI + MVPA +
SMOKER_CURR2 + Energie_kcal_day + Alcohol_totaal_g_day + mox.mdursedge30min_h + (1
|PATIENTN), data=Dataset_Sumsc))
summary(modela4)
confint(modela4)

modela5 <- (lmer(XA ~ VITB2_mg_day_SD + ENROLL_AGE + SEX + Creat + COM_NUMBER_CAT
+ P_CLIN_CHEM + WEEKS_SINCE_END_TREAT + STOMA + W0EDU_LEVEL + BMI + MVPA +
SMOKER_CURR2 + Energie_kcal_day + Alcohol_totaal_g_day + mox.mdursedge30min_h + (1
|PATIENTN), data=Dataset_Sumsc))
summary(modela5)
confint(modela5)

modela6 <- (lmer(AA ~ VITB2_mg_day_SD + ENROLL_AGE + SEX + Creat + COM_NUMBER_CAT
+ P_CLIN_CHEM + WEEKS_SINCE_END_TREAT + STOMA + W0EDU_LEVEL + BMI + MVPA +
SMOKER_CURR2 + Energie_kcal_day + Alcohol_totaal_g_day + mox.mdursedge30min_h + (1
|PATIENTN), data=Dataset_Sumsc))
summary(modela6)
confint(modela6)

modela7 <- (lmer(HAA ~ VITB2_mg_day_SD + ENROLL_AGE + SEX + Creat + COM_NUMBER_CAT
+ P_CLIN_CHEM + WEEKS_SINCE_END_TREAT + STOMA + W0EDU_LEVEL + BMI + MVPA +
SMOKER_CURR2 + Energie_kcal_day + Alcohol_totaal_g_day + mox.mdursedge30min_h + (1
|PATIENTN), data=Dataset_Sumsc))
summary(modela7)
confint(modela7)
```

```
modela8 <- (lmer(Pic ~ VITB2_mg_day_SD + ENROLL_AGE + SEX + Creat + COM_NUMBER_CAT
+ P_CLIN_CHEM + WEEKS_SINCE_END_TREAT + STOMA + W0EDU_LEVEL + BMI + MVPA +
SMOKER_CURR2 + Energie_kcal_day + Alcohol_totaal_g_day + mox.mdursedge30min_h + (1
|PATIENTN), data=Dataset_Sumsc))
summary(modela8)
confint(modela8)

modela9 <- (lmer(QA ~ VITB2_mg_day_SD + ENROLL_AGE + SEX + Creat + COM_NUMBER_CAT
+ P_CLIN_CHEM + WEEKS_SINCE_END_TREAT + STOMA + W0EDU_LEVEL + BMI + MVPA +
SMOKER_CURR2 + Energie_kcal_day + Alcohol_totaal_g_day + mox.mdursedge30min_h + (1
|PATIENTN), data=Dataset_Sumsc))
summary(modela9)
confint(modela9)

# Save a coefficients and variance
a1 <- modela1@beta[2]
Sa1 <- vcov(modela1) ["VITB2_mg_day_SD", "VITB2_mg_day_SD"]

a2 <- modela2@beta[2]
Sa2 <- vcov(modela2) ["VITB2_mg_day_SD", "VITB2_mg_day_SD"]

a3 <- modela3@beta[2]
Sa3 <- vcov(modela3) ["VITB2_mg_day_SD", "VITB2_mg_day_SD"]

a4 <- modela4@beta[2]
Sa4 <- vcov(modela4) ["VITB2_mg_day_SD", "VITB2_mg_day_SD"]

a5 <- modela5@beta[2]
Sa5 <- vcov(modela5) ["VITB2_mg_day_SD", "VITB2_mg_day_SD"]

a6 <- modela6@beta[2]
Sa6 <- vcov(modela6) ["VITB2_mg_day_SD", "VITB2_mg_day_SD"]

a7 <- modela7@beta[2]
Sa7 <- vcov(modela7) ["VITB2_mg_day_SD", "VITB2_mg_day_SD"]

a8 <- modela8@beta[2]
Sa8 <- vcov(modela8) ["VITB2_mg_day_SD", "VITB2_mg_day_SD"]

a9 <- modela9@beta[2]
Sa9 <- vcov(modela9) ["VITB2_mg_day_SD", "VITB2_mg_day_SD"]


### Estimate outcome model and save coefficients ###
# Outcome model
modelb <- (lmer(PF2 ~ VITB2_mg_day_SD + Trp + Kyn + HK + KA + XA + AA + HAA + Pic + QA +
ENROLL_AGE + SEX + Creat + COM_NUMBER_CAT + P_CLIN_CHEM +
WEEKS_SINCE_END_TREAT + STOMA + W0EDU_LEVEL + BMI + MVPA + SMOKER_CURR2 +
Energie_kcal_day + Alcohol_totaal_g_day + mox.mdursedge30min_h + (1 |PATIENTN),
data=Dataset_Sumsc))
summary(modelb)
confint(modelb)

# Save c' coefficient and variance
cp <- modelb@beta[2]
```

```
Scp <- vcov(modelb) ["VITB2_mg_day_SD", "VITB2_mg_day_SD"]

# Save b coefficient and variance
b1 <- modelb@beta[3]
Sb1 <- vcov(modelb) ["Trp", "Trp"]
Covsb1b2 <- vcov(modelb) ["Trp", "Kyn"]
Covsb1b3 <- vcov(modelb) ["Trp", "HK"]
Covsb1b4 <- vcov(modelb) ["Trp", "KA"]
Covsb1b5 <- vcov(modelb) ["Trp", "XA"]
Covsb1b6 <- vcov(modelb) ["Trp", "AA"]
Covsb1b7 <- vcov(modelb) ["Trp", "HAA"]
Covsb1b8 <- vcov(modelb) ["Trp", "Pic"]
Covsb1b9 <- vcov(modelb) ["Trp", "QA"]
Covsb1cp <- vcov(modelb) ["Trp", "VITB2_mg_day_SD"]

b2 <- modelb@beta[4]
Sb2 <- vcov(modelb) ["Kyn", "Kyn"]
Covsb2b3 <- vcov(modelb) ["Kyn", "HK"]
Covsb2b4 <- vcov(modelb) ["Kyn", "KA"]
Covsb2b5 <- vcov(modelb) ["Kyn", "XA"]
Covsb2b6 <- vcov(modelb) ["Kyn", "AA"]
Covsb2b7 <- vcov(modelb) ["Kyn", "HAA"]
Covsb2b8 <- vcov(modelb) ["Kyn", "Pic"]
Covsb2b9 <- vcov(modelb) ["Kyn", "QA"]
Covsb2cp <- vcov(modelb) ["Kyn", "VITB2_mg_day_SD"]

b3 <- modelb@beta[5]
Sb3 <- vcov(modelb) ["HK", "HK"]
Covsb3b4 <- vcov(modelb) ["HK", "KA"]
Covsb3b5 <- vcov(modelb) ["HK", "XA"]
Covsb3b6 <- vcov(modelb) ["HK", "AA"]
Covsb3b7 <- vcov(modelb) ["HK", "HAA"]
Covsb3b8 <- vcov(modelb) ["HK", "Pic"]
Covsb3b9 <- vcov(modelb) ["HK", "QA"]
Covsb3cp <- vcov(modelb) ["HK", "VITB2_mg_day_SD"]

b4 <- modelb@beta[6]
Sb4 <- vcov(modelb) ["KA", "KA"]
Covsb4b5 <- vcov(modelb) ["KA", "XA"]
Covsb4b6 <- vcov(modelb) ["KA", "AA"]
Covsb4b7 <- vcov(modelb) ["KA", "HAA"]
Covsb4b8 <- vcov(modelb) ["KA", "Pic"]
Covsb4b9 <- vcov(modelb) ["KA", "QA"]
Covsb4cp <- vcov(modelb) ["KA", "VITB2_mg_day_SD"]

b5 <- modelb@beta[7]
Sb5 <- vcov(modelb) ["XA", "XA"]
Covsb5b6 <- vcov(modelb) ["XA", "AA"]
Covsb5b7 <- vcov(modelb) ["XA", "HAA"]
Covsb5b8 <- vcov(modelb) ["XA", "Pic"]
Covsb5b9 <- vcov(modelb) ["XA", "QA"]
Covsb5cp <- vcov(modelb) ["XA", "VITB2_mg_day_SD"]

b6 <- modelb@beta[8]
Sb6 <- vcov(modelb) ["AA", "AA"]
Covsb6b7 <- vcov(modelb) ["AA", "HAA"]
```

```
Covsb6b8 <- vcov(modelb) ["AA", "Pic"]
Covsb6b9 <- vcov(modelb) ["AA", "QA"]
Covsb6cp <- vcov(modelb) ["AA", "VITB2_mg_day_SD"]

b7 <- modelb@beta[9]
Sb7 <- vcov(modelb) ["HAA", "HAA"]
Covsb7b8 <- vcov(modelb) ["HAA", "Pic"]
Covsb7b9 <- vcov(modelb) ["HAA", "QA"]
Covsb7cp <- vcov(modelb) ["HAA", "VITB2_mg_day_SD"]

b8 <- modelb@beta[10]
Sb8 <- vcov(modelb) ["Pic", "Pic"]
Covsb8b9 <- vcov(modelb) ["Pic", "QA"]
Covsb8cp <- vcov(modelb) ["Pic", "VITB2_mg_day_SD"]

b9 <- modelb@beta[11]
Sb9 <- vcov(modelb) ["QA", "QA"]
Covsb9cp <- vcov(modelb) ["QA", "VITB2_mg_day_SD"]

### Compute mediation effects with confidence intervals ###
# Indirect effect with Monte Carlo confidence interval
ab1 <- a1*b1
ab1
set.seed(1234)
ci(mu=c(b1=a1, b2=a2, b3=a3, b4=a4, b5=a5, b6=a6, b7=a7, b8=a8, b9=a9, b10=b1, b11=b2,
b12=b3, b13=b4, b14=b5, b15=b6, b16=b7, b17=b8, b18=b9, b19=cp),
Sigma=c(Sa1,0,0,0,0,0,0,0,0,0,0,0,0,0,0,0,0,0,0,Sa2,0,0,0,0,0,0,0,0,0,0,0,0,0,0,0,0,0,Sa3,0,0,0,0,0,0,0
,0,0,0,0,0,0,0,0,0,Sa4,0,0,0,0,0,0,0,0,0,0,0,0,0,0,0,Sa5,
0,0,0,0,0,0,0,0,0,0,0,0,0,0,Sa6,0,0,0,0,0,0,0,0,0,0,0,0,0,Sa7,0,0,0,0,0,0,0,0,0,0,0,0,Sa8,0,0,0,0,0,0,0,0
,0,0,0,Sa9,0,0,0,0,0,0,0,0,0,0,Sb1,Covsb1b2,Covsb1b3,Covsb1b4,Covsb1b5,Covsb1b6,Covsb1b7,Co
vsb1b8,Covsb1b9,Covsb1cp,Sb2,Covsb2b3,Covsb2b4,Covsb2b5,Covsb2b6,Covsb2b7,Covsb2b8,Co
vsb2b9,Covsb2cp,Sb3,Covsb3b4,Covsb3b5,Covsb3b6,Covsb3b7,Covsb3b8,Covsb3b9,Covsb3cp,Sb
4,Covsb4b5,Covsb4b6,Covsb4b7,Covsb4b8,Covsb4b9,Covsb4cp,Sb5,Covsb5b6,Covsb5b7,Covsb5b
8,Covsb5b9,Covsb5cp,Sb6,Covsb6b7,Covsb6b8,Covsb6b9,Covsb6cp,Sb7,Covsb7b8,Covsb7b9,Cov
sb7cp,Sb8,Covsb8b9,Covsb8cp,Sb9,Covsb9cp,Scp),
  quant=~b1*b10, alpha=0.05, type="MC", n.mc=20000)

ab2 <- a2*b2
ab2
set.seed(1234)
ci(mu=c(b1=a1, b2=a2, b3=a3, b4=a4, b5=a5, b6=a6, b7=a7, b8=a8, b9=a9, b10=b1, b11=b2,
b12=b3, b13=b4, b14=b5, b15=b6, b16=b7, b17=b8, b18=b9, b19=cp),
Sigma=c(Sa1,0,0,0,0,0,0,0,0,0,0,0,0,0,0,0,0,0,0,Sa2,0,0,0,0,0,0,0,0,0,0,0,0,0,0,0,0,0,Sa3,0,0,0,0,0,0,0
,0,0,0,0,0,0,0,0,0,Sa4,0,0,0,0,0,0,0,0,0,0,0,0,0,0,0,Sa5,
0,0,0,0,0,0,0,0,0,0,0,0,0,0,Sa6,0,0,0,0,0,0,0,0,0,0,0,0,0,Sa7,0,0,0,0,0,0,0,0,0,0,0,0,Sa8,0,0,0,0,0,0,0,0
,0,0,0,Sa9,0,0,0,0,0,0,0,0,0,0,Sb1,Covsb1b2,Covsb1b3,Covsb1b4,Covsb1b5,Covsb1b6,Covsb1b7,Co
vsb1b8,Covsb1b9,Covsb1cp,Sb2,Covsb2b3,Covsb2b4,Covsb2b5,Covsb2b6,Covsb2b7,Covsb2b8,Co
vsb2b9,Covsb2cp,Sb3,Covsb3b4,Covsb3b5,Covsb3b6,Covsb3b7,Covsb3b8,Covsb3b9,Covsb3cp,Sb
4,Covsb4b5,Covsb4b6,Covsb4b7,Covsb4b8,Covsb4b9,Covsb4cp,Sb5,Covsb5b6,Covsb5b7,Covsb5b
8,Covsb5b9,Covsb5cp,Sb6,Covsb6b7,Covsb6b8,Covsb6b9,Covsb6cp,Sb7,Covsb7b8,Covsb7b9,Cov
sb7cp,Sb8,Covsb8b9,Covsb8cp,Sb9,Covsb9cp,Scp),
  quant=~b2*b11, alpha=0.05, type="MC", n.mc=20000)

ab3 <- a3*b3
ab3
set.seed(1234)
```

```
ci(mu=c(b1=a1, b2=a2, b3=a3, b4=a4, b5=a5, b6=a6, b7=a7, b8=a8, b9=a9, b10=b1, b11=b2,
b12=b3, b13=b4, b14=b5, b15=b6, b16=b7, b17=b8, b18=b9, b19=cp),
Sigma=c(Sa1,0,0,0,0,0,0,0,0,0,0,0,0,0,0,0,0,0,0,Sa2,0,0,0,0,0,0,0,0,0,0,0,0,0,0,0,0,0,Sa3,0,0,0,0,0,0,0
,0,0,0,0,0,0,0,0,0,Sa4,0,0,0,0,0,0,0,0,0,0,0,0,0,0,0,Sa5,
0,0,0,0,0,0,0,0,0,0,0,0,0,0,Sa6,0,0,0,0,0,0,0,0,0,0,0,0,0,Sa7,0,0,0,0,0,0,0,0,0,0,0,0,Sa8,0,0,0,0,0,0,0,0
,0,0,0,Sa9,0,0,0,0,0,0,0,0,0,0,Sb1,Covsb1b2,Covsb1b3,Covsb1b4,Covsb1b5,Covsb1b6,Covsb1b7,Co
vsb1b8,Covsb1b9,Covsb1cp,Sb2,Covsb2b3,Covsb2b4,Covsb2b5,Covsb2b6,Covsb2b7,Covsb2b8,Co
vsb2b9,Covsb2cp,Sb3,Covsb3b4,Covsb3b5,Covsb3b6,Covsb3b7,Covsb3b8,Covsb3b9,Covsb3cp,Sb
4,Covsb4b5,Covsb4b6,Covsb4b7,Covsb4b8,Covsb4b9,Covsb4cp,Sb5,Covsb5b6,Covsb5b7,Covsb5b
8,Covsb5b9,Covsb5cp,Sb6,Covsb6b7,Covsb6b8,Covsb6b9,Covsb6cp,Sb7,Covsb7b8,Covsb7b9,Cov
sb7cp,Sb8,Covsb8b9,Covsb8cp,Sb9,Covsb9cp,Scp),
  quant=~b3*b12, alpha=0.05, type="MC", n.mc=20000)

ab4 <- a4*b4
ab4
set.seed(1234)
ci(mu=c(b1=a1, b2=a2, b3=a3, b4=a4, b5=a5, b6=a6, b7=a7, b8=a8, b9=a9, b10=b1, b11=b2,
b12=b3, b13=b4, b14=b5, b15=b6, b16=b7, b17=b8, b18=b9, b19=cp),
Sigma=c(Sa1,0,0,0,0,0,0,0,0,0,0,0,0,0,0,0,0,0,0,Sa2,0,0,0,0,0,0,0,0,0,0,0,0,0,0,0,0,0,Sa3,0,0,0,0,0,0,0
,0,0,0,0,0,0,0,0,0,Sa4,0,0,0,0,0,0,0,0,0,0,0,0,0,0,0,Sa5,
0,0,0,0,0,0,0,0,0,0,0,0,0,0,Sa6,0,0,0,0,0,0,0,0,0,0,0,0,0,Sa7,0,0,0,0,0,0,0,0,0,0,0,0,Sa8,0,0,0,0,0,0,0,0
,0,0,0,Sa9,0,0,0,0,0,0,0,0,0,0,Sb1,Covsb1b2,Covsb1b3,Covsb1b4,Covsb1b5,Covsb1b6,Covsb1b7,Co
vsb1b8,Covsb1b9,Covsb1cp,Sb2,Covsb2b3,Covsb2b4,Covsb2b5,Covsb2b6,Covsb2b7,Covsb2b8,Co
vsb2b9,Covsb2cp,Sb3,Covsb3b4,Covsb3b5,Covsb3b6,Covsb3b7,Covsb3b8,Covsb3b9,Covsb3cp,Sb
4,Covsb4b5,Covsb4b6,Covsb4b7,Covsb4b8,Covsb4b9,Covsb4cp,Sb5,Covsb5b6,Covsb5b7,Covsb5b
8,Covsb5b9,Covsb5cp,Sb6,Covsb6b7,Covsb6b8,Covsb6b9,Covsb6cp,Sb7,Covsb7b8,Covsb7b9,Cov
sb7cp,Sb8,Covsb8b9,Covsb8cp,Sb9,Covsb9cp,Scp),
  quant=~b4*b13, alpha=0.05, type="MC", n.mc=20000)

ab5 <- a5*b5
ab5
set.seed(1234)
ci(mu=c(b1=a1, b2=a2, b3=a3, b4=a4, b5=a5, b6=a6, b7=a7, b8=a8, b9=a9, b10=b1, b11=b2,
b12=b3, b13=b4, b14=b5, b15=b6, b16=b7, b17=b8, b18=b9, b19=cp),
Sigma=c(Sa1,0,0,0,0,0,0,0,0,0,0,0,0,0,0,0,0,0,0,Sa2,0,0,0,0,0,0,0,0,0,0,0,0,0,0,0,0,0,Sa3,0,0,0,0,0,0,0
,0,0,0,0,0,0,0,0,0,Sa4,0,0,0,0,0,0,0,0,0,0,0,0,0,0,0,Sa5,
0,0,0,0,0,0,0,0,0,0,0,0,0,0,Sa6,0,0,0,0,0,0,0,0,0,0,0,0,0,Sa7,0,0,0,0,0,0,0,0,0,0,0,0,Sa8,0,0,0,0,0,0,0,0
,0,0,0,Sa9,0,0,0,0,0,0,0,0,0,0,Sb1,Covsb1b2,Covsb1b3,Covsb1b4,Covsb1b5,Covsb1b6,Covsb1b7,Co
vsb1b8,Covsb1b9,Covsb1cp,Sb2,Covsb2b3,Covsb2b4,Covsb2b5,Covsb2b6,Covsb2b7,Covsb2b8,Co
vsb2b9,Covsb2cp,Sb3,Covsb3b4,Covsb3b5,Covsb3b6,Covsb3b7,Covsb3b8,Covsb3b9,Covsb3cp,Sb
4,Covsb4b5,Covsb4b6,Covsb4b7,Covsb4b8,Covsb4b9,Covsb4cp,Sb5,Covsb5b6,Covsb5b7,Covsb5b
8,Covsb5b9,Covsb5cp,Sb6,Covsb6b7,Covsb6b8,Covsb6b9,Covsb6cp,Sb7,Covsb7b8,Covsb7b9,Cov
sb7cp,Sb8,Covsb8b9,Covsb8cp,Sb9,Covsb9cp,Scp),
  quant=~b5*b14, alpha=0.05, type="MC", n.mc=20000)

ab6 <- a6*b6
ab6
set.seed(1234)
ci(mu=c(b1=a1, b2=a2, b3=a3, b4=a4, b5=a5, b6=a6, b7=a7, b8=a8, b9=a9, b10=b1, b11=b2,
b12=b3, b13=b4, b14=b5, b15=b6, b16=b7, b17=b8, b18=b9, b19=cp),
Sigma=c(Sa1,0,0,0,0,0,0,0,0,0,0,0,0,0,0,0,0,0,0,Sa2,0,0,0,0,0,0,0,0,0,0,0,0,0,0,0,0,0,Sa3,0,0,0,0,0,0,0
,0,0,0,0,0,0,0,0,0,Sa4,0,0,0,0,0,0,0,0,0,0,0,0,0,0,0,Sa5,
0,0,0,0,0,0,0,0,0,0,0,0,0,0,Sa6,0,0,0,0,0,0,0,0,0,0,0,0,0,Sa7,0,0,0,0,0,0,0,0,0,0,0,0,Sa8,0,0,0,0,0,0,0,0
,0,0,0,Sa9,0,0,0,0,0,0,0,0,0,0,Sb1,Covsb1b2,Covsb1b3,Covsb1b4,Covsb1b5,Covsb1b6,Covsb1b7,Co
vsb1b8,Covsb1b9,Covsb1cp,Sb2,Covsb2b3,Covsb2b4,Covsb2b5,Covsb2b6,Covsb2b7,Covsb2b8,Co
vsb2b9,Covsb2cp,Sb3,Covsb3b4,Covsb3b5,Covsb3b6,Covsb3b7,Covsb3b8,Covsb3b9,Covsb3cp,Sb
```

```
4,Covsb4b5,Covsb4b6,Covsb4b7,Covsb4b8,Covsb4b9,Covsb4cp,Sb5,Covsb5b6,Covsb5b7,Covsb5b
8,Covsb5b9,Covsb5cp,Sb6,Covsb6b7,Covsb6b8,Covsb6b9,Covsb6cp,Sb7,Covsb7b8,Covsb7b9,Cov
sb7cp,Sb8,Covsb8b9,Covsb8cp,Sb9,Covsb9cp,Scp),
  quant=~b6*b15, alpha=0.05, type="MC", n.mc=20000)

ab7 <- a7*b7
ab7
set.seed(1234)
ci(mu=c(b1=a1, b2=a2, b3=a3, b4=a4, b5=a5, b6=a6, b7=a7, b8=a8, b9=a9, b10=b1, b11=b2,
b12=b3, b13=b4, b14=b5, b15=b6, b16=b7, b17=b8, b18=b9, b19=cp),
Sigma=c(Sa1,0,0,0,0,0,0,0,0,0,0,0,0,0,0,0,0,0,0,Sa2,0,0,0,0,0,0,0,0,0,0,0,0,0,0,0,0,0,Sa3,0,0,0,0,0,0,0
,0,0,0,0,0,0,0,0,0,Sa4,0,0,0,0,0,0,0,0,0,0,0,0,0,0,0,Sa5,
0,0,0,0,0,0,0,0,0,0,0,0,0,0,Sa6,0,0,0,0,0,0,0,0,0,0,0,0,0,Sa7,0,0,0,0,0,0,0,0,0,0,0,0,Sa8,0,0,0,0,0,0,0,0
,0,0,0,Sa9,0,0,0,0,0,0,0,0,0,0,Sb1,Covsb1b2,Covsb1b3,Covsb1b4,Covsb1b5,Covsb1b6,Covsb1b7,Co
vsb1b8,Covsb1b9,Covsb1cp,Sb2,Covsb2b3,Covsb2b4,Covsb2b5,Covsb2b6,Covsb2b7,Covsb2b8,Co
vsb2b9,Covsb2cp,Sb3,Covsb3b4,Covsb3b5,Covsb3b6,Covsb3b7,Covsb3b8,Covsb3b9,Covsb3cp,Sb
4,Covsb4b5,Covsb4b6,Covsb4b7,Covsb4b8,Covsb4b9,Covsb4cp,Sb5,Covsb5b6,Covsb5b7,Covsb5b
8,Covsb5b9,Covsb5cp,Sb6,Covsb6b7,Covsb6b8,Covsb6b9,Covsb6cp,Sb7,Covsb7b8,Covsb7b9,Cov
sb7cp,Sb8,Covsb8b9,Covsb8cp,Sb9,Covsb9cp,Scp),
  quant=~b7*b16, alpha=0.05, type="MC", n.mc=20000)

ab8 <- a8*b8
ab8
set.seed(1234)
ci(mu=c(b1=a1, b2=a2, b3=a3, b4=a4, b5=a5, b6=a6, b7=a7, b8=a8, b9=a9, b10=b1, b11=b2,
b12=b3, b13=b4, b14=b5, b15=b6, b16=b7, b17=b8, b18=b9, b19=cp),
Sigma=c(Sa1,0,0,0,0,0,0,0,0,0,0,0,0,0,0,0,0,0,0,Sa2,0,0,0,0,0,0,0,0,0,0,0,0,0,0,0,0,0,Sa3,0,0,0,0,0,0,0
,0,0,0,0,0,0,0,0,0,Sa4,0,0,0,0,0,0,0,0,0,0,0,0,0,0,0,Sa5,
0,0,0,0,0,0,0,0,0,0,0,0,0,0,Sa6,0,0,0,0,0,0,0,0,0,0,0,0,0,Sa7,0,0,0,0,0,0,0,0,0,0,0,0,Sa8,0,0,0,0,0,0,0,0
,0,0,0,Sa9,0,0,0,0,0,0,0,0,0,0,Sb1,Covsb1b2,Covsb1b3,Covsb1b4,Covsb1b5,Covsb1b6,Covsb1b7,Co
vsb1b8,Covsb1b9,Covsb1cp,Sb2,Covsb2b3,Covsb2b4,Covsb2b5,Covsb2b6,Covsb2b7,Covsb2b8,Co
vsb2b9,Covsb2cp,Sb3,Covsb3b4,Covsb3b5,Covsb3b6,Covsb3b7,Covsb3b8,Covsb3b9,Covsb3cp,Sb
4,Covsb4b5,Covsb4b6,Covsb4b7,Covsb4b8,Covsb4b9,Covsb4cp,Sb5,Covsb5b6,Covsb5b7,Covsb5b
8,Covsb5b9,Covsb5cp,Sb6,Covsb6b7,Covsb6b8,Covsb6b9,Covsb6cp,Sb7,Covsb7b8,Covsb7b9,Cov
sb7cp,Sb8,Covsb8b9,Covsb8cp,Sb9,Covsb9cp,Scp),
  quant=~b8*b17, alpha=0.05, type="MC", n.mc=20000)

ab9 <- a9*b9
ab9
set.seed(1234)
ci(mu=c(b1=a1, b2=a2, b3=a3, b4=a4, b5=a5, b6=a6, b7=a7, b8=a8, b9=a9, b10=b1, b11=b2,
b12=b3, b13=b4, b14=b5, b15=b6, b16=b7, b17=b8, b18=b9, b19=cp),
Sigma=c(Sa1,0,0,0,0,0,0,0,0,0,0,0,0,0,0,0,0,0,0,Sa2,0,0,0,0,0,0,0,0,0,0,0,0,0,0,0,0,0,Sa3,0,0,0,0,0,0,0
,0,0,0,0,0,0,0,0,0,Sa4,0,0,0,0,0,0,0,0,0,0,0,0,0,0,0,Sa5,
0,0,0,0,0,0,0,0,0,0,0,0,0,0,Sa6,0,0,0,0,0,0,0,0,0,0,0,0,0,Sa7,0,0,0,0,0,0,0,0,0,0,0,0,Sa8,0,0,0,0,0,0,0,0
,0,0,0,Sa9,0,0,0,0,0,0,0,0,0,0,Sb1,Covsb1b2,Covsb1b3,Covsb1b4,Covsb1b5,Covsb1b6,Covsb1b7,Co
vsb1b8,Covsb1b9,Covsb1cp,Sb2,Covsb2b3,Covsb2b4,Covsb2b5,Covsb2b6,Covsb2b7,Covsb2b8,Co
vsb2b9,Covsb2cp,Sb3,Covsb3b4,Covsb3b5,Covsb3b6,Covsb3b7,Covsb3b8,Covsb3b9,Covsb3cp,Sb
4,Covsb4b5,Covsb4b6,Covsb4b7,Covsb4b8,Covsb4b9,Covsb4cp,Sb5,Covsb5b6,Covsb5b7,Covsb5b
8,Covsb5b9,Covsb5cp,Sb6,Covsb6b7,Covsb6b8,Covsb6b9,Covsb6cp,Sb7,Covsb7b8,Covsb7b9,Cov
sb7cp,Sb8,Covsb8b9,Covsb8cp,Sb9,Covsb9cp,Scp),
  quant=~b9*b18, alpha=0.05, type="MC", n.mc=20000)

abtot <- ab1 + ab2 + ab3 + ab4 + ab5 + ab6 + ab7 + ab8 + ab9
abtot
set.seed(1234)
```

```
ci(mu=c(b1=a1, b2=a2, b3=a3, b4=a4, b5=a5, b6=a6, b7=a7, b8=a8, b9=a9, b10=b1, b11=b2,
b12=b3, b13=b4, b14=b5, b15=b6, b16=b7, b17=b8, b18=b9, b19=cp),
Sigma=c(Sa1,0,0,0,0,0,0,0,0,0,0,0,0,0,0,0,0,0,0,Sa2,0,0,0,0,0,0,0,0,0,0,0,0,0,0,0,0,0,0,Sa3,0,0,0,0,0,0,0
,0,0,0,0,0,0,0,0,0,Sa4,0,0,0,0,0,0,0,0,0,0,0,0,0,0,0,Sa5,
0,0,0,0,0,0,0,0,0,0,0,0,0,0,Sa6,0,0,0,0,0,0,0,0,0,0,0,0,0,Sa7,0,0,0,0,0,0,0,0,0,0,0,0,Sa8,0,0,0,0,0,0,0,0
,0,0,0,Sa9,0,0,0,0,0,0,0,0,0,0,Sb1,Covsb1b2,Covsb1b3,Covsb1b4,Covsb1b5,Covsb1b6,Covsb1b7,Co
vsb1b8,Covsb1b9,Covsb1cp,Sb2,Covsb2b3,Covsb2b4,Covsb2b5,Covsb2b6,Covsb2b7,Covsb2b8,Co
vsb2b9,Covsb2cp,Sb3,Covsb3b4,Covsb3b5,Covsb3b6,Covsb3b7,Covsb3b8,Covsb3b9,Covsb3cp,Sb
4,Covsb4b5,Covsb4b6,Covsb4b7,Covsb4b8,Covsb4b9,Covsb4cp,Sb5,Covsb5b6,Covsb5b7,Covsb5b
8,Covsb5b9,Covsb5cp,Sb6,Covsb6b7,Covsb6b8,Covsb6b9,Covsb6cp,Sb7,Covsb7b8,Covsb7b9,Cov
sb7cp,Sb8,Covsb8b9,Covsb8cp,Sb9,Covsb9cp,Scp),
  quant=~b1*b10+b2*b11+b3*b12+b4*b13+b5*b14+ b6*b15+b7*b16+b8*b17+b9*b18, alpha=0.05,
type="MC", n.mc=20000)

# Direct effect with confidence interval
direct <- cp
direct
set.seed(1234)
ci(mu=c(b1=a1, b2=a2, b3=a3, b4=a4, b5=a5, b6=a6, b7=a7, b8=a8, b9=a9, b10=b1, b11=b2,
b12=b3, b13=b4, b14=b5, b15=b6, b16=b7, b17=b8, b18=b9, b19=cp),
Sigma=c(Sa1,0,0,0,0,0,0,0,0,0,0,0,0,0,0,0,0,0,0,Sa2,0,0,0,0,0,0,0,0,0,0,0,0,0,0,0,0,0,0,Sa3,0,0,0,0,0,0,0
,0,0,0,0,0,0,0,0,0,Sa4,0,0,0,0,0,0,0,0,0,0,0,0,0,0,0,Sa5,
0,0,0,0,0,0,0,0,0,0,0,0,0,0,Sa6,0,0,0,0,0,0,0,0,0,0,0,0,0,Sa7,0,0,0,0,0,0,0,0,0,0,0,0,Sa8,0,0,0,0,0,0,0,0
,0,0,0,Sa9,0,0,0,0,0,0,0,0,0,0,Sb1,Covsb1b2,Covsb1b3,Covsb1b4,Covsb1b5,Covsb1b6,Covsb1b7,Co
vsb1b8,Covsb1b9,Covsb1cp,Sb2,Covsb2b3,Covsb2b4,Covsb2b5,Covsb2b6,Covsb2b7,Covsb2b8,Co
vsb2b9,Covsb2cp,Sb3,Covsb3b4,Covsb3b5,Covsb3b6,Covsb3b7,Covsb3b8,Covsb3b9,Covsb3cp,Sb
4,Covsb4b5,Covsb4b6,Covsb4b7,Covsb4b8,Covsb4b9,Covsb4cp,Sb5,Covsb5b6,Covsb5b7,Covsb5b
8,Covsb5b9,Covsb5cp,Sb6,Covsb6b7,Covsb6b8,Covsb6b9,Covsb6cp,Sb7,Covsb7b8,Covsb7b9,Cov
sb7cp,Sb8,Covsb8b9,Covsb8cp,Sb9,Covsb9cp,Scp),
  quant=~b19, alpha=0.05, type="asymp", n.asymp=20000)

# Total effect with confidence interval
total <- abtot+cp
total
set.seed(1234)
ci(mu=c(b1=a1, b2=a2, b3=a3, b4=a4, b5=a5, b6=a6, b7=a7, b8=a8, b9=a9, b10=b1, b11=b2,
b12=b3, b13=b4, b14=b5, b15=b6, b16=b7, b17=b8, b18=b9, b19=cp),
Sigma=c(Sa1,0,0,0,0,0,0,0,0,0,0,0,0,0,0,0,0,0,0,Sa2,0,0,0,0,0,0,0,0,0,0,0,0,0,0,0,0,0,0,Sa3,0,0,0,0,0,0,0
,0,0,0,0,0,0,0,0,0,Sa4,0,0,0,0,0,0,0,0,0,0,0,0,0,0,0,Sa5,
0,0,0,0,0,0,0,0,0,0,0,0,0,0,Sa6,0,0,0,0,0,0,0,0,0,0,0,0,0,Sa7,0,0,0,0,0,0,0,0,0,0,0,0,Sa8,0,0,0,0,0,0,0,0
,0,0,0,Sa9,0,0,0,0,0,0,0,0,0,0,Sb1,Covsb1b2,Covsb1b3,Covsb1b4,Covsb1b5,Covsb1b6,Covsb1b7,Co
vsb1b8,Covsb1b9,Covsb1cp,Sb2,Covsb2b3,Covsb2b4,Covsb2b5,Covsb2b6,Covsb2b7,Covsb2b8,Co
vsb2b9,Covsb2cp,Sb3,Covsb3b4,Covsb3b5,Covsb3b6,Covsb3b7,Covsb3b8,Covsb3b9,Covsb3cp,Sb
4,Covsb4b5,Covsb4b6,Covsb4b7,Covsb4b8,Covsb4b9,Covsb4cp,Sb5,Covsb5b6,Covsb5b7,Covsb5b
8,Covsb5b9,Covsb5cp,Sb6,Covsb6b7,Covsb6b8,Covsb6b9,Covsb6cp,Sb7,Covsb7b8,Covsb7b9,Cov
sb7cp,Sb8,Covsb8b9,Covsb8cp,Sb9,Covsb9cp,Scp),
  quant=~b1*b10+b2*b11+b3*b12+b4*b13+b5*b14+ b6*b15+b7*b16+b8*b17+b9*b18+b19,
alpha=0.05, type="asymp", n.asymp=20000)
```

1.3 Parallel-multiple mediator model: Dietary pattern – Physical functioning

```
###DHD-PHYSICAL FUNCTIONING

### Estimate mediator model and save coefficients ###
# Mediator models
modela1 <- (lmer(Trp ~ DHD_TOTAL_SD + ENROLL_AGE + SEX + Creat + COM_NUMBER_CAT +
P_CLIN_CHEM + WEEKS_SINCE_END_TREAT + STOMA + W0EDU_LEVEL + BMI + MVPA +
SMOKER_CURR2 + Energie_kcal_day + mox.mdursedge30min_h + (1 |PATIENTN),
data=Dataset_Sumsc))
summary(modela1)
confint(modela1)

modela2 <- (lmer(Kyn ~ DHD_TOTAL_SD + ENROLL_AGE + SEX + Creat + COM_NUMBER_CAT +
P_CLIN_CHEM + WEEKS_SINCE_END_TREAT + STOMA + W0EDU_LEVEL + BMI + MVPA +
SMOKER_CURR2 + Energie_kcal_day + mox.mdursedge30min_h + (1 |PATIENTN),
data=Dataset_Sumsc))
summary(modela2)
confint(modela2)

modela3 <- (lmer(HK ~ DHD_TOTAL_SD + ENROLL_AGE + SEX + Creat + COM_NUMBER_CAT +
P_CLIN_CHEM + WEEKS_SINCE_END_TREAT + STOMA + W0EDU_LEVEL + BMI + MVPA +
SMOKER_CURR2 + Energie_kcal_day + mox.mdursedge30min_h + (1 |PATIENTN),
data=Dataset_Sumsc))
summary(modela3)
confint(modela3)

modela4 <- (lmer(KA ~ DHD_TOTAL_SD + ENROLL_AGE + SEX + Creat + COM_NUMBER_CAT +
P_CLIN_CHEM + WEEKS_SINCE_END_TREAT + STOMA + W0EDU_LEVEL + BMI + MVPA +
SMOKER_CURR2 + Energie_kcal_day + mox.mdursedge30min_h + (1 |PATIENTN),
data=Dataset_Sumsc))
summary(modela4)
confint(modela4)

modela5 <- (lmer(XA ~ DHD_TOTAL_SD + ENROLL_AGE + SEX + Creat + COM_NUMBER_CAT +
P_CLIN_CHEM + WEEKS_SINCE_END_TREAT + STOMA + W0EDU_LEVEL + BMI + MVPA +
SMOKER_CURR2 + Energie_kcal_day + mox.mdursedge30min_h + (1 |PATIENTN),
data=Dataset_Sumsc))
summary(modela5)
confint(modela5)

modela6 <- (lmer(AA ~ DHD_TOTAL_SD + ENROLL_AGE + SEX + Creat + COM_NUMBER_CAT +
P_CLIN_CHEM + WEEKS_SINCE_END_TREAT + STOMA + W0EDU_LEVEL + BMI + MVPA +
SMOKER_CURR2 + Energie_kcal_day + mox.mdursedge30min_h + (1 |PATIENTN),
data=Dataset_Sumsc))
summary(modela6)
confint(modela6)

modela7 <- (lmer(HAA ~ DHD_TOTAL_SD + ENROLL_AGE + SEX + Creat + COM_NUMBER_CAT +
P_CLIN_CHEM + WEEKS_SINCE_END_TREAT + STOMA + W0EDU_LEVEL + BMI + MVPA +
SMOKER_CURR2 + Energie_kcal_day + mox.mdursedge30min_h + (1 |PATIENTN),
data=Dataset_Sumsc))
summary(modela7)
confint(modela7)
```

```
modela8 <- (lmer(Pic ~ DHD_TOTAL_SD + ENROLL_AGE + SEX + Creat + COM_NUMBER_CAT +
P_CLIN_CHEM + WEEKS_SINCE_END_TREAT + STOMA + W0EDU_LEVEL + BMI + MVPA +
SMOKER_CURR2 + Energie_kcal_day + mox.mdursedge30min_h + (1 |PATIENTN),
data=Dataset_Sumsc))
summary(modela8)
confint(modela8)

modela9 <- (lmer(QA ~ DHD_TOTAL_SD + ENROLL_AGE + SEX + Creat + COM_NUMBER_CAT +
P_CLIN_CHEM + WEEKS_SINCE_END_TREAT + STOMA + W0EDU_LEVEL + BMI + MVPA +
SMOKER_CURR2 + Energie_kcal_day + mox.mdursedge30min_h + (1 |PATIENTN),
data=Dataset_Sumsc))
summary(modela9)
confint(modela9)

# Save a coefficients and variance
a1 <- modela1@beta[2]
Sa1 <- vcov(modela1) ["DHD_TOTAL_SD", "DHD_TOTAL_SD"]

a2 <- modela2@beta[2]
Sa2 <- vcov(modela2) ["DHD_TOTAL_SD", "DHD_TOTAL_SD"]

a3 <- modela3@beta[2]
Sa3 <- vcov(modela3) ["DHD_TOTAL_SD", "DHD_TOTAL_SD"]

a4 <- modela4@beta[2]
Sa4 <- vcov(modela4) ["DHD_TOTAL_SD", "DHD_TOTAL_SD"]

a5 <- modela5@beta[2]
Sa5 <- vcov(modela5) ["DHD_TOTAL_SD", "DHD_TOTAL_SD"]

a6 <- modela6@beta[2]
Sa6 <- vcov(modela6) ["DHD_TOTAL_SD", "DHD_TOTAL_SD"]

a7 <- modela7@beta[2]
Sa7 <- vcov(modela7) ["DHD_TOTAL_SD", "DHD_TOTAL_SD"]

a8 <- modela8@beta[2]
Sa8 <- vcov(modela8) ["DHD_TOTAL_SD", "DHD_TOTAL_SD"]

a9 <- modela9@beta[2]
Sa9 <- vcov(modela9) ["DHD_TOTAL_SD", "DHD_TOTAL_SD"]


### Estimate outcome model and save coefficients ###
# Outcome model
modelb <- (lmer(PF2 ~ DHD_TOTAL_SD + Trp + Kyn + HK + KA + XA + AA + HAA + Pic + QA +
ENROLL_AGE + SEX + Creat + COM_NUMBER_CAT + P_CLIN_CHEM +
WEEKS_SINCE_END_TREAT + STOMA + W0EDU_LEVEL + BMI + MVPA + SMOKER_CURR2 +
Energie_kcal_day + mox.mdursedge30min_h + (1 |PATIENTN), data=Dataset_Sumsc))
summary(modelb)
confint(modelb)

# Save c' coefficient and variance
cp <- modelb@beta[2]
Scp <- vcov(modelb) ["DHD_TOTAL_SD", "DHD_TOTAL_SD"]
```

```
# Save b coefficient and variance
b1 <- modelb@beta[3]
Sb1 <- vcov(modelb) ["Trp", "Trp"]
Covsb1b2 <- vcov(modelb) ["Trp", "Kyn"]
Covsb1b3 <- vcov(modelb) ["Trp", "HK"]
Covsb1b4 <- vcov(modelb) ["Trp", "KA"]
Covsb1b5 <- vcov(modelb) ["Trp", "XA"]
Covsb1b6 <- vcov(modelb) ["Trp", "AA"]
Covsb1b7 <- vcov(modelb) ["Trp", "HAA"]
Covsb1b8 <- vcov(modelb) ["Trp", "Pic"]
Covsb1b9 <- vcov(modelb) ["Trp", "QA"]
Covsb1cp <- vcov(modelb) ["Trp", "DHD_TOTAL_SD"]

b2 <- modelb@beta[4]
Sb2 <- vcov(modelb) ["Kyn", "Kyn"]
Covsb2b3 <- vcov(modelb) ["Kyn", "HK"]
Covsb2b4 <- vcov(modelb) ["Kyn", "KA"]
Covsb2b5 <- vcov(modelb) ["Kyn", "XA"]
Covsb2b6 <- vcov(modelb) ["Kyn", "AA"]
Covsb2b7 <- vcov(modelb) ["Kyn", "HAA"]
Covsb2b8 <- vcov(modelb) ["Kyn", "Pic"]
Covsb2b9 <- vcov(modelb) ["Kyn", "QA"]
Covsb2cp <- vcov(modelb) ["Kyn", "DHD_TOTAL_SD"]

b3 <- modelb@beta[5]
Sb3 <- vcov(modelb) ["HK", "HK"]
Covsb3b4 <- vcov(modelb) ["HK", "KA"]
Covsb3b5 <- vcov(modelb) ["HK", "XA"]
Covsb3b6 <- vcov(modelb) ["HK", "AA"]
Covsb3b7 <- vcov(modelb) ["HK", "HAA"]
Covsb3b8 <- vcov(modelb) ["HK", "Pic"]
Covsb3b9 <- vcov(modelb) ["HK", "QA"]
Covsb3cp <- vcov(modelb) ["HK", "DHD_TOTAL_SD"]

b4 <- modelb@beta[6]
Sb4 <- vcov(modelb) ["KA", "KA"]
Covsb4b5 <- vcov(modelb) ["KA", "XA"]
Covsb4b6 <- vcov(modelb) ["KA", "AA"]
Covsb4b7 <- vcov(modelb) ["KA", "HAA"]
Covsb4b8 <- vcov(modelb) ["KA", "Pic"]
Covsb4b9 <- vcov(modelb) ["KA", "QA"]
Covsb4cp <- vcov(modelb) ["KA", "DHD_TOTAL_SD"]

b5 <- modelb@beta[7]
Sb5 <- vcov(modelb) ["XA", "XA"]
Covsb5b6 <- vcov(modelb) ["XA", "AA"]
Covsb5b7 <- vcov(modelb) ["XA", "HAA"]
Covsb5b8 <- vcov(modelb) ["XA", "Pic"]
Covsb5b9 <- vcov(modelb) ["XA", "QA"]
Covsb5cp <- vcov(modelb) ["XA", "DHD_TOTAL_SD"]

b6 <- modelb@beta[8]
Sb6 <- vcov(modelb) ["AA", "AA"]
Covsb6b7 <- vcov(modelb) ["AA", "HAA"]
Covsb6b8 <- vcov(modelb) ["AA", "Pic"]
Covsb6b9 <- vcov(modelb) ["AA", "QA"]
```

```
Covsb6cp <- vcov(modelb) ["AA", "DHD_TOTAL_SD"]

b7 <- modelb@beta[9]
Sb7 <- vcov(modelb) ["HAA", "HAA"]
Covsb7b8 <- vcov(modelb) ["HAA", "Pic"]
Covsb7b9 <- vcov(modelb) ["HAA", "QA"]
Covsb7cp <- vcov(modelb) ["HAA", "DHD_TOTAL_SD"]

b8 <- modelb@beta[10]
Sb8 <- vcov(modelb) ["Pic", "Pic"]
Covsb8b9 <- vcov(modelb) ["Pic", "QA"]
Covsb8cp <- vcov(modelb) ["Pic", "DHD_TOTAL_SD"]

b9 <- modelb@beta[11]
Sb9 <- vcov(modelb) ["QA", "QA"]
Covsb9cp <- vcov(modelb) ["QA", "DHD_TOTAL_SD"]

### Compute mediation effects with confidence intervals ###
# Indirect effect with Monte Carlo confidence interval
ab1 <- a1*b1
ab1
set.seed(1234)
ci(mu=c(b1=a1, b2=a2, b3=a3, b4=a4, b5=a5, b6=a6, b7=a7, b8=a8, b9=a9, b10=b1, b11=b2,
b12=b3, b13=b4, b14=b5, b15=b6, b16=b7, b17=b8, b18=b9, b19=cp),
Sigma=c(Sa1,0,0,0,0,0,0,0,0,0,0,0,0,0,0,0,0,0,0,Sa2,0,0,0,0,0,0,0,0,0,0,0,0,0,0,0,0,0,0,Sa3,0,0,0,0,0,0,0
,0,0,0,0,0,0,0,0,0,Sa4,0,0,0,0,0,0,0,0,0,0,0,0,0,0,0,0,Sa5,
0,0,0,0,0,0,0,0,0,0,0,0,0,0,Sa6,0,0,0,0,0,0,0,0,0,0,0,0,0,Sa7,0,0,0,0,0,0,0,0,0,0,0,0,Sa8,0,0,0,0,0,0,0,0
,0,0,0,Sa9,0,0,0,0,0,0,0,0,0,0,Sb1,Covsb1b2,Covsb1b3,Covsb1b4,Covsb1b5,Covsb1b6,Covsb1b7,Co
vsb1b8,Covsb1b9,Covsb1cp,Sb2,Covsb2b3,Covsb2b4,Covsb2b5,Covsb2b6,Covsb2b7,Covsb2b8,Co
vsb2b9,Covsb2cp,Sb3,Covsb3b4,Covsb3b5,Covsb3b6,Covsb3b7,Covsb3b8,Covsb3b9,Covsb3cp,Sb
4,Covsb4b5,Covsb4b6,Covsb4b7,Covsb4b8,Covsb4b9,Covsb4cp,Sb5,Covsb5b6,Covsb5b7,Covsb5b
8,Covsb5b9,Covsb5cp,Sb6,Covsb6b7,Covsb6b8,Covsb6b9,Covsb6cp,Sb7,Covsb7b8,Covsb7b9,Cov
sb7cp,Sb8,Covsb8b9,Covsb8cp,Sb9,Covsb9cp,Scp),
  quant=~b1*b10, alpha=0.05, type="MC", n.mc=20000)

ab2 <- a2*b2
ab2
set.seed(1234)
ci(mu=c(b1=a1, b2=a2, b3=a3, b4=a4, b5=a5, b6=a6, b7=a7, b8=a8, b9=a9, b10=b1, b11=b2,
b12=b3, b13=b4, b14=b5, b15=b6, b16=b7, b17=b8, b18=b9, b19=cp),
Sigma=c(Sa1,0,0,0,0,0,0,0,0,0,0,0,0,0,0,0,0,0,0,Sa2,0,0,0,0,0,0,0,0,0,0,0,0,0,0,0,0,0,0,Sa3,0,0,0,0,0,0,0
,0,0,0,0,0,0,0,0,0,Sa4,0,0,0,0,0,0,0,0,0,0,0,0,0,0,0,0,Sa5,
0,0,0,0,0,0,0,0,0,0,0,0,0,0,Sa6,0,0,0,0,0,0,0,0,0,0,0,0,0,Sa7,0,0,0,0,0,0,0,0,0,0,0,0,Sa8,0,0,0,0,0,0,0,0
,0,0,0,Sa9,0,0,0,0,0,0,0,0,0,0,Sb1,Covsb1b2,Covsb1b3,Covsb1b4,Covsb1b5,Covsb1b6,Covsb1b7,Co
vsb1b8,Covsb1b9,Covsb1cp,Sb2,Covsb2b3,Covsb2b4,Covsb2b5,Covsb2b6,Covsb2b7,Covsb2b8,Co
vsb2b9,Covsb2cp,Sb3,Covsb3b4,Covsb3b5,Covsb3b6,Covsb3b7,Covsb3b8,Covsb3b9,Covsb3cp,Sb
4,Covsb4b5,Covsb4b6,Covsb4b7,Covsb4b8,Covsb4b9,Covsb4cp,Sb5,Covsb5b6,Covsb5b7,Covsb5b
8,Covsb5b9,Covsb5cp,Sb6,Covsb6b7,Covsb6b8,Covsb6b9,Covsb6cp,Sb7,Covsb7b8,Covsb7b9,Cov
sb7cp,Sb8,Covsb8b9,Covsb8cp,Sb9,Covsb9cp,Scp),
  quant=~b2*b11, alpha=0.05, type="MC", n.mc=20000)

ab3 <- a3*b3
ab3
set.seed(1234)
ci(mu=c(b1=a1, b2=a2, b3=a3, b4=a4, b5=a5, b6=a6, b7=a7, b8=a8, b9=a9, b10=b1, b11=b2,
b12=b3, b13=b4, b14=b5, b15=b6, b16=b7, b17=b8, b18=b9, b19=cp),
```

```
Sigma=c(Sa1,0,0,0,0,0,0,0,0,0,0,0,0,0,0,0,0,0,0,Sa2,0,0,0,0,0,0,0,0,0,0,0,0,0,0,0,0,0,Sa3,0,0,0,0,0,0,0
,0,0,0,0,0,0,0,0,0,Sa4,0,0,0,0,0,0,0,0,0,0,0,0,0,0,0,Sa5,
0,0,0,0,0,0,0,0,0,0,0,0,0,0,Sa6,0,0,0,0,0,0,0,0,0,0,0,0,0,Sa7,0,0,0,0,0,0,0,0,0,0,0,0,Sa8,0,0,0,0,0,0,0,0
,0,0,0,Sa9,0,0,0,0,0,0,0,0,0,0,Sb1,Covsb1b2,Covsb1b3,Covsb1b4,Covsb1b5,Covsb1b6,Covsb1b7,Co
vsb1b8,Covsb1b9,Covsb1cp,Sb2,Covsb2b3,Covsb2b4,Covsb2b5,Covsb2b6,Covsb2b7,Covsb2b8,Co
vsb2b9,Covsb2cp,Sb3,Covsb3b4,Covsb3b5,Covsb3b6,Covsb3b7,Covsb3b8,Covsb3b9,Covsb3cp,Sb
4,Covsb4b5,Covsb4b6,Covsb4b7,Covsb4b8,Covsb4b9,Covsb4cp,Sb5,Covsb5b6,Covsb5b7,Covsb5b
8,Covsb5b9,Covsb5cp,Sb6,Covsb6b7,Covsb6b8,Covsb6b9,Covsb6cp,Sb7,Covsb7b8,Covsb7b9,Cov
sb7cp,Sb8,Covsb8b9,Covsb8cp,Sb9,Covsb9cp,Scp),
  quant=~b3*b12, alpha=0.05, type="MC", n.mc=20000)

ab4 <- a4*b4
ab4
set.seed(1234)
ci(mu=c(b1=a1, b2=a2, b3=a3, b4=a4, b5=a5, b6=a6, b7=a7, b8=a8, b9=a9, b10=b1, b11=b2,
b12=b3, b13=b4, b14=b5, b15=b6, b16=b7, b17=b8, b18=b9, b19=cp),
Sigma=c(Sa1,0,0,0,0,0,0,0,0,0,0,0,0,0,0,0,0,0,0,Sa2,0,0,0,0,0,0,0,0,0,0,0,0,0,0,0,0,0,Sa3,0,0,0,0,0,0,0
,0,0,0,0,0,0,0,0,0,Sa4,0,0,0,0,0,0,0,0,0,0,0,0,0,0,0,Sa5,
0,0,0,0,0,0,0,0,0,0,0,0,0,0,Sa6,0,0,0,0,0,0,0,0,0,0,0,0,0,Sa7,0,0,0,0,0,0,0,0,0,0,0,0,Sa8,0,0,0,0,0,0,0,0
,0,0,0,Sa9,0,0,0,0,0,0,0,0,0,0,Sb1,Covsb1b2,Covsb1b3,Covsb1b4,Covsb1b5,Covsb1b6,Covsb1b7,Co
vsb1b8,Covsb1b9,Covsb1cp,Sb2,Covsb2b3,Covsb2b4,Covsb2b5,Covsb2b6,Covsb2b7,Covsb2b8,Co
vsb2b9,Covsb2cp,Sb3,Covsb3b4,Covsb3b5,Covsb3b6,Covsb3b7,Covsb3b8,Covsb3b9,Covsb3cp,Sb
4,Covsb4b5,Covsb4b6,Covsb4b7,Covsb4b8,Covsb4b9,Covsb4cp,Sb5,Covsb5b6,Covsb5b7,Covsb5b
8,Covsb5b9,Covsb5cp,Sb6,Covsb6b7,Covsb6b8,Covsb6b9,Covsb6cp,Sb7,Covsb7b8,Covsb7b9,Cov
sb7cp,Sb8,Covsb8b9,Covsb8cp,Sb9,Covsb9cp,Scp),
  quant=~b4*b13, alpha=0.05, type="MC", n.mc=20000)

ab5 <- a5*b5
ab5
set.seed(1234)
ci(mu=c(b1=a1, b2=a2, b3=a3, b4=a4, b5=a5, b6=a6, b7=a7, b8=a8, b9=a9, b10=b1, b11=b2,
b12=b3, b13=b4, b14=b5, b15=b6, b16=b7, b17=b8, b18=b9, b19=cp),
Sigma=c(Sa1,0,0,0,0,0,0,0,0,0,0,0,0,0,0,0,0,0,0,Sa2,0,0,0,0,0,0,0,0,0,0,0,0,0,0,0,0,0,Sa3,0,0,0,0,0,0,0
,0,0,0,0,0,0,0,0,0,Sa4,0,0,0,0,0,0,0,0,0,0,0,0,0,0,0,Sa5,
0,0,0,0,0,0,0,0,0,0,0,0,0,0,Sa6,0,0,0,0,0,0,0,0,0,0,0,0,0,Sa7,0,0,0,0,0,0,0,0,0,0,0,0,Sa8,0,0,0,0,0,0,0,0
,0,0,0,Sa9,0,0,0,0,0,0,0,0,0,0,Sb1,Covsb1b2,Covsb1b3,Covsb1b4,Covsb1b5,Covsb1b6,Covsb1b7,Co
vsb1b8,Covsb1b9,Covsb1cp,Sb2,Covsb2b3,Covsb2b4,Covsb2b5,Covsb2b6,Covsb2b7,Covsb2b8,Co
vsb2b9,Covsb2cp,Sb3,Covsb3b4,Covsb3b5,Covsb3b6,Covsb3b7,Covsb3b8,Covsb3b9,Covsb3cp,Sb
4,Covsb4b5,Covsb4b6,Covsb4b7,Covsb4b8,Covsb4b9,Covsb4cp,Sb5,Covsb5b6,Covsb5b7,Covsb5b
8,Covsb5b9,Covsb5cp,Sb6,Covsb6b7,Covsb6b8,Covsb6b9,Covsb6cp,Sb7,Covsb7b8,Covsb7b9,Cov
sb7cp,Sb8,Covsb8b9,Covsb8cp,Sb9,Covsb9cp,Scp),
  quant=~b5*b14, alpha=0.05, type="MC", n.mc=20000)

ab6 <- a6*b6
ab6
set.seed(1234)
ci(mu=c(b1=a1, b2=a2, b3=a3, b4=a4, b5=a5, b6=a6, b7=a7, b8=a8, b9=a9, b10=b1, b11=b2,
b12=b3, b13=b4, b14=b5, b15=b6, b16=b7, b17=b8, b18=b9, b19=cp),
Sigma=c(Sa1,0,0,0,0,0,0,0,0,0,0,0,0,0,0,0,0,0,0,Sa2,0,0,0,0,0,0,0,0,0,0,0,0,0,0,0,0,0,Sa3,0,0,0,0,0,0,0
,0,0,0,0,0,0,0,0,0,Sa4,0,0,0,0,0,0,0,0,0,0,0,0,0,0,0,Sa5,
0,0,0,0,0,0,0,0,0,0,0,0,0,0,Sa6,0,0,0,0,0,0,0,0,0,0,0,0,0,Sa7,0,0,0,0,0,0,0,0,0,0,0,0,Sa8,0,0,0,0,0,0,0,0
,0,0,0,Sa9,0,0,0,0,0,0,0,0,0,0,Sb1,Covsb1b2,Covsb1b3,Covsb1b4,Covsb1b5,Covsb1b6,Covsb1b7,Co
vsb1b8,Covsb1b9,Covsb1cp,Sb2,Covsb2b3,Covsb2b4,Covsb2b5,Covsb2b6,Covsb2b7,Covsb2b8,Co
vsb2b9,Covsb2cp,Sb3,Covsb3b4,Covsb3b5,Covsb3b6,Covsb3b7,Covsb3b8,Covsb3b9,Covsb3cp,Sb
4,Covsb4b5,Covsb4b6,Covsb4b7,Covsb4b8,Covsb4b9,Covsb4cp,Sb5,Covsb5b6,Covsb5b7,Covsb5b
```

```
8,Covsb5b9,Covsb5cp,Sb6,Covsb6b7,Covsb6b8,Covsb6b9,Covsb6cp,Sb7,Covsb7b8,Covsb7b9,Cov
sb7cp,Sb8,Covsb8b9,Covsb8cp,Sb9,Covsb9cp,Scp),
  quant=~b6*b15, alpha=0.05, type="MC", n.mc=20000)

ab7 <- a7*b7
ab7
set.seed(1234)
ci(mu=c(b1=a1, b2=a2, b3=a3, b4=a4, b5=a5, b6=a6, b7=a7, b8=a8, b9=a9, b10=b1, b11=b2,
b12=b3, b13=b4, b14=b5, b15=b6, b16=b7, b17=b8, b18=b9, b19=cp),
Sigma=c(Sa1,0,0,0,0,0,0,0,0,0,0,0,0,0,0,0,0,0,0,Sa2,0,0,0,0,0,0,0,0,0,0,0,0,0,0,0,0,0,Sa3,0,0,0,0,0,0,0
,0,0,0,0,0,0,0,0,0,Sa4,0,0,0,0,0,0,0,0,0,0,0,0,0,0,0,Sa5,
0,0,0,0,0,0,0,0,0,0,0,0,0,0,Sa6,0,0,0,0,0,0,0,0,0,0,0,0,0,Sa7,0,0,0,0,0,0,0,0,0,0,0,0,Sa8,0,0,0,0,0,0,0,0
,0,0,0,Sa9,0,0,0,0,0,0,0,0,0,0,Sb1,Covsb1b2,Covsb1b3,Covsb1b4,Covsb1b5,Covsb1b6,Covsb1b7,Co
vsb1b8,Covsb1b9,Covsb1cp,Sb2,Covsb2b3,Covsb2b4,Covsb2b5,Covsb2b6,Covsb2b7,Covsb2b8,Co
vsb2b9,Covsb2cp,Sb3,Covsb3b4,Covsb3b5,Covsb3b6,Covsb3b7,Covsb3b8,Covsb3b9,Covsb3cp,Sb
4,Covsb4b5,Covsb4b6,Covsb4b7,Covsb4b8,Covsb4b9,Covsb4cp,Sb5,Covsb5b6,Covsb5b7,Covsb5b
8,Covsb5b9,Covsb5cp,Sb6,Covsb6b7,Covsb6b8,Covsb6b9,Covsb6cp,Sb7,Covsb7b8,Covsb7b9,Cov
sb7cp,Sb8,Covsb8b9,Covsb8cp,Sb9,Covsb9cp,Scp),
  quant=~b7*b16, alpha=0.05, type="MC", n.mc=20000)

ab8 <- a8*b8
ab8
set.seed(1234)
ci(mu=c(b1=a1, b2=a2, b3=a3, b4=a4, b5=a5, b6=a6, b7=a7, b8=a8, b9=a9, b10=b1, b11=b2,
b12=b3, b13=b4, b14=b5, b15=b6, b16=b7, b17=b8, b18=b9, b19=cp),
Sigma=c(Sa1,0,0,0,0,0,0,0,0,0,0,0,0,0,0,0,0,0,0,Sa2,0,0,0,0,0,0,0,0,0,0,0,0,0,0,0,0,0,Sa3,0,0,0,0,0,0,0
,0,0,0,0,0,0,0,0,0,Sa4,0,0,0,0,0,0,0,0,0,0,0,0,0,0,0,Sa5,
0,0,0,0,0,0,0,0,0,0,0,0,0,0,Sa6,0,0,0,0,0,0,0,0,0,0,0,0,0,Sa7,0,0,0,0,0,0,0,0,0,0,0,0,Sa8,0,0,0,0,0,0,0,0
,0,0,0,Sa9,0,0,0,0,0,0,0,0,0,0,Sb1,Covsb1b2,Covsb1b3,Covsb1b4,Covsb1b5,Covsb1b6,Covsb1b7,Co
vsb1b8,Covsb1b9,Covsb1cp,Sb2,Covsb2b3,Covsb2b4,Covsb2b5,Covsb2b6,Covsb2b7,Covsb2b8,Co
vsb2b9,Covsb2cp,Sb3,Covsb3b4,Covsb3b5,Covsb3b6,Covsb3b7,Covsb3b8,Covsb3b9,Covsb3cp,Sb
4,Covsb4b5,Covsb4b6,Covsb4b7,Covsb4b8,Covsb4b9,Covsb4cp,Sb5,Covsb5b6,Covsb5b7,Covsb5b
8,Covsb5b9,Covsb5cp,Sb6,Covsb6b7,Covsb6b8,Covsb6b9,Covsb6cp,Sb7,Covsb7b8,Covsb7b9,Cov
sb7cp,Sb8,Covsb8b9,Covsb8cp,Sb9,Covsb9cp,Scp),
  quant=~b8*b17, alpha=0.05, type="MC", n.mc=20000)

ab9 <- a9*b9
ab9
set.seed(1234)
ci(mu=c(b1=a1, b2=a2, b3=a3, b4=a4, b5=a5, b6=a6, b7=a7, b8=a8, b9=a9, b10=b1, b11=b2,
b12=b3, b13=b4, b14=b5, b15=b6, b16=b7, b17=b8, b18=b9, b19=cp),
Sigma=c(Sa1,0,0,0,0,0,0,0,0,0,0,0,0,0,0,0,0,0,0,Sa2,0,0,0,0,0,0,0,0,0,0,0,0,0,0,0,0,0,Sa3,0,0,0,0,0,0,0
,0,0,0,0,0,0,0,0,0,Sa4,0,0,0,0,0,0,0,0,0,0,0,0,0,0,0,Sa5,
0,0,0,0,0,0,0,0,0,0,0,0,0,0,Sa6,0,0,0,0,0,0,0,0,0,0,0,0,0,Sa7,0,0,0,0,0,0,0,0,0,0,0,0,Sa8,0,0,0,0,0,0,0,0
,0,0,0,Sa9,0,0,0,0,0,0,0,0,0,0,Sb1,Covsb1b2,Covsb1b3,Covsb1b4,Covsb1b5,Covsb1b6,Covsb1b7,Co
vsb1b8,Covsb1b9,Covsb1cp,Sb2,Covsb2b3,Covsb2b4,Covsb2b5,Covsb2b6,Covsb2b7,Covsb2b8,Co
vsb2b9,Covsb2cp,Sb3,Covsb3b4,Covsb3b5,Covsb3b6,Covsb3b7,Covsb3b8,Covsb3b9,Covsb3cp,Sb
4,Covsb4b5,Covsb4b6,Covsb4b7,Covsb4b8,Covsb4b9,Covsb4cp,Sb5,Covsb5b6,Covsb5b7,Covsb5b
8,Covsb5b9,Covsb5cp,Sb6,Covsb6b7,Covsb6b8,Covsb6b9,Covsb6cp,Sb7,Covsb7b8,Covsb7b9,Cov
sb7cp,Sb8,Covsb8b9,Covsb8cp,Sb9,Covsb9cp,Scp),
  quant=~b9*b18, alpha=0.05, type="MC", n.mc=20000)

abtot <- ab1 + ab2 + ab3 + ab4 + ab5 + ab6 + ab7 + ab8 + ab9
abtot
set.seed(1234)
```

```
ci(mu=c(b1=a1, b2=a2, b3=a3, b4=a4, b5=a5, b6=a6, b7=a7, b8=a8, b9=a9, b10=b1, b11=b2,
b12=b3, b13=b4, b14=b5, b15=b6, b16=b7, b17=b8, b18=b9, b19=cp),
Sigma=c(Sa1,0,0,0,0,0,0,0,0,0,0,0,0,0,0,0,0,0,0,Sa2,0,0,0,0,0,0,0,0,0,0,0,0,0,0,0,0,0,Sa3,0,0,0,0,0,0,0
,0,0,0,0,0,0,0,0,0,Sa4,0,0,0,0,0,0,0,0,0,0,0,0,0,0,0,Sa5,
0,0,0,0,0,0,0,0,0,0,0,0,0,0,Sa6,0,0,0,0,0,0,0,0,0,0,0,0,0,Sa7,0,0,0,0,0,0,0,0,0,0,0,0,Sa8,0,0,0,0,0,0,0,0
,0,0,0,Sa9,0,0,0,0,0,0,0,0,0,0,Sb1,Covsb1b2,Covsb1b3,Covsb1b4,Covsb1b5,Covsb1b6,Covsb1b7,Co
vsb1b8,Covsb1b9,Covsb1cp,Sb2,Covsb2b3,Covsb2b4,Covsb2b5,Covsb2b6,Covsb2b7,Covsb2b8,Co
vsb2b9,Covsb2cp,Sb3,Covsb3b4,Covsb3b5,Covsb3b6,Covsb3b7,Covsb3b8,Covsb3b9,Covsb3cp,Sb
4,Covsb4b5,Covsb4b6,Covsb4b7,Covsb4b8,Covsb4b9,Covsb4cp,Sb5,Covsb5b6,Covsb5b7,Covsb5b
8,Covsb5b9,Covsb5cp,Sb6,Covsb6b7,Covsb6b8,Covsb6b9,Covsb6cp,Sb7,Covsb7b8,Covsb7b9,Cov
sb7cp,Sb8,Covsb8b9,Covsb8cp,Sb9,Covsb9cp,Scp),
  quant=~b1*b10+b2*b11+b3*b12+b4*b13+b5*b14+ b6*b15+b7*b16+b8*b17+b9*b18, alpha=0.05,
type="MC", n.mc=20000)

# Direct effect with confidence interval
direct <- cp
direct
set.seed(1234)
ci(mu=c(b1=a1, b2=a2, b3=a3, b4=a4, b5=a5, b6=a6, b7=a7, b8=a8, b9=a9, b10=b1, b11=b2,
b12=b3, b13=b4, b14=b5, b15=b6, b16=b7, b17=b8, b18=b9, b19=cp),
Sigma=c(Sa1,0,0,0,0,0,0,0,0,0,0,0,0,0,0,0,0,0,0,Sa2,0,0,0,0,0,0,0,0,0,0,0,0,0,0,0,0,0,Sa3,0,0,0,0,0,0,0
,0,0,0,0,0,0,0,0,0,Sa4,0,0,0,0,0,0,0,0,0,0,0,0,0,0,0,Sa5,
0,0,0,0,0,0,0,0,0,0,0,0,0,0,Sa6,0,0,0,0,0,0,0,0,0,0,0,0,0,Sa7,0,0,0,0,0,0,0,0,0,0,0,0,Sa8,0,0,0,0,0,0,0,0
,0,0,0,Sa9,0,0,0,0,0,0,0,0,0,0,Sb1,Covsb1b2,Covsb1b3,Covsb1b4,Covsb1b5,Covsb1b6,Covsb1b7,Co
vsb1b8,Covsb1b9,Covsb1cp,Sb2,Covsb2b3,Covsb2b4,Covsb2b5,Covsb2b6,Covsb2b7,Covsb2b8,Co
vsb2b9,Covsb2cp,Sb3,Covsb3b4,Covsb3b5,Covsb3b6,Covsb3b7,Covsb3b8,Covsb3b9,Covsb3cp,Sb
4,Covsb4b5,Covsb4b6,Covsb4b7,Covsb4b8,Covsb4b9,Covsb4cp,Sb5,Covsb5b6,Covsb5b7,Covsb5b
8,Covsb5b9,Covsb5cp,Sb6,Covsb6b7,Covsb6b8,Covsb6b9,Covsb6cp,Sb7,Covsb7b8,Covsb7b9,Cov
sb7cp,Sb8,Covsb8b9,Covsb8cp,Sb9,Covsb9cp,Scp),
  quant=~b19, alpha=0.05, type="asymp", n.asymp=20000)

# Total effect with confidence interval
total <- abtot+cp
total
set.seed(1234)
ci(mu=c(b1=a1, b2=a2, b3=a3, b4=a4, b5=a5, b6=a6, b7=a7, b8=a8, b9=a9, b10=b1, b11=b2,
b12=b3, b13=b4, b14=b5, b15=b6, b16=b7, b17=b8, b18=b9, b19=cp),
Sigma=c(Sa1,0,0,0,0,0,0,0,0,0,0,0,0,0,0,0,0,0,0,Sa2,0,0,0,0,0,0,0,0,0,0,0,0,0,0,0,0,0,Sa3,0,0,0,0,0,0,0
,0,0,0,0,0,0,0,0,0,Sa4,0,0,0,0,0,0,0,0,0,0,0,0,0,0,0,Sa5,
0,0,0,0,0,0,0,0,0,0,0,0,0,0,Sa6,0,0,0,0,0,0,0,0,0,0,0,0,0,Sa7,0,0,0,0,0,0,0,0,0,0,0,0,Sa8,0,0,0,0,0,0,0,0
,0,0,0,Sa9,0,0,0,0,0,0,0,0,0,0,Sb1,Covsb1b2,Covsb1b3,Covsb1b4,Covsb1b5,Covsb1b6,Covsb1b7,Co
vsb1b8,Covsb1b9,Covsb1cp,Sb2,Covsb2b3,Covsb2b4,Covsb2b5,Covsb2b6,Covsb2b7,Covsb2b8,Co
vsb2b9,Covsb2cp,Sb3,Covsb3b4,Covsb3b5,Covsb3b6,Covsb3b7,Covsb3b8,Covsb3b9,Covsb3cp,Sb
4,Covsb4b5,Covsb4b6,Covsb4b7,Covsb4b8,Covsb4b9,Covsb4cp,Sb5,Covsb5b6,Covsb5b7,Covsb5b
8,Covsb5b9,Covsb5cp,Sb6,Covsb6b7,Covsb6b8,Covsb6b9,Covsb6cp,Sb7,Covsb7b8,Covsb7b9,Cov
sb7cp,Sb8,Covsb8b9,Covsb8cp,Sb9,Covsb9cp,Scp),
  quant=~b1*b10+b2*b11+b3*b12+b4*b13+b5*b14+ b6*b15+b7*b16+b8*b17+b9*b18+b19,
alpha=0.05, type="asymp", n.asymp=20000)
```

1.4 Single mediator model: Macronutrient intake – Physical functioning

```
###KH-KTR-PHYSICAL FUNCTIONING

### Estimate mediator model and save coefficients ###
# Mediator models
modela1 <- (lmer(KTR ~ Koolhydraten_kcal_day_SD + Vet_kcal_day_SD + Eiwit_kcal_day_SD +
Alcohol_kcal_day_SD + Voedingsvezel_kcal_day_SD + ENROLL_AGE + SEX + Creat +
COM_NUMBER_CAT + P_CLIN_CHEM + WEEKS_SINCE_END_TREAT + STOMA +
W0EDU_LEVEL + BMI + MVPA + SMOKER_CURR2 + mox.mdursedge30min_h + (1 |PATIENTN),
data=Dataset_Sumsc))
summary(modela1)
confint(modela1)

# Save a coefficients and variance
a1 <- modela1@beta[2]
Sa1 <- vcov(modela1) ["Koolhydraten_kcal_day_SD", "Koolhydraten_kcal_day_SD"]


### Estimate outcome model and save coefficients ###
# Outcome model
modelb <- (lmer(PF2 ~ Koolhydraten_kcal_day_SD + Vet_kcal_day_SD + Eiwit_kcal_day_SD +
Alcohol_kcal_day_SD + Voedingsvezel_kcal_day_SD + KTR + ENROLL_AGE + SEX + Creat +
COM_NUMBER_CAT + P_CLIN_CHEM + WEEKS_SINCE_END_TREAT + STOMA +
W0EDU_LEVEL + BMI + MVPA + SMOKER_CURR2 + mox.mdursedge30min_h + (1 |PATIENTN),
data=Dataset_Sumsc))
summary(modelb)
confint(modelb)

# Save c' coefficient and variance
cp <- modelb@beta[2]
Scp <- vcov(modelb) ["Koolhydraten_kcal_day_SD", "Koolhydraten_kcal_day_SD"]

# Save b coefficient and variance
b1 <- modelb@beta[7]
Sb1 <- vcov(modelb) ["KTR", "KTR"]
Covsb1cp <- vcov(modelb) ["KTR", "Koolhydraten_kcal_day_SD"]

### Compute mediation effects with confidence intervals ###
# Indirect effect with Monte Carlo confidence interval
ab1 <- a1*b1
ab1
set.seed(1234)
ci(mu=c(b1=a1, b2=b1, b3=cp), Sigma=c(Sa1,0,0,Sb1, Covsb1cp,Scp),
  quant=~b1*b2, alpha=0.05, type="MC", n.mc=20000)

# Direct effect with confidence interval
direct <- cp
direct
set.seed(1234)
ci(mu=c(b1=a1, b2=b1, b3=cp), Sigma=c(Sa1,0,0,Sb1, Covsb1cp,Scp),
  quant=~b3, alpha=0.05, type="asymp", n.asymp=20000)

# Total effect with confidence interval
total <- ab1+cp
```

```
total
set.seed(1234)
ci(mu=c(b1=a1, b2=b1, b3=cp), Sigma=c(Sa1,0,0,Sb1, Covsb1cp,Scp),
   quant=~b1*b2+b3, alpha=0.05, type="asymp", n.asymp=20000)
```

1.5 Single mediator model: Micronutrient intake – Physical functioning

```
###VITAMINE B2-KTR-PHYSICAL FUNCTIONING

### Estimate mediator model and save coefficients ###
# Mediator models
modela1 <- (lmer(KTR ~ VITB2_mg_day_SD + ENROLL_AGE + SEX + Creat + COM_NUMBER_CAT
+ P_CLIN_CHEM + WEEKS_SINCE_END_TREAT + STOMA + W0EDU_LEVEL + BMI + MVPA +
SMOKER_CURR2 + Energie_kcal_day + Alcohol_totaal_g_day + mox.mdursedge30min_h + (1
|PATIENTN), data=Dataset_Sumsc))
summary(modela1)
confint(modela1)

# Save a coefficients and variance
a1 <- modela1@beta[2]
Sa1 <- vcov(modela1) ["VITB2_mg_day_SD", "VITB2_mg_day_SD"]


### Estimate outcome model and save coefficients ###
# Outcome model
# Outcome model
modelb <- (lmer(PF2 ~ VITB2_mg_day_SD + KTR + ENROLL_AGE + SEX + Creat +
COM_NUMBER_CAT + P_CLIN_CHEM + WEEKS_SINCE_END_TREAT + STOMA +
W0EDU_LEVEL + BMI + MVPA + SMOKER_CURR2 + Energie_kcal_day + Alcohol_totaal_g_day +
mox.mdursedge30min_h + (1 |PATIENTN), data=Dataset_Sumsc))
summary(modelb)
confint(modelb)

# Save c' coefficient and variance
cp <- modelb@beta[2]
Scp <- vcov(modelb) ["VITB2_mg_day_SD", "VITB2_mg_day_SD"]

# Save b coefficient and variance
b1 <- modelb@beta[3]
Sb1 <- vcov(modelb) ["KTR", "KTR"]
Covsb1cp <- vcov(modelb) ["KTR", "VITB2_mg_day_SD"]

### Compute mediation effects with confidence intervals ###
# Indirect effect with Monte Carlo confidence interval
ab1 <- a1*b1
ab1
set.seed(1234)
ci(mu=c(b1=a1, b2=b1, b3=cp), Sigma=c(Sa1,0,0,Sb1, Covsb1cp,Scp),
  quant=~b1*b2, alpha=0.05, type="MC", n.mc=20000)

# Direct effect with confidence interval
direct <- cp
direct
set.seed(1234)
ci(mu=c(b1=a1, b2=b1, b3=cp), Sigma=c(Sa1,0,0,Sb1, Covsb1cp,Scp),
  quant=~b3, alpha=0.05, type="asymp", n.asymp=20000)

# Total effect with confidence interval
total <- ab1+cp
total
```

```
set.seed(1234)
ci(mu=c(b1=a1, b2=b1, b3=cp), Sigma=c(Sa1,0,0,Sb1, Covsb1cp,Scp),
  quant=~b1*b2+b3, alpha=0.05, type="asymp", n.asymp=20000)
```

1.6 Single mediator model: Dietary pattern – Physical functioning

```
###DHD-KTR-PHYSICAL FUNCTIONING

### Estimate mediator model and save coefficients ###
# Mediator models
modela1 <- (lmer(KTR ~ DHD_TOTAL_SD + ENROLL_AGE + SEX + Creat + COM_NUMBER_CAT +
P_CLIN_CHEM + WEEKS_SINCE_END_TREAT + STOMA + W0EDU_LEVEL + BMI + MVPA +
SMOKER_CURR2 + Energie_kcal_day + mox.mdursedge30min_h + (1 |PATIENTN),
data=Dataset_Sumsc))
summary(modela1)
confint(modela1)

# Save a coefficients and variance
a1 <- modela1@beta[2]
Sa1 <- vcov(modela1) ["DHD_TOTAL_SD", "DHD_TOTAL_SD"]


### Estimate outcome model and save coefficients ###
# Outcome model
modelb <- (lmer(PF2 ~ DHD_TOTAL_SD + KTR + ENROLL_AGE + SEX + Creat +
COM_NUMBER_CAT + P_CLIN_CHEM + WEEKS_SINCE_END_TREAT + STOMA +
W0EDU_LEVEL + BMI + MVPA + SMOKER_CURR2 + Energie_kcal_day + mox.mdursedge30min_h
+ (1 |PATIENTN), data=Dataset_Sumsc))
summary(modelb)
confint(modelb)

# Save c' coefficient and variance
cp <- modelb@beta[2]
Scp <- vcov(modelb) ["DHD_TOTAL_SD", "DHD_TOTAL_SD"]

# Save b coefficient and variance
b1 <- modelb@beta[3]
Sb1 <- vcov(modelb) ["KTR", "KTR"]
Covsb1cp <- vcov(modelb) ["KTR", "DHD_TOTAL_SD"]

### Compute mediation effects with confidence intervals ###
# Indirect effect with Monte Carlo confidence interval
ab1 <- a1*b1
ab1
set.seed(1234)
ci(mu=c(b1=a1, b2=b1, b3=cp), Sigma=c(Sa1,0,0,Sb1, Covsb1cp,Scp),
  quant=~b1*b2, alpha=0.05, type="MC", n.mc=20000)

# Direct effect with confidence interval
direct <- cp
direct
set.seed(1234)
ci(mu=c(b1=a1, b2=b1, b3=cp), Sigma=c(Sa1,0,0,Sb1, Covsb1cp,Scp),
  quant=~b3, alpha=0.05, type="asymp", n.asymp=20000)

# Total effect with confidence interval
total <- ab1+cp
total
set.seed(1234)
```

```
ci(mu=c(b1=a1, b2=b1, b3=cp), Sigma=c(Sa1,0,0,Sb1, Covsb1cp,Scp),
   quant=~b1*b2+b3, alpha=0.05, type="asymp", n.asymp=20000)
```

2. Supplementary Tables

Supplementary Table 1. Analysis of all KP metabolites as mediators in the longitudinal association of macronutrient intake with global QoL and the EORTC summary score; parallel-multiple mediator model

			**Global QoL (0 – 100)**					**Summary score (0 – 100)**				
**Exposure**	**Mediator**	**a-path**	**b-path**	**Mediator-specific indirect effect (a*b)**	**Total indirect effect**	**Direct effect**	**Total effect**	**b-path**	**Mediator-specific indirect effect (a*b)**	**Total indirect effect**	**Direct effect**	**Total effect**
**Macronutrient intake**												
Total carbohydrates *(per SD =230 kcal/day)*	Trp	-1.65*	-0.09	0.15 (-0.15, 0.57)	-0.35 (-1.33, 0.63)	**-2.83 (-5.17, -0.49)**	**-3.18 (-5.54, -0.82)**	-0.00	0.00 (-0.20, 0.22)	-0.24 (-0.77, 0.28)	**-2.08 (-3.50, -0.67)**	**-2.32 (-3.72, -0.91)**
	Kyn	0.05	1.07	0.05 (-0.29, 0.44)				0.58	0.03 (-0.18, 0.27)			
	HK	0.79	-0.06	-0.05 (-0.35, 0.19)				-0.05*	-0.04 (-0.26, 0.15)			
	KA	-2.89*	0.04	-0.11 (-0.56, 0.24)				0.05	-0.14 (-0.46, 0.07)			
	XA	-1.47*	0.53*	**-0.77 (-1.65, -0.14)**				0.19	-0.28 (-0.73, 0.05)			
	AA	-0.53	-0.08	0.04 (-0.10, 0.26)				-0.03	0.02 (-0.07, 0.13)			
	HAA	-0.91	-0.11	0.10 (-0.13, 0.45)				-0.03	0.02 (-0.09, 0.18)			
	Pic	-2.45*	-0.04	0.09 (-0.24, 0.48)				-0.05	0.12 (-0.07, 0.37)			
	QA	23.06	0.01	0.15 (-0.10, 0.55)				0.00	0.04 (-0.08, 0.22)			
Mono- and disaccharides *(per SD =137 kcal/day)*	Trp	-0.90	-0.09	0.08 (-0.09, 0.36)	-0.15 (-0.87, 0.57)	**-1.96 (-3.74, -0.18)**	**-2.11 (-3.92, -0.30)**	-0.00	0.00 (-0.12, 0.13)	-0.07 (-0.45, 0.32)	**-1.15 (-2.23, -0.07)**	**-1.22, (-2.30, -0.14)**
	Kyn	0.01	1.01	0.01 (-0.15, 0.22)				0.60	0.01 (-0.09, 0.13)			
	HK	-1.51	-0.06	0.10 (-0.07, 0.37)				-0.05*	0.08 (-0.05, 0.27)			
	KA	-2.19*	0.04	-0.08 (-0.43, 0.18)				0.05	-0.11 (-0.35, 0.05)			
	XA	-1.02*	0.52*	**-0.53 (-1.18, -0.08)**				0.19	-0.19 (-0.52, 0.03)			
	AA	-0.20	-0.08	0.02 (-0.08, 0.15)				-0.03	0.01 (-0.05, 0.08)			
	HAA	-0.23	-0.11	0.02 (-0.17, 0.25)				-0.03	0.01 (-0.08, 0.10)			
	Pic	-1.95*	-0.04	0.08 (-0.19, 0.38)				-0.05	0.09 (-0.06, 0.29)			
	QA	23.11	0.01*	0.16 (-0.04, 0.48)				0.00	0.04 (-0.08, 0.19)			
Polysaccharides *(per SD =140 kcal/day)*	Trp	-1.14	-0.09	0.10 (-0.12, 0.46)	-0.32 (-1.31, 0.62)	-1.27 (-3.61, 1.07)	-1.59 (-3.98, 0.80)	-0.00	0.00 (-0.16, 0.17)	-0.27 (-0.80, 0.23)	-1.41 (-2.83, 0.01)	**-1.67 (-3.10, -0.25)**
	Kyn	0.05	1.01	0.05 (-0.31, 0.46)				0.60	0.03 (-0.19, 0.28)			
	HK	3.79*	-0.06	-0.24 (-0.71, 0.05)				-0.05*	**-0.20 (-0.51, -0.00)**			
	KA	-1.00	0.04	-0.04 (-0.32, 0.17)				0.05	-0.05 (-0.27, 0.11)			
	XA	-0.67	0.52*	-0.35 (-1.06, 0.16)				0.19	-0.13 (-0.45, 0.07)			
	AA	-0.51	-0.08	0.04 (-0.10, 0.25)				-0.03	0.02 (-0.07, 0.13)			
	HAA	-1.10	-0.11	0.12 (-0.12, 0.49)				-0.03	0.03 (-0.10, 0.21)			
	Pic	-0.68	-0.04	0.03 (-0.13, 0.24)				-0.05	0.03 (-0.08, 0.19)			
	QA	-2.31	0.01*	-0.02 (-0.34, 0.29)				0.00	-0.00 (-0.13, 0.11)			
Total protein	Trp	0.43	-0.09	-0.04 (-0.30, 0.14)	0.46	0.64	1.10	-0.00	-0.00 (-0.11, 0.10)	0.14	0.52	0.66
	Kyn	0.02	1.07	0.03 (-0.20, 0.30)				0.58	0.01 (-0.12, 0.18)			

(per SD =69 kcal/day)	HK	3.38*	-0.06	-0.21 (-0.63, 0.06)	(-0.39, 1.31)	(-1.50, 2.78)	(-1.09, 3.30)	-0.05*	-0.18 (-0.46, 0.00)	(-0.32, 0.59)	(-0.74, 1.78)	(-0.62, 1.94)
	KA	3.60*	0.04	0.14 (-0.30, 0.66)				0.05	0.17 (-0.08, 0.52)			
	XA	1.65*	0.53*	**0.87 (0.20, 1.75)**				0.19	0.31 (-0.05, 0.78)			
	AA	0.92*	-0.08	-0.08 (-0.36, 0.13)				-0.03	-0.03 (-0.18, 0.09)			
	HAA	2.47*	-0.11	-0.27 (-0.79, 0.08)				-0.03	-0.06 (-0.34, 0.17)			
	Pic	2.46*	-0.04	-0.09 (-0.48, 0.23)				-0.05	-0.12 (-0.37, 0.07)			
	QA	18.13	0.01	0.12 (-0.11, 0.46)				0.00	0.03 (-0.08, 0.18)			
Animal-based protein *(per SD =55 kcal/day)*	Trp	0.30	-0.10	-0.03 (-0.23, 0.12)	0.40 (-0.29, 1.11)	0.39 (-1.31, 2.09)	0.79 (-0.97, 2.55)	-0.00	-0.00 (-0.08, 0.08)	0.11 (-0.26, 0.48)	0.48 (-0.54, 1.49)	0.58 (-0.44, 1.61)
	Kyn	0.02	1.22	0.02 (-0.15, 0.24)				0.57	0.01 (-0.10, 0.14)			
	HK	2.59*	-0.07	-0.17 (-0.50, 0.04)				-0.05*	-0.14 (-0.36, 0.00)			
	KA	2.70*	0.03	0.07 (-0.27, 0.46)				0.05	0.13 (-0.06, 0.40)			
	XA	1.35*	0.56*	**0.76 (0.20, 1.48)**				0.19	0.25 (-0.05, 0.64)			
	AA	0.75*	-0.09	-0.07 (-0.30, 0.10)				-0.03	-0.02 (-0.15, 0.08)			
	HAA	1.95*	-0.11	-0.21 (-0.62, 0.07)				-0.03	-0.05 (-0.27, 0.13)			
	Pic	2.04*	-0.04	-0.08 (-0.39, 0.19)				-0.05	-0.10 (-0.30, 0.06)			
	QA	15.22	0.01*	0.11 (-0.08, 0.39)				0.00	0.02 (-0.06, 0.15)			
Plant-based protein *(per SD =33 kcal/day)*	Trp	0.25	-0.10	-0.02 (-0.35, 0.24)	-0.05 (-1.18, 0.98)	**3.36 (0.58, 6.15)**	**3.32 (0.42, 6.22)**	-0.00	-0.00 (-0.13, 0.12)	0.05 (-0.52, 0.58)	0.19 (-1.48, 1.85)	0.23 (-1.46, 1.93)
	Kyn	0.01	1.22	0.01 (-0.22, 0.29)				0.57	0.01 (-0.14, 0.17)			
	HK	1.41	-0.07	-0.09 (-0.48, 0.20)				-0.05*	-0.08 (-0.37, 0.15)			
	KA	2.60	0.03	0.07 (-0.30, 0.50)				0.05	0.13 (-0.08, 0.47)			
	XA	0.22	0.56*	0.12 (-0.61, 0.91)				0.19	0.04 (-0.23, 0.36)			
	AA	0.33	-0.09	-0.03 (-0.26, 0.13)				-0.03	-0.01 (-0.13, 0.08)			
	HAA	0.34	-0.11	-0.04 (-0.40, 0.27)				-0.03	-0.01 (-0.16, 0.12)			
	Pic	0.39	-0.04	-0.01 (-0.23, 0.17)				-0.05	-0.02 (-0.20, 0.12)			
	QA	-7.66	0.01*	-0.05 (-0.45, 0.30)				0.00	-0.01 (-0.16, 0.11)			
Total fat *(per SD =207 kcal/day)*	Trp	1.71*	-0.09	-0.15 (-0.57, 0.15)	-0.05 (-0.87, 0.77)	2.11 (-0.04, 4.25)	2.06 (-0.16, 4.28)	-0.00	-0.00 (-0.21, 0.20)	0.07 (-0.35, 0.49)	1.19 (-0.08, 2.46)	1.26 (-0.03, 2.55)
	Kyn	0.02	1.07	0.02 (-0.19, 0.29)				0.58	0.01 (-0.12, 0.18)			
	HK	-1.77	-0.06	0.11 (-0.10, 0.43)				-0.05*	0.09 (-0.07, 0.33)			
	KA	-0.62	0.04	-0.02 (-0.26, 0.16)				0.05	-0.03 (-0.23, 0.12)			
	XA	0.11	0.53*	0.06 (-0.48, 0.61)				0.19	0.02 (-0.19, 0.26)			
	AA	0.33	-0.08	-0.03 (-0.21, 0.10)				-0.03	-0.01 (-0.11, 0.06)			
	HAA	0.30	-0.11	-0.03 (-0.32, 0.20)				-0.03	-0.01 (-0.13, 0.09)			
	Pic	0.19	-0.04	-0.01 (-0.17, 0.13)				-0.05	-0.01 (-0.14, 0.10)			
	QA	-0.24	0.01	-0.00 (-0.28, 0.27)				0.00	-0.00 (-0.10, 0.10)			
Saturated fat *(per SD =90 kcal/day)*	Trp	1.50	-0.09	-0.13 (-0.52, 0.13)	0.02 (-0.80, 0.85)	1.08 (-1.10, 3.25)	1.09 (-1.16, 3.34)	-0.00	-0.01 (-0.20, 0.18)	-0.02 (-0.44, 0.42)	**1.41 (0.11, 2.71)**	**1.39 (0.07, 2.71)**
	Kyn	0.05*	1.06	0.05 (-0.32, 0.47)				0.41	0.02 (-0.21, 0.27)			
	HK	0.77	-0.06	-0.05 (-0.33, 0.18)				-0.05*	-0.04 (-0.25, 0.14)			
	KA	-0.49	0.04	-0.02 (-0.25, 0.17)				0.05	-0.03 (-0.22, 0.14)			

	XA	0.24	0.53*	0.13 (-0.40, 0.71)				0.18	0.04 (-0.16, 0.29)			
	AA	0.33	-0.08	-0.03 (-0.21, 0.10)				-0.03	-0.01 (-0.11, 0.06)			
	HAA	0.34	-0.11	-0.04 (-0.33, 0.20)				-0.02	-0.01 (-0.13, 0.09)			
	Pic	0.30	-0.04	-0.01 (-0.18, 0.13)				-0.05	-0.01 (-0.15, 0.10)			
	QA	16.49	0.01	0.11 (-0.13, 0.45)				0.00	0.03 (-0.07, 0.18)			
Unsaturated fat *(per SD =136 kcal/day)*	Trp	0.32	-0.09	-0.03 (-0.31, 0.19)	-0.06 (-1.02, 0.85)	1.39 (-1.11, 3.89)	1.33 (-1.27, 3.92)	-0.00	-0.00 (-0.11, 0.11)	0.09 (-0.39, 0.56)	-0.04 (-1.49, 1.41)	0.05 (-1.44, 1.54)
	Kyn	-0.03	1.06	-0.03 (-0.34, 0.23)				0.41	-0.01 (-0.20, 0.15)			
	HK	-2.81	-0.06	0.18 (-0.08, 0.59)				-0.05*	0.15 (-0.04, 0.45)			
	KA	-0.17	0.04	-0.01 (-0.25, 0.22)				0.05	-0.01 (-0.23, 0.20)			
	XA	-0.14	0.53*	-0.07 (-0.75, 0.54)				0.18	-0.03 (-0.30, 0.21)			
	AA	0.01	-0.08	-0.00 (-0.16, 0.16)				-0.03	0.00 (-0.09, 0.08)			
	HAA	0.04	-0.11	-0.00 (-0.32, 0.30)				-0.02	-0.00 (-0.12, 0.12)			
	Pic	-0.14	-0.04	0.01 (-0.16, 0.19)				-0.05	0.01 (-0.14, 0.15)			
	QA	-14.91	0.01	-0.10 (-0.47, 0.17)				0.00	-0.02 (-0.18, 0.08)			
Alcohol *(per SD =131 kcal/day)*	Trp	0.97	-0.09	-0.09 (-0.37, 0.10)	0.61 (-0.14, 1.40)	0.88 (-1.00, 2.75)	1.48 (-0.43, 3.40)	-0.00	-0.00 (-0.14, 0.12)	0.34 (-0.06, 0.76)	1.18 (-0.03, 2.39)	**1.52 (0.31, 2.73)**
	Kyn	-0.00	1.07	-0.00 (-0.18, 0.16)				0.58	-0.00 (-0.10, 0.10)			
	HK	0.03	-0.06	-0.00 (-0.22, 0.21)				-0.05*	-0.00 (-0.17, 0.17)			
	KA	3.56*	0.04	0.13 (-0.29, 0.63)				0.05	0.17 (-0.08, 0.49)			
	XA	1.46*	0.53*	**0.77 (0.18, 1.54)**				0.19	0.28 (-0.05, 0.69)			
	AA	0.10	-0.08	-0.01 (-0.13, 0.09)				-0.03	-0.00 (-0.07, 0.05)			
	HAA	-0.04	-0.11	0.00 (-0.21, 0.23)				-0.03	0.00 (-0.09, 0.09)			
	Pic	1.43	-0.04	-0.06 (-0.30, 0.14)				-0.05	-0.07 (-0.25, 0.05)			
	QA	-22.20	0.01	-0.15 (-0.50, 0.08)				0.00	-0.04 (-0.20, 0.08)			
Fiber *(per SD =12 kcal/day)*	Trp	0.97	-0.09	-0.09 (-0.39, 0.10)	-0.16 (-0.96, 0.56)	1.26 (-0.67, 3.20)	1.10 (-0.90, 3.10)	-0.00	-0.00 (-0.14, 0.13)	0.02 (-0.39, 0.40)	1.11 (-0.06, 2.27)	1.12 (-0.06, 2.31)
	Kyn	-0.03	1.07	-0.03 (-0.31, 0.20)				0.58	-0.02 (-0.18, 0.13)			
	HK	-0.63	-0.06	0.04 (-0.16, 0.29)				-0.05*	0.03 (-0.13, 0.242			
	KA	0.18	0.04	0.01 (-0.17, 0.20)				0.05	0.01 (-0.14, 0.17)			
	XA	-0.05	0.53*	-0.03 (-0.53, 0.45)				0.19	-0.01 (-0.22, 0.18)			
	AA	-0.74*	-0.08	0.06 (-0.11, 0.29)				-0.03	0.02 (-0.08, 0.15)			
	HAA	-0.07	-0.11	0.01 (-0.22, 0.25)				-0.03	0.00 (-0.09, 0.10)			
	Pic	-0.23	-0.04	0.01 (-0.12, 0.16)				-0.05	0.01 (-0.09, 0.13)			
	QA	-21.61	0.01	-0.14 (-0.49, 0.07)				0.00	-0.03 (-0.19, 0.07)			
**Micronutrient intake**												
Vitamin B2 *(per SD =0.4 mg/day)*	Trp	0.43	-0.05	-0.02 (-0.19, 0.11)	0.28 (-0.40, 0.94)	-0.66 (-2.40, 1.07)	-0.38 (-2.18, 1.41)	0.03	0.01 (-0.06, 0.12)	0.15 (-0.20, 0.50)	0.04 (-1.01, 1.10)	0.19 (-0.88, 1.26)
	Kyn	0.01	-0.49	-0.01 (-0.18, 0.15)				-0.42	-0.00 (-0.11, 0.09)			
	HK	1.02	-0.06	-0.06 (-0.29, 0.10)				-0.05*	-0.05 (-0.22, 0.08)			
	KA	2.13*	0.05	0.11 (-0.15, 0.46)				0.06	0.12 (-0.04, 0.37)			
	XA	0.79*	0.53*	**0.42 (0.00, 1.01)**				0.19	0.15 (-0.04, 0.44)			

	AA	0.17	-0.05	-0.01 (-0.13, 0.08)				-0.01	-0.00 (-0.06, 0.05)			
	HAA	1.98*	-0.11	-0.22 (-0.64, 0.07)				-0.03	-0.05 (-0.27, 0.13)			
	Pic	1.09	-0.03	-0.03 (-0.24, 0.13)				-0.04	-0.05 (-0.20, 0.05)			
	QA	13.83	0.01*	0.10 (-0.10, 0.39)				0.00	0.02 (-0.06, 0.15)			
Vitamin B6 *(per SD =0.6 mg/day)*	Trp	-0.10	-0.05	0.00 (-0.12, 0.14)	0.25 (-0.37, 0.86)	-0.17 (-1.77, 1.43)	0.08 (-1.58, 1.74)	0.03	-0.00 (-0.08, 0.07)	0.08 (-0.25, 0.40)	0.50 (-0.45, 1.45)	0.58 (-0.39, 1.54)
	Kyn	0.00	-0.42	-0.00 (-0.13, 0.13)				-0.35	-0.00 (-0.08, 0.08)			
	HK	1.10	-0.06	-0.06 (-0.29, 0.09)				-0.05*	-0.05 (-0.22, 0.06)			
	KA	1.65	0.05	0.08 (-0.13, 0.38)				0.05	0.09 (-0.04, 0.30)			
	XA	0.76*	0.53*	**0.41 (0.01, 0.96)**				0.19	0.15 (-0.03, 0.42)			
	AA	0.01	-0.05	-0.00 (-0.10, 0.09)				-0.01	-0.00 (-0.05, 0.05)			
	HAA	1.39*	-0.11	-0.16 (-0.50, 0.05)				-0.03	-0.04 (-0.21, 0.10)			
	Pic	1.46*	-0.03	-0.04 (-0.29, 0.16)				-0.05	-0.07 (-0.23, 0.05)			
	QA	3.11	0.01*	0.02 (-0.18, 0.24)				0.00	0.01 (-0.07, 0.09)			
Magnesium *(per SD =84.0 mg/day)*	Trp	0.38	-0.05	-0.02 (-0.24, 0.16)	0.13 (-0.90, 1.08)	-0.01 (-2.48, 2.45)	0.12 (-2.41, 2.65)	0.03	0.01 (-0.09, 0.14)	0.16 (-0.37, 0.68)	0.42 (-1.09, 1.94)	0.58 (-0.94, 2.11)
	Kyn	-0.04	-0.41	0.02 (-0.33, 0.39)				-0.26	0.01 (-0.20, 0.24)			
	HK	0.07	-0.06	-0.00 (-0.27, 0.26)				-0.05*	-0.00 (-0.21, 0.20)			
	KA	2.68	0.05	0.13 (-0.20, 0.60)				0.05	0.14 (-0.06, 0.47)			
	XA	0.55	0.53*	0.30 (-0.28, 1.02)				0.20	0.11 (-0.11, 0.44)			
	AA	-0.10	-0.05	0.00 (-0.13, 0.14)				-0.00	0.00 (-0.07, 0.07)			
	HAA	1.54	-0.11	-0.18 (-0.63, 0.08)				-0.03	-0.04 (-0.26, 0.11)			
	Pic	1.11	-0.03	-0.03 (-0.29, 0.15)				-0.04	-0.05 (-0.24, 0.07)			
	QA	-12.10	0.01*	-0.08 (-0.47, 0.22)				0.00	-0.02 (-0.18, 0.10)			
Zinc *(per SD =2.4 mg/day)*	Trp	0.11	-0.05	-0.01 (-0.17, 0.14)	0.48 (-0.34, 1.29)	0.12 (-1.91, 2.14)	0.59 (-1.48, 2.66)	0.03	0.00 (-0.09, 0.10)	0.15 (-0.29, 0.58)	0.59 (-0.60, 1.77)	0.73 (-0.47, 1.93)
	Kyn	0.00	-0.37	-0.00 (-0.16, 0.16)				-0.29	-0.00 (-0.10, 0.10)			
	HK	3.73*	-0.06	-0.21 (-0.63, 0.07)				-0.05*	**-0.19 (-0.48, -0.00)**			
	KA	3.41*	0.05	0.17 (-0.24, 0.66)				0.05	0.19 (-0.06, 0.53)			
	XA	1.45*	0.53*	**0.77 (0.16, 1.58)**				0.19	0.27 (-0.05, 0.70)			
	AA	0.59	-0.05	-0.03 (-0.23, 0.12)				-0.01	-0.01 (-0.11, 0.09)			
	HAA	2.06*	-0.11	-0.24 (-0.69, 0.07)				-0.03	-0.06 (-0.30, 0.14)			
	Pic	1.82*	-0.03	-0.06 (-0.35, 0.20)				-0.04	-0.08 (-0.28, 0.07)			
	QA	11.84	0.01*	0.08 (-0.14, 0.39)				0.00	0.02 (-0.07, 0.15)			
**Dietary patterns**												
DHD score *(per SD =15 points)*	Trp	0.03	-0.04	-0.00 (-0.12, 0.11)	-0.21 (-0.85, 0.38)	0.88 (-0.63, 2.40)	0.67 (-0.90, 2.24)	0.04	0.00 (-0.07, 0.08)	-0.02 (-0.35, 0.31)	0.45 (-0.47, 1.36)	0.43 (-0.51, 1.36)
	Kyn	-0.03	-0.22	0.01 (-0.22, 0.24)				-0.44	0.01 (-0.12, 0.17)			
	HK	-1.89	-0.06	0.10 (-0.05, 0.35)				-0.05*	0.09 (-0.02, 0.27)			
	KA	-0.50	0.05	-0.02 (-0.21, 0.11)				0.06	-0.03 (-0.19, 0.09)			
	XA	-0.47	0.57*	-0.27 (-0.77, 0.11)				0.22	-0.10 (-0.34, 0.05)			
	AA	-0.25	-0.05	0.01 (-0.08, 0.12)				-0.01	0.00 (-0.05, 0.06)			

	HAA	0.20	-0.13	-0.03 (-0.25, 0.16)				-0.04	-0.00 (-0.11, 0.07)			
	Pic	-0.57	-0.03	0.01 (-0.09, 0.16)				-0.04	0.02 (-0.05, 0.13)			
	QA	-4.81	0.01*	-0.03 (-0.25, 0.15)				0.00	-0.01 (-0.10, 0.06)			
WCRF/AICR *(per SD =0.7 points)*	Trp	0.05	-0.04	-0.00 (-0.11, 0.10)	-0.05 (-0.61, 0.47)	0.20 (-1.22, 1.62)	0.15 (-1.33, 1.64)	0.04	0.00 (-0.07, 0.08)	0.02 (-0.27, 0.30)	-0.11 (-0.96, 0.75)	-0.09 (-0.96, 0.79)
	Kyn	-0.01	-0.50	0.00 (-0.13, 0.15)				-0.65	0.01 (-0.07, 0.10)			
	HK	-0.12	-0.06	0.01 (-0.15, 0.17)				-0.05*	0.01 (-0.11, 0.13)			
	KA	0.40	0.05	0.02 (-0.11, 0.20)				0.06	0.02 (-0.10, 0.17)			
	XA	-0.08	0.55*	-0.05 (-0.45, 0.33)				0.21	-0.02 (-0.19, 0.13)			
	AA	-0.24	-0.05	0.01 (-0.07, 0.12)				-0.01	0.00 (-0.05, 0.06)			
	HAA	-0.16	-0.12	0.02 (-0.16, 0.22)				-0.04	0.01 (-0.07, 0.09)			
	Pic	-0.25	-0.03	0.01 (-0.08, 0.12)				-0.04	0.01 (-0.06, 0.09)			
	QA	-10.45	0.01*	-0.07 (-0.30, 0.09)				0.00	-0.02 (-0.12, 0.05)			

Abbreviations: DHD, Dutch Healthy Diet; WCRF/AICR, World Cancer Research Fund/American Institute for Cancer Research; Trp, tryptophan; Kyn, kynurenine; HK, 3-hydroxykynurenine; KA, kynurenic acid; XA, xanthurenic acid; AA, anthranilic acid; HAA, 3-hydroxyanthranilic acid; Pic, picolinic acid; QA, quinolinic acid; SD, standard deviation.

Models with macronutrients as exposure are adjusted for age, sex (male, female), renal function (μmol/L), weeks since end treatment (weeks), chemotherapy (yes, no), comorbidities (0, 1, ≥2), stoma (yes, no), educational level (low, medium, high), BMI (kg/m²), MVPA (h/week), smoking status (never, former, current), prolonged sedentary time (h/day), and energy intake using the all-components method (kcal/day). Models with micronutrients as exposure are adjusted for age, sex (male, female), renal function (μmol/L), weeks since end treatment (weeks), chemotherapy (yes, no), comorbidities (0, 1, ≥2), stoma (yes, no), educational level (low, medium, high), BMI (kg/m²), MVPA (h/week), smoking status (never, former, current), prolonged sedentary time (h/day), alcohol intake (kcal/day), and total energy intake (kcal/day). Models with dietary pattern scores as exposure are adjusted for age, sex (male, female), renal function (μmol/L), weeks since end treatment (weeks), chemotherapy (yes, no), comorbidities (0, 1, ≥2), stoma (yes, no), educational level (low, medium, high), BMI (kg/m²), MVPA (h/week), smoking status (never, former, current), prolonged sedentary time (h/day), and total energy intake (kcal/day).

* indicates a statistically significant *a*- or *b*-path ($p < 0.05$)

Bold indicates a statistically significant mediator-specific indirect effect, total indirect effect, direct effect or total effect ($p < 0.05$)

Supplementary Table 2. Analysis of the kynurenine-to-tryptophan ratio (KTR) as mediator in the longitudinal association of dietary intake with global quality of life and the EORTC summary score as outcome; single mediator model

		Global QoL (0 – 100)				Summary score (0 – 100)			
**Exposure**	**a-path**	**b-path**	**Indirect effect (a*b)**	**Direct effect**	**Total effect**	**b-path**	**Indirect effect (a*b)**	**Direct effect**	**Total effect**
**Macronutrient intake**									
Total carbohydrates *(per SD =230 kcal/day)*	2.27*	0.07	0.15 (-0.28, 0.63)	**-3.19 (-5.51, -0.86)**	**-3.03 (-5.32, -0.74)**	0.01	0.02 (-0.24, 0.30)	**-2.30 (-3.70, -0.89)**	**-2.27 (-3.65, -0.89)**
Mono- and disaccharides *(per SD =137 kcal/day)*	1.40*	0.07	0.09 (-0.18, 0.40)	**-2.12 (-3.90, -0.35)**	**-2.03 (-3.79, -0.27)**	0.01	0.01 (-0.16, 0.19)	**-1.22 (-2.29, -0.15)**	**-1.20 (-2.26, -0.15)**
Polysaccharides *(per SD =140 kcal/day)*	1.29*	0.07	0.09 (-0.17, 0.40)	-1.57 (-3.90, 0.77)	-1.48 (-3.80, 0.84)	0.01	0.01 (-0.15, 0.19)	**-1.64 (-3.04, -0.23)**	**-1.62 (-3.02, -0.23)**
Total protein *(per SD =69 kcal/day)*	0.41	0.07	0.03 (-0.09, 0.20)	1.00 (-1.13, 3.14)	1.03 (-1.11, 3.16)	0.01	0.00 (-0.07, 0.09)	0.64 (-0.62, 1.89)	0.64 (-0.61, 1.89)
Animal-based protein *(per SD =55 kcal/day)*	0.39	0.08	0.03 (-0.07, 0.19)	0.70 (-1.01, 2.40)	0.73 (-0.98, 2.43)	0.01	0.00 (-0.07, 0.08)	0.57 (-0.43, 1.57)	0.57 (-0.43, 1.57)
Plant-based protein *(per SD =33 kcal/day)*	-0.48	0.08	-0.04 (-0.27, 0.13)	**3.30 (0.49, 6.12)**	**3.27 (0.46, 6.08)**	0.01	-0.00 (-0.11, 0.09)	0.20 (-1.46, 1.86)	0.20 (-1.46, 1.86)
Total fat *(per SD =207 kcal/day)*	-1.33*	0.08	-0.09 (-0.40, 0.17)	2.13 (-0.04, 4.30)	2.04 (-0.12, 4.20)	0.01	-0.01 (-0.19, 0.15)	**1.33 (0.06, 2.60)**	**1.32 (0.06, 2.57)**
Saturated fat *(per SD =90 kcal/day)*	-0.28	0.07	-0.02 (-0.18, 0.10)	1.08 (-1.11, 3.27)	1.06 (-1.13, 3.25)	0.01	-0.00 (-0.08, 0.07)	**1.35 (0.06, 2.65)**	**1.35 (0.06, 2.64)**
Unsaturated fat *(per SD =136 kcal/day)*	-1.26*	0.07	-0.08 (-0.40, 0.17)	1.42 (-1.12, 3.96)	1.34 (-1.19, 3.86)	0.01	-0.01 (-0.18, 0.15)	0.17 (-1.29, 1.63)	0.16 (-1.29, 1.61)
Alcohol *(per SD =131 kcal/day)*	-0.73	0.07	-0.05 (-0.25, 0.10)	1.51 (-0.34, 3.37)	1.46 (-0.39, 3.32)	0.01	-0.01 (-0.12, 0.09)	**1.51 (0.31, 2.72)**	**1.50 (0.30, 2.70)**
Fiber *(per SD =12 kcal/day)*	-1.02*	0.07	-0.07 (-0.32, 0.13)	1.17 (-0.79, 3.12)	1.10 (-0.85, 3.04)	0.01	-0.01 (-0.15, 0.12)	1.15 (-0.02, 2.31)	1.14 (-0.02, 2.30)
**Micronutrient intake**									
Vitamin B2 *(per SD = 0.4 mg/day)*	0.28	0.01	0.00 (-0.10, 0.11)	-0.34 (-2.09, 1.41)	-0.34 (-2.09, 1.41)	-0.03	-0.01 (-0.09, 0.05)	0.27 (-0.78, 1.32)	0.26 (-0.79, 1.31)
Vitamin B6 *(per SD = 0.6 mg/day)*	-0.01	0.01	-0.00 (-0.08, 0.08)	0.07 (-1.55, 1.69)	0.07 (-1.55, 1.69)	-0.03	0.00 (-0.06, 0.05)	0.65 (-0.30, 1.59)	0.65 (-0.30, 1.59)
Magnesium *(per SD = 84 mg/day)*	-0.79	0.01	-0.01 (-0.22, 0.18)	0.12 (-2.35, 2.58)	0.11 (-2.35, 2.57)	-0.03	0.02 (-0.08, 0.16)	0.59 (-0.92, 2.09)	0.61 (-0.89, 2.11)
Zinc *(per SD = 2.4 mg/day)*	0.50	0.01	0.01 (-0.14, 0.15)	0.56 (-1.46, 2.58)	0.57 (-1.45, 2.59)	-0.04	-0.02 (-0.13, 0.06)	0.78 (-0.40, 1.95)	0.76 (-0.42, 1.93)
**Dietary patterns**									

DHD score *(per SD = 15 points)*	-0.77*	0.01	-0.00 (-0.18, 0.16)	0.67 (-0.87, 2.20)	0.66 (-0.87, 2.19)	-0.04	0.03 (-0.06, 0.15)	0.39 (-0.53, 1.31)	0.42 (-0.49, 1.34)
WCRF/AICR dietary score *(per SD = 0.7 points)*	-0.64	-0.00	0.00 (-0.14, 0.14)	0.15 (-1.30, 1.60)	0.15 (-1.29, 1.60)	-0.05	0.03 (-0.05, 0.13)	-0.09 (-0.95, 0.77)	-0.06 (-0.92, 0.79)

Abbreviations: KTR, kynurenine-to-tryptophan ratio; DHD, Dutch Healthy Diet; WCRF/AICR, World Cancer Research Fund/American Institute for Cancer Research; SD, standard deviation.
Models with macronutrients as exposure are adjusted for age, sex (male, female), renal function (μmol/L), weeks since end treatment (weeks), chemotherapy (yes, no), comorbidities (0, 1, ≥2), stoma (yes, no), educational level (low, medium, high), BMI (kg/m²), MVPA (h/week), smoking status (never, former, current), prolonged sedentary time (h/day), and energy intake using the all-components method (kcal/day). Models with micronutrients as exposure are adjusted for age, sex (male, female), renal function (μmol/L), weeks since end treatment (weeks), chemotherapy (yes, no), comorbidities (0, 1, ≥2), stoma (yes, no), educational level (low, medium, high), BMI (kg/m²), MVPA (h/week), smoking status (never, former, current), prolonged sedentary time (h/day), alcohol intake (kcal/day), and total energy intake (kcal/day). Models with dietary pattern scores as exposure are adjusted for age, sex (male, female), renal function (μmol/L), weeks since end treatment (weeks), chemotherapy (yes, no), comorbidities (0, 1, ≥2), stoma (yes, no), educational level (low, medium, high), BMI (kg/m²), MVPA (h/week), smoking status (never, former, current), prolonged sedentary time (h/day), and total energy intake (kcal/day).
* indicates a statistically significant *a*- or *b*-path ($p < 0.05$)
Bold indicates a statistically significant mediator-specific indirect effect, total indirect effect, direct effect or total effect ($p < 0.05$)

Supplementary Table 3. Analysis of the kynurenic acid-to-quinolinic acid (KA/QA) ratio as mediator in the longitudinal association of dietary intake with global quality of life and the EORTC summary score as outcome; single mediator model

		**Global QoL (0 – 100)**				**Summary score (0 – 100)**			
**Exposure**	**a-path**	**b-path**	**Indirect effect (a*b)**	**Direct effect**	**Total effect**	**b-path**	**Indirect effect (a*b)**	**Direct effect**	**Total effect**
**Macronutrient intake**									
Total carbohydrates *(per SD =230 kcal/day)*	-0.01*	35.36*	**-0.39 (-0.90, -0.01)**	**-2.64 (-4.95, -0.33)**	**-3.03 (-5.32, -0.74)**	32.07*	**-0.36 (-0.69, -0.10)**	**-1.94 (-3.32, -0.56)**	**-2.30 (-3.68, -0.92)**
Mono- and disaccharides *(per SD =137 kcal/day)*	-0.01*	35.19*	**-0.27 (-0.62, -0.01)**	-1.77 (-3.54, 0.00)	**-2.03 (-3.79, -0.28)**	32.36*	**-0.24 (-0.49, -0.06)**	-0.96 (-2.02, 0.10)	**-1.20 (-2.26, -0.14)**
Polysaccharides *(per SD =140 kcal/day)*	-0.01	35.19*	-0.18 (-0.53, 0.03)	-1.29 (-3.61, 1.03)	-1.47 (-3.79, 0.85)	32.36*	-0.17 (-0.43, 0.02)	**-1.51 (-2.89, -0.12)**	**-1.67 (-3.07, -0.27)**
Total protein *(per SD =69 kcal/day)*	0.01*	35.36*	0.24 (-0.00, 0.61)	0.79 (-1.35, 2.93)	1.02 (-1.11, 3.16)	32.07*	**0.22 (0.03, 0.48)**	0.44 (-0.81, 1.68)	0.65 (-0.60, 1.90)
Animal-based protein *(per SD =55 kcal/day)*	0.00*	33.17	0.16 (-0.01, 0.44)	0.56 (-1.14, 2.27)	0.72 (-0.98, 2.43)	32.23*	**0.16 (0.01, 0.36)**	0.43 (-0.57, 1.43)	0.59 (-0.41, 1.59)
Plant-based protein *(per SD =33 kcal/day)*	0.01*	33.17	0.25 (-0.03, 0.69)	3.02 (0.21, 5.83)	3.27 (0.45, 6.08)	32.23*	**0.24 (0.00, 0.57)**	-0.06 (-1.71, 1.60)	0.18 (-1.48, 1.84)
Total fat *(per SD =207 kcal/day)*	-0.00	35.36*	-0.08 (-0.35, 0.12)	2.12 (-0.03, 4.28)	2.05 (-0.11, 4.21)	32.07*	-0.07 (-0.28, 0.11)	**1.37 (0.13, 2.62)**	**1.30 (0.04, 2.56)**
Saturated fat *(per SD =90 kcal/day)*	-0.00	35.65*	-0.15 (-0.46, 0.05)	1.21 (-0.98, 3.39)	1.06 (-1.13, 3.25)	32.96*	-0.14 (-0.38, 0.04)	**1.49 (0.21, 2.77)**	**1.35 (0.06, 2.64)**
Unsaturated fat *(per SD =136 kcal/day)*	0.00	35.65*	0.05 (-0.20, 0.35)	1.29 (-1.23, 3.81)	1.34 (-1.19, 3.87)	32.96*	0.05 (-0.17, 0.29)	0.10 (-1.34, 1.54)	0.15 (-1.31, 1.60)
Alcohol *(per SD =131 kcal/day)*	0.01*	35.36*	**0.33 (0.01, 0.75)**	1.13 (-0.74, 3.00)	1.46 (-0.39, 3.31)	32.07*	**0.30 (0.09, 0.58)**	**1.20 (0.00, 2.41)**	**1.51 (0.31, 2.70)**
Fiber *(per SD =12 kcal/day)*	0.00	35.36*	0.12 (-0.06, 0.39)	0.98 (-0.96, 2.92)	1.10 (-0.84, 3.05)	32.07*	0.11 (-0.05, 0.31)	1.03 (-0.13, 2.18)	1.13 (-0.03, 2.29)
**Micronutrient intake**									
Vitamin B2 *(per SD = 0.4 mg/day)*	0.00	43.47*	0.14 (-0.05, 0.42)	-0.47 (-2.22, 1.27)	-0.33 (-2.08, 1.42)	37.21*	0.12 (-0.04, 0.33)	0.11 (-0.93, 1.16)	0.23 (-0.82, 1.29)
Vitamin B6 *(per SD = 0.6 mg/day)*	0.00	42.99*	0.14 (-0.04, 0.40)	-0.07 (-1.68, 1.55)	0.07 (-1.54, 1.69)	36.52*	0.12 (-0.03, 0.31)	0.53 (-0.41, 1.47)	0.64 (-0.30, 1.59)
Magnesium *(per SD = 84 mg/day)*	0.01*	43.45*	**0.41 (0.06, 0.91)**	-0.30 (-2.76, 2.17)	0.11 (-2.35, 2.57)	36.93*	**0.35 (0.09, 0.70)**	0.24 (-1.26, 1.74)	0.58 (-0.92, 2.09)
Zinc *(per SD = 2.4 mg/day)*	0.01*	42.35*	**0.30 (0.03, 0.69)**	0.27 (-1.76, 2.29)	0.57 (-1.45, 2.58)	36.23*	**0.26 (0.05, 0.53)**	0.49 (-0.68, 1.66)	0.75 (-0.43, 1.92)
**Dietary patterns**									

DHD score<br>*(per SD = 15 points)*	-0.00	46.72*	-0.00 (-0.21, 0.20)	0.67<br>(-0.85, 2.19)	0.67<br>(-0.86, 2.20)	40.75*	-0.00 (-0.18, 0.17)	0.44<br>(-0.47, 1.34)	0.43<br>(-0.49, 1.35)
WCRF/AICR dietary score<br>*(per SD = 0.7 points)*	0.00	46.62*	0.11 (-0.06, 0.35)	0.04<br>(-1.40, 1.48)	0.16<br>(-1.29, 1.60)	40.88*	0.10 (-0.05, 0.28)	-0.16<br>(-1.01, 0.68)	-0.06<br>(-0.92, 0.80)

Abbreviations: KA/QA, kynurenic acid-to-quinolinic acid; DHD, Dutch Healthy Diet; WCRF/AICR, World Cancer Research Fund/American Institute for Cancer Research; SD, standard deviation.

Models with macronutrients as exposure are adjusted for age, sex (male, female), renal function (µmol/L), weeks since end treatment (weeks), chemotherapy (yes, no), comorbidities (0, 1, ≥2), stoma (yes, no), educational level (low, medium, high), BMI (kg/m²), MVPA (h/week), smoking status (never, former, current), prolonged sedentary time (h/day), and energy intake using the all-components method (kcal/day). Models with micronutrients as exposure are adjusted for age, sex (male, female), renal function (µmol/L), weeks since end treatment (weeks), chemotherapy (yes, no), comorbidities (0, 1, ≥2), stoma (yes, no), educational level (low, medium, high), BMI (kg/m²), MVPA (h/week), smoking status (never, former, current), prolonged sedentary time (h/day), alcohol intake (kcal/day), and total energy intake (kcal/day). Models with dietary pattern scores as exposure are adjusted for age, sex (male, female), renal function (µmol/L), weeks since end treatment (weeks), chemotherapy (yes, no), comorbidities (0, 1, ≥2), stoma (yes, no), educational level (low, medium, high), BMI (kg/m²), MVPA (h/week), smoking status (never, former, current), prolonged sedentary time (h/day), and total energy intake (kcal/day).

* indicates a statistically significant *a*- or *b*-path ($p < 0.05$)

Bold indicates a statistically significant mediator-specific indirect effect, total indirect effect, direct effect or total effect ($p < 0.05$)

Supplementary Table 4. Analysis of the hydroxykynurenine ratio (HKr) as mediator in the longitudinal association of dietary intake with global quality of life and the EORTC summary score as outcome; single mediator model

		**Global QoL (0 – 100)**				**Summary score (0 – 100)**			
**Exposure**	**a-path**	**b-path**	**Indirect effect (a*b)**	**Direct effect**	**Total effect**	**b-path**	**Indirect effect (a*b)**	**Direct effect**	**Total effect**
**Macronutrient intake**									
Total carbohydrates *(per SD =230 kcal/day)*	0.03*	-5.36	-0.17 (-0.52, 0.09)	**-2.87 (-5.17, -0.57)**	**-3.03 (-5.32, -0.74)**	-5.08*	-0.16 (-0.39, 0.00)	**-2.10 (-3.49, -0.72)**	**-2.26 (-3.64, -0.88)**
Mono- and disaccharides *(per SD =137 kcal/day)*	-0.00	-5.68	0.00 (-0.14, 0.15)	**-2.02 (-3.78, -0.27)**	**-2.02 (-3.78, -0.27)**	-5.03*	0.00 (-0.11, 0.11)	**-1.18 (-2.24, -0.13)**	**-1.18 (-2.24, -0.12)**
Polysaccharides *(per SD =140 kcal/day)*	0.05*	-5.68	-0.28 (-0.77, 0.13)	-1.21 (-3.56, 1.14)	-1.49 (-3.81, 0.83)	-5.03*	**-0.25 (-0.55,-0.00)**	-1.40 (-2.81, 0.02)	**-1.64 (-3.04, -0.24)**
Total protein *(per SD =69 kcal/day)*	0.00	-5.36	-0.01 (-0.19, 0.15)	1.05 (-1.09, 3.18)	1.03 (-1.10, 3.17)	-5.08*	-0.01 (-0.15, 0.12)	0.67 (-0.58, 1.92)	0.66 (-0.60, 1.91)
Animal-based protein *(per SD =55 kcal/day)*	0.00	-5.29	-0.01 (-0.15, 0.12)	0.74 (-0.96, 2.44)	0.73 (-0.97, 2.44)	-5.09*	-0.01 (-0.12, 0.10)	0.60 (-0.41, 1.60)	0.59 (-0.42, 1.59)
Plant-based protein *(per SD =33 kcal/day)*	-0.00	-5.29	0.00 (-0.22, .024)	**3.26 (0.45, 6.07)**	**3.26 (0.45, 6.07)**	-5.09*	0.00 (-0.17, 0.19)	0.20 (-1.46, 1.86)	0.21 (-1.46, 1.87)
Total fat *(per SD =207 kcal/day)*	-0.02*	-5.36	0.13 (-0.08, 0.44)	1.90 (-0.27, 4.07)	2.03 (-0.13, 4.19)	-5.08*	0.12 (-0.01, 0.34)	1.13 (-0.14, 2.40)	1.25 (-0.01, 2.52)
Saturated fat *(per SD =90 kcal/day)*	-0.00	-5.41	0.02 (-0.13, 0.22)	1.04 (-1.15, 3.22)	1.06 (-1.13, 3.25)	-5.21*	0.02 (-0.11, 0.18)	**1.30 (0.01, 2.59)**	**1.32 (0.03, 2.62)**
Unsaturated fat *(per SD =136 kcal/day)*	-0.02	-5.41	0.13 (-0.08, 0.47)	1.19 (-1.34, 3.73)	1.32 (-1.21, 3.85)	-5.21*	0.12 (-0.02, 0.36)	-0.01 (-1.47, 1.45)	0.11 (-1.35, 1.57)
Alcohol *(per SD =131 kcal/day)*	-0.01	-5.36	0.07 (-0.06, 0.28)	1.40 (-0.45, 3.25)	1.46 (-0.39, 3.31)	-5.08*	0.06 (-0.03, 0.21)	**1.44 (0.24, 2.63)**	**1.50 (0.30, 2.70)**
Fiber *(per SD =12 kcal/day)*	-0.01	-5.36	0.05 (-0.08, 0.26)	1.05 (-0.90, 2.99)	1.10 (-0.85, 3.04)	-5.08*	0.05 (-0.05, 0.20)	1.10 (-0.06, 2.26)	1.15 (-0.02, 2.31)
**Micronutrient intake**									
Vitamin B2 *(per SD = 0.4 mg/day)*	-0.01	-7.03	0.05 (-0.09, 0.25)	-0.38 (-2.12, 1.37)	-0.33 (-2.08, 1.42)	-6.29*	0.04 (-0.08, 0.19)	0.21 (-0.84, 1.26)	0.25 (-0.80, 1.30)
Vitamin B6 *(per SD = 0.6 mg/day)*	-0.00	-6.98	0.00 (-0.15, 0.16)	0.06 (-1.55, 1.68)	0.06 (-1.55, 1.68)	-6.24*	0.00 (-0.12, 0.12)	0.62 (-0.33, 1.56)	0.62 (-0.33, 1.57)
Magnesium *(per SD = 84 mg/day)*	-0.02	-6.98	0.12 (-0.07, 0.44)	0.01 (-2.44, 2.47)	0.14 (-2.32, 2.60)	-6.29*	0.11 (-0.05, 0.34)	0.57 (-0.92, 2.07)	0.69 (-0.82, 2.19)
Zinc *(per SD = 2.4 mg/day)*	0.01	-7.10	-0.07 (-0.31, 0.09)	0.65 (-1.37, 2.66)	0.58 (-1.44, 2.60)	6.47*	-0.06 (-0.24, 0.08)	0.83 (-0.34, 2.00)	0.77 (-0.41, 1.94)
**Dietary patterns**									

DHD score *(per SD = 15 points)*	-0.02*	-7.08	0.14 (-0.03, 0.39)	0.53 (-1.00, 2.06)	0.67 (-0.86, 2.20)	-6.49*	**0.13 (0.01, 0.30)**	0.32 (-0.60, 1.24)	0.44 (-0.47, 1.36)
WCRF/AICR dietary score *(per SD = 0.7 points)*	-0.01	-7.35	0.07 (-0.04, 0.27)	0.08 (-1.36, 1.52)	0.15 (-1.29, 1.60)	-6.67*	0.07 (-0.03, 0.21)	-0.12 (-0.98, 0.73)	-0.06 (-0.91, 0.80)

Abbreviations: HKr, hydroxykynurenine ratio (3-hydroxykynurenine ratio : (kynurenic acid + xanthurenic acid + anthranilic acid + 3-hydroxyanthranilic acid)); DHD, Dutch Healthy Diet; WCRF/AICR, World Cancer Research Fund/American Institute for Cancer Research; SD, standard deviation.
Models with macronutrients as exposure are adjusted for age, sex (male, female), renal function (μmol/L), weeks since end treatment (weeks), chemotherapy (yes, no), comorbidities (0, 1, ≥2), stoma (yes, no), educational level (low, medium, high), BMI (kg/m²), MVPA (h/week), smoking status (never, former, current), prolonged sedentary time (h/day), and energy intake using the all-components method (kcal/day). Models with micronutrients as exposure are adjusted for age, sex (male, female), renal function (μmol/L), weeks since end treatment (weeks), chemotherapy (yes, no), comorbidities (0, 1, ≥2), stoma (yes, no), educational level (low, medium, high), BMI (kg/m²), MVPA (h/week), smoking status (never, former, current), prolonged sedentary time (h/day), alcohol intake (kcal/day), and total energy intake (kcal/day). Models with dietary pattern scores as exposure are adjusted for age, sex (male, female), renal function (μmol/L), weeks since end treatment (weeks), chemotherapy (yes, no), comorbidities (0, 1, ≥2), stoma (yes, no), educational level (low, medium, high), BMI (kg/m²), MVPA (h/week), smoking status (never, former, current), prolonged sedentary time (h/day), and total energy intake (kcal/day).
* indicates a statistically significant *a*- or *b*-path ($p < 0.05$)
Bold indicates a statistically significant mediator-specific indirect effect, total indirect effect, direct effect or total effect ($p < 0.05$)

3. Supplementary Figures

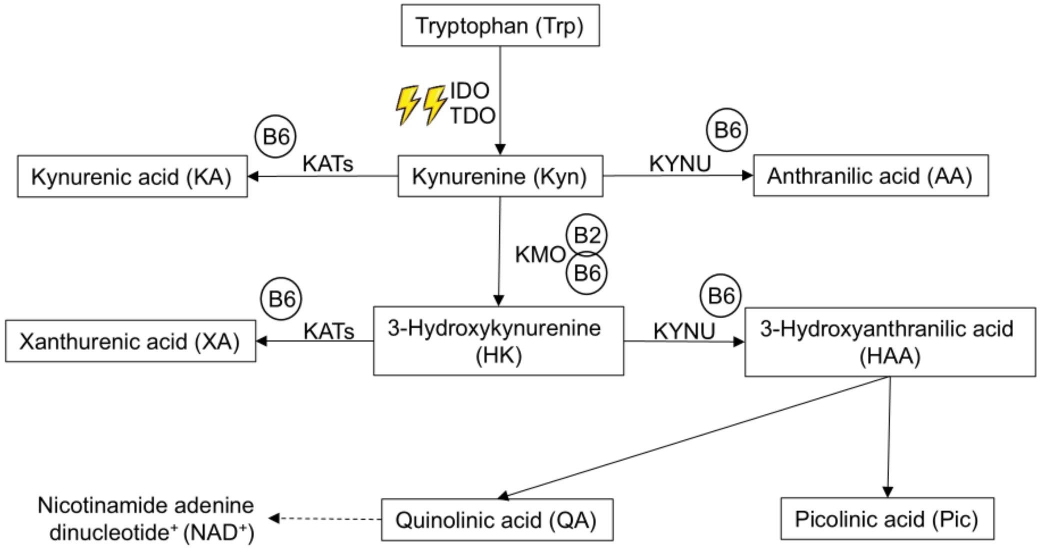

Supplementary Figure 1. The tryptophan-kynurenine pathway. The enzymes indoleamine 2,3-dioxygenase (IDO 1 & IDO 2) and tryptophan 2,3-dioxygenase (TDO) degrade Trp into Kyn. Kyn can then be further broken down into several downstream metabolites by different enzymes, including kynurenine amino transferase (KAT), kynureninase (KYNU), and kynurenine monooxygenase (KMO). Vitamin B2 and B6 are important enzymatic co-factors in the kynurenine pathway. Adapted from Holthuijsen, D. D. B. (2025). Unraveling the role of the kynurenine pathway in colorectal cancer survivorship: associations with dietary intake and domains of quality of life.

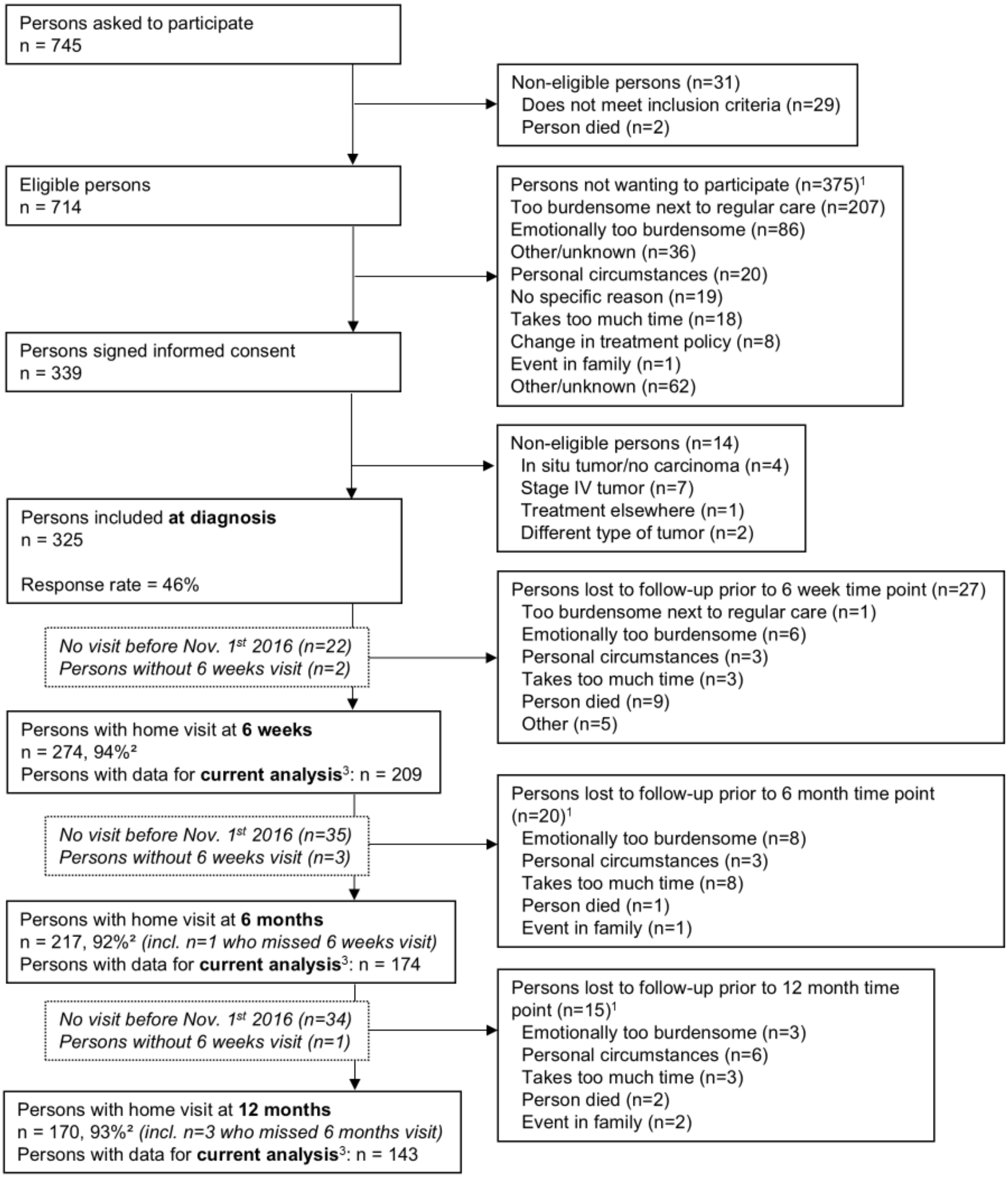

Supplementary Figure 2. Flow diagram of the inclusion of participants within the EnCoRe study and the number of posttreatment measurements included in the analyses presented in this paper. Data of home visits performed before November 1st, 2016 were included in the analyses. 1Totals do not add up because some individuals reported multiple reasons for non-participation. 2Response rate = (persons with home visits)/(persons with home visits+persons lost to follow-up – persons died). The declining number of participants at the subsequent time points is because not all participants included at diagnosis from April 2012 onwards had reached these time points in November 2016. 3Since the current analysis was focused on dietary intake, kynurenines, and HRQoL outcomes after colorectal cancer treatment, only posttreatment measurements with available data on dietary intake, kynurenines, HRQoL outcomes and covariates were included.
